# Integrated Analysis of miRNA-mRNA Interaction Network in Porcine Granulosa Cells Undergoing Oxidative Stress

**DOI:** 10.1155/2019/1041583

**Published:** 2019-11-04

**Authors:** Xing Du, Qiqi Li, Qiuyu Cao, Siqi Wang, Honglin Liu, Qifa Li

**Affiliations:** College of Animal Science and Technology, Nanjing Agricultural University, Nanjing 210095, China

## Abstract

Oxidative stress (OS), a common intracellular phenomenon induced by excess reactive oxygen species (ROS) generation, has been shown to be associated with mammalian ovarian follicular development blockage and granulosa cell (GC) impairment. However, the mechanism involved in these effects remains unknown, and the effect of OS on the transcriptome profiles in porcine GCs has not been fully characterized. In this study, we found that hydrogen peroxide-mediated oxidative stress induced porcine GC apoptosis and impaired cell viability. Moreover, RNA-seq analysis showed that oxidative stress induced dramatic changes in gene expression in porcine GCs. A total of 2025 differentially expressed genes (DEGs) were identified, including 1940 DEmRNAs and 55 DEmiRNAs. Functional annotation showed that the DEGs were mainly associated with cell states and function regulation. In addition, multiple hub genes (*FOXO1*, *SOD2*, *BMP2*, *DICER1*, *BCL2L11*, *FZD4*, *ssc-miR-424*, and *ssc-miR-27b*) were identified by constructing protein-protein interaction and DEmiRNA-DEmRNA regulatory networks. Furthermore, a gene-pathway-function coregulatory network was established and demonstrated that these hub genes were enriched in FoxO, TGF-*β*, Wnt, PIK3-Akt, MAPK, and cAMP signaling pathways, which play important roles in regulating cell apoptosis, cell proliferation, stress responses, and hormone secretion. The current research provides a comprehensive perspective of the effects of oxidative stress on porcine GCs and also identifies potential therapeutic targets for oxidative stress-induced female infertility.

## 1. Introduction

In mammalian ovaries, less than 1 percent of the follicles are mature and capable of ovulation, whereas the majority of the follicles undergo atresia and degeneration during folliculogenesis and follicular development [1]. It is generally believed that the fate of follicles is determined by the state of the follicular granulosa cells (GCs) [2, 3]. Recent reports have suggested that follicular atresia is mainly attributed to the apoptosis of GCs [4] and nonapoptotic forms of programmed cell death [5], which are mediated by a complex regulatory network that consists of multiple factors, including environmental factors [6], homeostasis [7], steroid hormones [8], cytokine [9], and epigenetic regulators [10]. Besides, accumulating evidence shows that the reactive oxygen species- (ROS-) induced oxidative stress also plays an important role in regulating the state and function of granulosa cells and even causes several anovulatory disorders [11, 12].

Oxidative stress, a common phenomenon in mammalian cells, is mainly caused by excessive ROS accumulation due to redox unbalance and involved in multiple critical biological processes [13, 14]. ROS are the natural byproducts of intracellular aerobic metabolism occurring into the mitochondria. Basel ROS concentration in normal cells can be beneficial for maintaining physiological functions, but excessive ROS accumulation disrupts cellular homeostasis and leads to oxidative stress-induced cellular damage and mitochondrial dysfunction [15–17]. It has been reported that excessive ROS levels are generated and accumulated in cells undergoing dramatic changes or processes that have a high aerobic energy metabolism requirement, such as autophagy, endoplasmic reticulum stress, carcinogenesis, and reproduction impairment [18–20]. During follicular development, metabolic rates accelerate to meet the increased demand for nutrients and energy, which inevitably generates excessive ROS and further induces oxidative stress in follicles [21]. Previous studies using hydrogen peroxide- (H_2_O_2_-) treated mouse models have shown that oxidative damage can block GC development and trigger follicular atresia [22]. However, the underlying mechanism of oxidative stress-induced GC injury and follicular atresia remains largely unknown.

In the current study, we attempted to identify the core RNA molecules and crucial pathways involved in the response of porcine GCs to oxidative stress, by constructing mRNA and miRNA expression profiles through high-throughput sequencing technology. The differentially expressed (DE) miRNA-mRNA regulatory axis and gene-pathway-function interaction network associated with H_2_O_2_-induced oxidative stress were also established. These data will lay a preliminary foundation for further investigation of the biological mechanisms of oxidative stress-induced porcine GC apoptosis and provide opportunities for the future development of molecular-targeted therapy for oxidative stress-induced female infertility.

## 2. Materials and Methods

### 2.1. Cell Culture and H_2_O_2_ Treatment

Porcine granulosa cells were derived from healthy ovarian follicles (diameter 3-6 mm) by using syringe with a 22-gauge needle and cultured into a DMEM/F12 medium with 10% fetal bovine serum (FBS) at 37°C in a 5% CO_2_ incubator as previously described [10, 23]. For H_2_O_2_ treatment, the medium was first changed by DMEM/F12 without FBS for 12 h and then H_2_O_2_ were added into the medium with the final concentration at 150 *μ*M for 3 h. The morphological features of porcine granulosa cells after H_2_O_2_ treatment were observed and recorded in [Supplementary-material supplementary-material-1]. This study was reviewed and approved by the Animal Ethics Committee of Nanjing Agricultural University, China (SYXK (Su) 2015-0656).

### 2.2. ROS Measurement

A Reactive Oxygen Species Assay Kit (#S0033, Beyotime, Haimen, China) was used to measure ROS levels in porcine GCs according to the manufacturer's instructions. Briefly, dichloro-dihydro-fluorescein diacetate (DCFH-DA) was diluted 1 : 1000 with a DMEM/F12 medium without FBS, to a concentration of 10 *μ*M. Porcine GCs were then submerged in 1 mL of DCFH-DA (10 *μ*M) for 20 min at 37°C. After incubation, cells were washed three times with a non-FBS medium and H_2_O_2_ was added to the medium for 2 h at a final concentration of 150 *μ*M. The entire procedure was performed in a darkroom. ROS levels in porcine GCs after treatment with H_2_O_2_ were detected by fluorescence microscopy and flow cytometric analysis.

### 2.3. Cell Apoptosis and Viability Analysis

To detect the apoptosis rate of porcine GC, Annexin V-FITC and propidium iodide (PI) were used according to the protocol (Vazyme Biotech Co., Ltd). Briefly, 2 × 10^5^ cells were collected and dyed with Annexin V-FITC and PI for 10 min in a darkroom, which were then sorted by flow cytometry with a cell counting machine (Becton Dickinson). FlowJo software (TreeStar) was used for data analysis. The apoptosis rate was calculated by the ratio of (cell numbers in Q2 and Q3)/total cells. For cell viability detection, Cell Counting Kit-8 (CCK-8, #K1018, APExBIO, USA) was used following the manufacturer's instructions. Briefly, porcine GCs were seeded into 96-well cell plates, and after treatment with PBS or H_2_O_2_, 10 *μ*L CCK-8 was added into the medium and incubated at 37°C for 2 h. Then, the absorbance (optical density, OD value) of porcine GCs was detected by using a microplate reader.

### 2.4. RNA Extraction, Library Preparation, and Sequencing

Porcine granulosa cells after H_2_O_2_ treatment were collected, and total RNA were extracted using the High purity RNA extraction kit (#RP1202, BioTeke Corporation, Beijing, China). The extracted RNA was run on 1.5% agarose gels to detect degradation and contamination; their quantity and quality were also estimated by the NanoDrop 3000 system (Agilent Technologies, USA). The cDNA libraries for sequencing were prepared according to the modified method [24] and subsequently sent for sequencing by Biomarker Technologies Co. Ltd., Beijing, China. The proportion of each category in relation to total clean tags was determined, and sequences obtained from *Sus Scrofa* RefSeq (*Sscrofa 11.1*) databases were used for reads mapping. The raw transcriptome sequencing data have been submitted to Sequence Read Archive (SRA) database of NCBI (accession number SUB6086396).

### 2.5. Bioinformatics Analysis

#### 2.5.1. Differentially Expressed Gene Analysis

Raw data were extracted and low-quality reads were removed through Perl scripts developed by Biomarker Technologies Co. Ltd. (Beijing, China). After quantile normalization, sequencing data were log_2_ transformed and expression levels of each gene in all samples were normalized as fragments per kilobase of transcript sequence per million mapped reads (FPKM). Differentially expressed genes (DEGs) between different samples were detected by using the DESeq R package (1.10.1), and *P* values were adjusted to control for the false discovery rate (FDR). Significant DEGs (DEmRNAs and DEmiRNAs) were identified using ∣log_2_(fold change)∣ ≥ 1 and adjusted FDR < 0.05 as cut-off criteria.

#### 2.5.2. Functional Annotation of Differentially Expressed Genes

To assess the functions, roles, and biological processes of the DEGs and their enrichment in different biological pathways, Gene Ontology (GO) and Kyoto Encyclopedia of Genes and Genomes (KEGG) pathway enrichment analyses were performed by using online database, DAVID (the Database for Annotation, Visualization and Integrated Discovery, version 6.7, https://david.ncifcrf.gov/). A significance level of *P* < 0.05 and an enrichment score > 2 were set as the thresholds. For the functional annotation and miRNA/pathway clustering of DEmiRNAs, the DIANA-miRPath v3.0 database (http://www.microrna.gr/miRPathv3/) was used according to the published instructions [25].

#### 2.5.3. Protein-Protein Interaction Network Construction

DEG-encoded proteins were chosen for construction of a protein-protein interaction (PPI) network. Briefly, potential or confirmed protein interactions were generated and analyzed using STRING online software (http://string-db.org/), with a minimum confident interaction score > 0.9 (0-1) and the interacted protein amount ≥ 1. The PPI network was then visualized using Cytoscape v3.7.1 software. Hub genes were identified as the nodes with higher degrees (top 5%) using CytoHubba functions. The Cytoscape software MCODE package was performed to analyze the modules in the PPI network.

#### 2.5.4. Construction of DEmiRNA-DEmRNA Regulatory Network and Functional Assessment

Target genes of DEmiRNAs were first predicted using the miRWalk v3.0 database (http://mirwalk.umm.uni-heidelberg.de/search_mirnas/), microRNA.org (http://microrna.org/), TargetScan (http://www.targetscan.org/), and miRDB (http://www.mirdb.org/). The common genes both belong to DEmiRNA targets and DEmRNAs which have inverse expression relationship with DEmiRNAs were chosen to analyze miRNA-mRNA pairs and retained for DEmiRNA-DEmRNA regulatory network construction through using Cytoscape software. To assess the functions of these miRNA-mRNA regulatory networks, DAVID was used for GO annotation and KEGG pathway analysis of the differentially expressed target genes. Venn diagram indicating the intersected genes was generated by a Draw Venn Diagram online tool (http://bioinformatics.psb.ugent.be/webtools/Venn/). Hub miRNAs were defined as the miRNA nodes with higher degree (top 5%) in the DEmiRNA-DEmRNA regulatory network.

### 2.6. Quantitative Real-Time PCR Validation

Total RNA from porcine granulosa cells after H_2_O_2_ treatment was reverse-transcribed into cDNA with three biological repeats by using HiScript® III RT SuperMix for qPCR (+gDNA wiper, #R323-01, Vazyme Biotech Co., Ltd.) according to the manufacturer's instructions. Several significant DEGs were chosen for sequencing accuracy detection, and qRT-PCR were performed by using AceQ qPCR SYBR Green Master Mix (#Q111-03, Vazyme Biotech Co., Ltd, Nanjing, China) on a StepOne Plus System (Applied Biosystems) with three biological repeats. The experimental data were analyzed using the 2^-*ΔΔ*CT^ method. The expression levels of coding genes were normalized to that of *GAPDH*. *U6* was chosen as an internal control of miRNAs' expression levels. The primers used here were designed using the primer 5.0 software and listed in Supplementary [Supplementary-material supplementary-material-1].

### 2.7. Statistical and Data Analysis

Statistical analyses were performed using GraphPad Prism 7.0 (GraphPad Software, CA, USA) and SPSS 20.0 (SPSS, IL, USA). The comparisons were conducted by a two-tailed Student's *t*-test. *P* value < 0.05 was considered as statistically significant.

## 3. Results

### 3.1. H_2_O_2_ Induced Oxidative Stress in Porcine GCs

In this study, 150 *μ*M H_2_O_2_ was used to establish oxidative stress in a porcine GC model. To confirm that the model was constructed successfully, we first analyzed ROS levels in porcine GCs under different treatment conditions ([Supplementary-material supplementary-material-1]). ROS levels in porcine GCs treated with 150 *μ*M H_2_O_2_ were significantly upregulated and higher than that in the control group (PBS), indicating that excessive ROS were generated and accumulated after 150 *μ*M H_2_O_2_ treatment. Besides, we observed that H_2_O_2_-treated porcine GCs had shrunken appearance with jagged edges, suggesting the loss of membrane integrity and low cell viability of porcine GCs ([Supplementary-material supplementary-material-1]). In addition, H_2_O_2_-mediated oxidative stress significantly upregulated porcine GC apoptosis ([Supplementary-material supplementary-material-1]) and dramatically inhibited cell viability ([Supplementary-material supplementary-material-1]). These observations suggested that 150 *μ*M H_2_O_2_ could induce oxidative stress in porcine GCs.

### 3.2. Identification of Differentially Expressed RNAs in Porcine GCs Treated with H_2_O_2_

**Figure 1:**
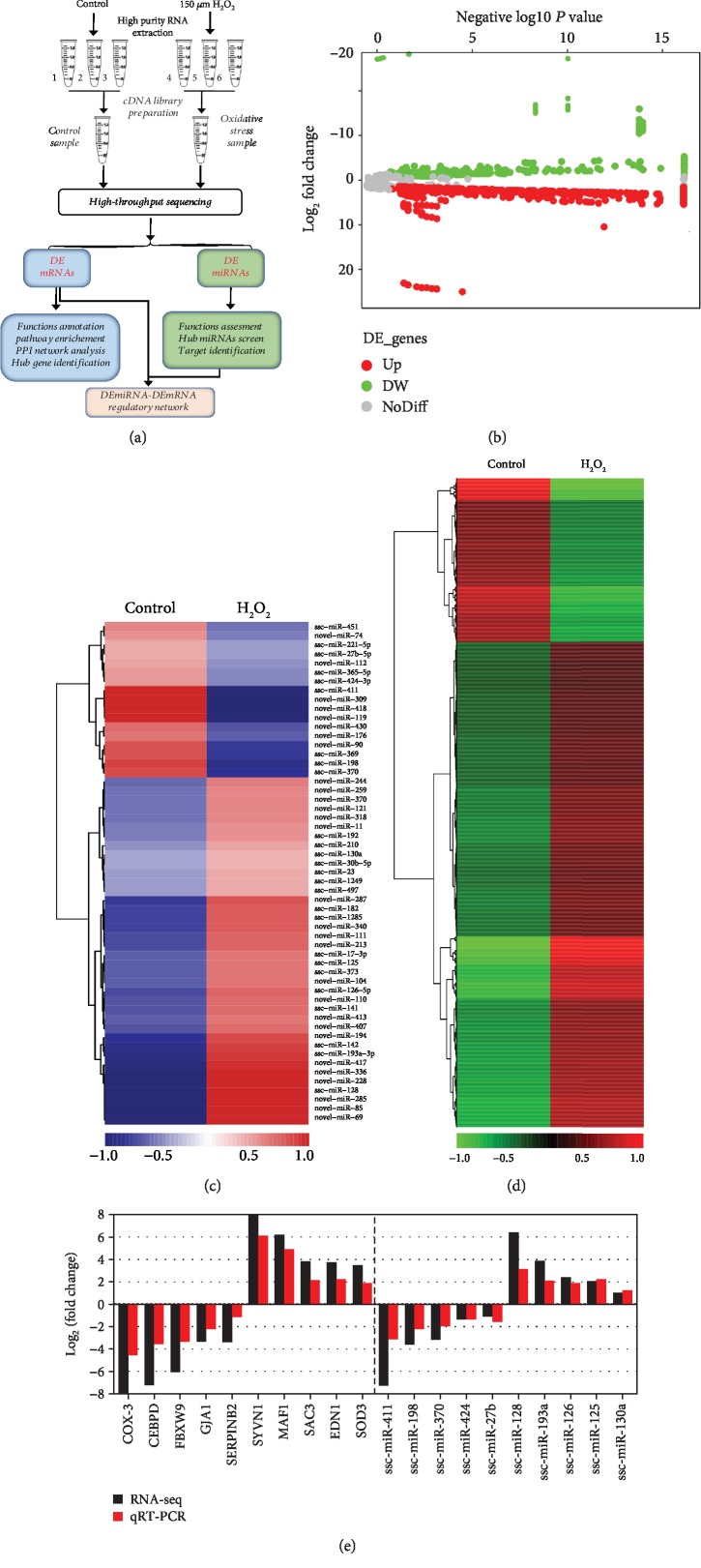
Expression profiles of differentially expressed RNAs in porcine GCs under oxidative stress. (a) Flow diagram showing the strategy for detection of differentially expressed RNAs through RNA-seq technology. (b) Volcano plot of differentially expressed RNAs in porcine GCs treated with H_2_O_2_. Up- and downregulated genes are indicated as red and green points, respectively (fold change ≥ 2 and FDR < 0.05). (c) Heat map showing the relative expression patterns of 55 DEmiRNAs between control and H_2_O_2_ treatment groups. The color scale of the heat map ranges from blue (low expression) to red (high expression). (d) Hierarchical clustering of 1970 DEmRNAs in porcine GCs under oxidative stress. Color brightness reflects the degree of expression, increase (red) or decrease (green). (e) Differentially expressed genes with high fold change were chosen for qRT-PCR validation of transcriptomic results in the porcine GCs under oxidative stress. The values are shown as log_2_ (fold change).

To investigate the crucial RNA molecules and pathways involved in the responses of porcine GCs to oxidative stress, a high-throughput sequencing strategy was employed, as shown in Figure 1(a). Using the criteria of ∣log_2_(fold change)∣ ≥ 1 and adjusted FDR < 0.05, a total of 2025 DEGs were identified in H_2_O_2_-treatment porcine GCs compared to the control group (Figure 1(b)), including 1970 DEmRNAs (1474 up- and 496 downregulated, Supplementary [Supplementary-material supplementary-material-1]) and 55 DEmiRNAs (38 up- and 17 downregulated, Supplementary [Supplementary-material supplementary-material-1]). Besides, 284 novel and 600 function-unknown genes were identified in the sequencing data. In addition, heat maps of these DEGs were generated with hierarchy cluster analysis to show their expression patterns (Figures 1(c) and 1(d)). The top 10 most up- and downregulated DEmRNAs and DEmiRNAs according to fold change are presented in Tables 1 and 2, respectively. Among these, *SYVN1* and *COX-3* were the most up- and downregulated mRNAs, whereas novel-miR-336 and novel-miR-418 were the most up- and downregulated miRNAs, respectively. To validate the accuracy of the sequencing data, top 10 significantly changed DEmRNAs and DEmiRNAs were selected for qRT-PCR detection and as shown in Figure 1(e), the changes of their expression level after H_2_O_2_ treatment were generally similar in qRT-PCR and sequencing data, indicating the high accuracy of our sequencing analysis.

### 3.3. Functional Analysis of Differentially Expressed mRNAs

**Figure 2:**
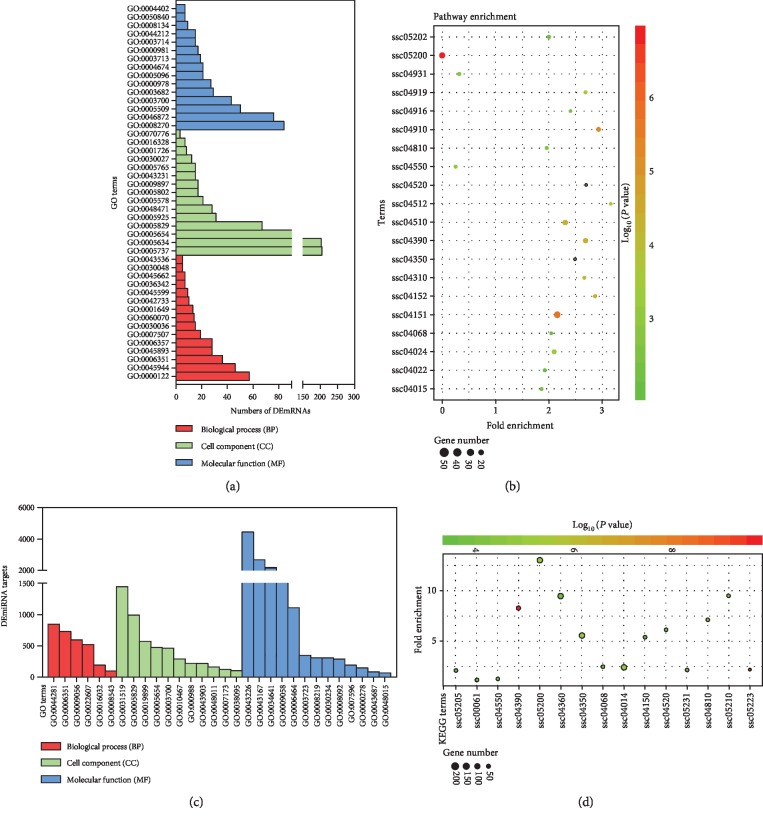
Functional analysis of differentially expressed RNAs. (a, b) Gene Ontology analysis (a) and KEGG pathway enrichment analysis (b) of DEmRNAs in porcine GCs treated with H_2_O_2_. (c, d) Functional annotations of DEmiRNAs were performed using Gene Ontology (c) and KEGG pathway enrichment analysis (d). GO and KEGG pathway terms with *P* value < 0.05 were considered as significantly enriched. The corresponding terms are listed in Supplementary [Supplementary-material supplementary-material-1].

To further investigate the role of these DEGs in porcine GCs under oxidative stress, Gene Ontology (GO) analysis was performed to assess the function of 1270 function-known DEmRNAs using DAVID. This analysis identified 135 significantly altered GO terms (*P* < 0.05, Supplementary [Supplementary-material supplementary-material-1]). As shown in Figure 2(a), three GO categories, including biological process (BP), cell component (CC), and molecular function (MF), were analyzed. The three most enriched GO terms in the BP category were “negative regulation of transcription from RNA polymerase II promoter,” “positive regulation of transcription from RNA polymerase II promoter,” and “heart development.” In CC category, “nucleoplasm,” “nucleus,” and “cytoplasm” were the three most enriched GO terms, whereas the top three terms in MF were “zinc ion binding,” “transcription coactivator activity,” and “GTPase activator activity.” Furthermore, KEGG pathway enrichment analyses showed that these DEmRNAs were mainly enriched in pathways associated with regulation of cell state and functions, such as PI3K-Akt, AMPK, cAMP, TGF-*β*, and FoxO signaling pathways (Supplementary [Supplementary-material supplementary-material-1]). The top 20 of the 38 significantly enriched pathways (*P* < 0.05) is shown in Figure 2(b).

### 3.4. Functional Annotation of Differentially Expressed miRNAs

To investigate the functions of these 55 DEmiRNAs in porcine GCs treated with H_2_O_2_, their targets were first predicted and GO analysis was performed. As shown in Figure 2(c), three GO categories including biological process, cell component, and molecular function were analyzed. In BP, the most enriched GO term was “small molecule metabolic process”. In CC, “protein complex” was the most enriched GO term and “ion binding” was the most enriched GO term in MF (Supplementary [Supplementary-material supplementary-material-1]). In addition, KEGG pathway enrichment analysis was performed and showed that the targets of DEmiRNAs were mainly enriched in estrogen generation, Hippo, TGF-*β*, FoxO, Ras, and mTOR signaling pathways (Figure 2(d); Supplementary [Supplementary-material supplementary-material-1]). Moreover, DEmiRNA annotation and miRNA/pathway clustering were performed and showed that these DEmiRNAs mainly participated in regulating cell pluripotency, cell growth and death, fatty acid metabolism, estrogen biosynthesis, and diseases ([Supplementary-material supplementary-material-1]).

### 3.5. DEG Protein-Protein Interaction Analysis and Hub Gene Identification

**Figure 3:**
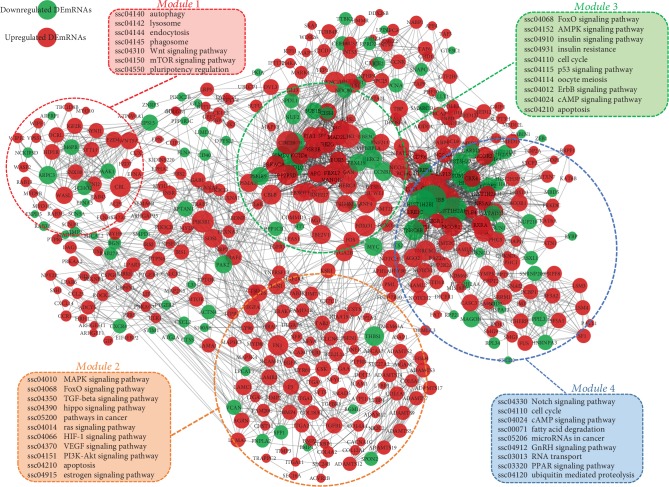
Protein-protein interaction (PPI) network of DEmRNAs and module analysis. A total of 483 function-known DEmRNAs existed in the PPI network. The color and the size of nodes represent their direction of expression change and degree, respectively. The circled areas indicate the four most significant modules. Pathways that each module are involved in were analyzed by KEGG pathway enrichment analysis.

After removing the novel and function-unknown DEGs, a PPI network was built and visualized by using STRING online database and the Cytoscape v3.7.1 visualization tool. As shown in Figure 3, there were 483 nodes (380 up- and 103 downregulated genes) and 2499 edges in the PPI network. Among them, 30 nodes with higher degree (top 5%) were considered as hub genes with *CREBBP*, *HIST1H2BD*, *CDK1*, *CDC20*, *PIK3R1*, *FOXO1*, *DYNC1H1*, *SUMO1*, *CBL*, and *FN1* being the most significant 10 node degree genes (Supplementary [Supplementary-material supplementary-material-1]). Module analysis was conducted and showed that there were four significant modules existing in the PPI network (Figure 3). KEGG pathway analyses of the genes in these modules showed that they were mainly enriched in pathways involved in regulating the cell cycle, growth, apoptosis, autophagy, oxidative stress, and ubiquitin modification. The top 6 hub genes were selected for qRT-PCR validation. The qRT-PCR and sequencing results were highly consistent, except for the *SUMO1* gene that was not significantly affected by H_2_O_2_ treatment ([Supplementary-material supplementary-material-1]).

### 3.6. DEmiRNA-DEmRNA Regulatory Network and Functional Assessment

**Figure 4:**
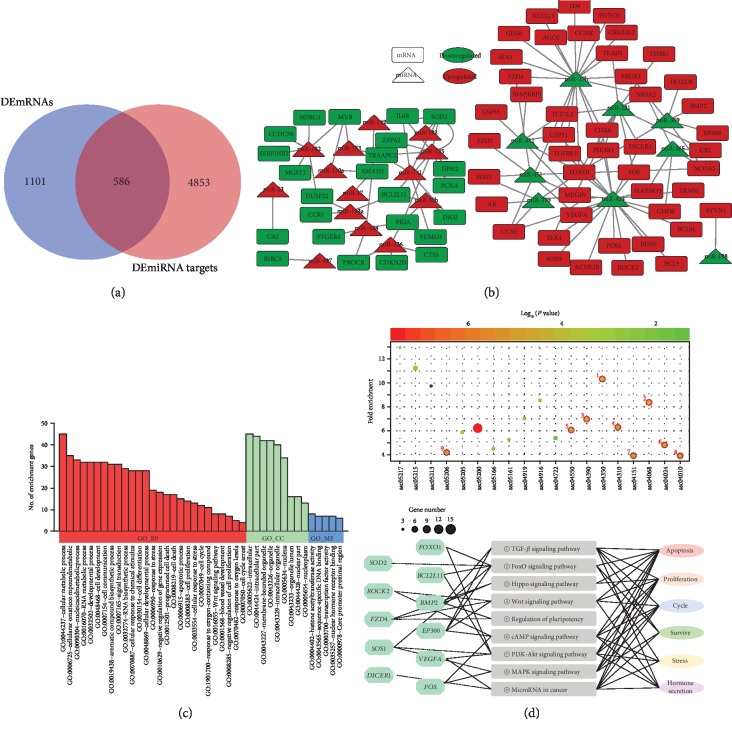
DEmiRNA-DEmRNA regulatory network analysis. (a) Venn diagram showing the overlapping genes that simultaneously belong to the DEmRNAs and the predicted target genes of the DEmiRNAs. (b) DEmiRNA-DEmRNA regulatory network. Rectangles and triangles indicate DEmRNAs and DEmiRNAs, respectively. Red and green indicate up- and downregulated, respectively. (c) Gene Ontology (GO) analysis for DEmiRNA-DEmRNA regulatory axes in porcine GCs underlying oxidative stress. (d) KEGG pathway enrichment analysis for DEmiRNA-DEmRNA regulatory axes (upper) and gene-pathway-function coregulation network was established (lower).

To establish the DEmiRNA-DEmRNA regulatory network, the potential targets of DEmiRNAs were first analyzed and 5439 genes were identified, including 586 common genes with DEmRNAs in the sequencing data (Figure 4(a)). With the DEmiRNAs (14 up- and 9 downregulated) and common DEmRNAs (46 up- and 24 downregulated) mentioned above, a regulatory network was established including 93 nodes and 124 edges (Figure 4(b), Supplementary [Supplementary-material supplementary-material-1]). In this work, ssc-miR-424 and ssc-miR-27b with higher degrees (top 5%) were considered as hub miRNAs. Functional assessment showed that their targets were mainly enriched in TGF-*β*, FoxO, Hippo, Wnt, cAMP, PI3K-Akt, and MAPK signaling pathways (Figure 4(c) upper lane). To further assess the effects of the miRNA-mRNA regulatory network on porcine GCs, gene-pathway-function coexpression patterns were analyzed and indicated that the oxidative stress-induced miRNA-mRNA regulatory axes exerted important roles in regulating states (proliferation, survive, and apoptosis) and functions (stress responses and hormone secretion) of porcine GCs (Figure 4(c) lower lane).

## 4. Discussion

Ovarian follicle development is a complex process that has been proved to be regulated by multiple follicular fluid factors, such as FSH, IGF-1, and ROS [26–28]. Previous studies have shown that ROS levels in follicular fluid are closely related to follicular development, atresia, and ovarian diseases. For example, high levels of ROS-induced oxidative stress increase HCG-stimulated androgen secretion and contribute to polycystic ovary syndrome (PCOS) [29]. Besides, excess ROS induces autophagy in oocytes, which eventually leads to follicular atresia and premature ovarian failure [30, 31]. 3-NP has been used as an oxidant in several studies and proves that it causes excess ROS generation and induces mouse follicular atresia and mouse GC (mGC) apoptosis *in vivo* [32, 33]. Meanwhile, proanthocyanidins and gallic acid have been shown to be excellent antioxidants, which further decrease the follicular ROS levels in mice and suppress atresia by inhibiting mGC apoptosis [34]. *In vitro*, H_2_O_2_-mediated oxidative stress has been proved to significantly increase mGC apoptosis and impair relative functions [26, 35]. In the present study, we found that 150 *μ*M H_2_O_2_ induced excess ROS generation and oxidative stress in porcine GCs which further induced porcine GC apoptosis and inhibited cell viability *in vitro*.

Accumulating evidence obtained through sequencing technology has shown that oxidative stress significantly changes gene expression in multiple cell types of different species, such as bovine, mouse, and human [36, 37]. In this study, DEmRNAs were screened and a total of 1970 DEmRNAs were identified in porcine GCs treated with H_2_O_2_. Moreover, multiple hub genes including *FOXO1*, *SOD2*, *COX-3*, *BMP2*, and *FZD4* were identified. Among which, FOXO1, a sensor of ROS, has been proven to be regulated by oxidative stress at different levels [38, 39]. Members of TGF-*β* (BMP2) and Wnt signaling pathway (TCF7 and FZD4) have also been proven to be regulated by oxidative stress in human bone marrow stromal cells (hBMSCs) [40]. In addition, the expression and activity of SOD2 have been reported to be modulated by oxidative stress during mitophagy and vascular hypertension [41, 42]. GO and KEGG pathway enrichment analyses demonstrated that these DEmRNAs mainly serve as regulators of porcine GC states and functions. For instance, *CDK6*, *CDK18*, *CDKN2B*, *CDKN2C*, *CCNB1*, *CCNJ*, and *CCNK* are associated with cell cycle [43–45]. *BMP2*, *GDF15*, *TGF-β1*, *SMAD5*, and *PCNA* are involved in cell proliferation [46, 47]. *BCL2L11*, *FOXO1*, *FOXO3*, *MMP2*, and *SOS1* are apoptotic factors [48, 49]. *ACVR2B*, *BMP1*, *BMP4*, and *NR5A2* are associated with hormone secretion and cytokine responses [50, 51]. These identified DEmRNAs may partially explain the effects of oxidative stress on porcine GC states and functions. Apart from these well-known genes, many function-unknown DEmRNAs were also identified and their functions will be the focus of future investigations.

MicroRNAs (miRNAs), one of the most important endogenous epigenetic factors, are a class of 18~24 nt short noncoding RNA that inhibit gene expression at the posttranscriptional level by complementary binding to the 3′-untranslated regions (3′-UTR) of specific target genes [52–54]. Thus, miRNAs are involved in regulating numerous biological processes such as cell death, proliferation, autophagy, and various diseases [55–57]. Importantly, miRNAs have been shown to be the major mediators affecting cell function under conditions of oxidative stress [58, 59]. For example, oxidative stress significantly upregulated various miRNAs, including miR-30b, miR-194, miR-125, and miR-128 which further suppressed proliferation of human fibroblasts and HN cells [59, 60]. Besides, Lee et al. showed that oxidative stress-induced hnRNPA2B1 increases *miR-17/93* expression in epithelial cells and elicits an innate immune response [61]. In addition, oxidative stress-induced APE1 is required for *miR-221/222* processing and regulating the tumor suppressor, PTEN [62]. A previous study using high-throughput sequencing technology showed that oxidative stress modulates the expression of miRNAs in bovine GCs [63]. In the present study, we identified 55 DEmiRNAs (38 up- and 17 downregulated) in oxidative stress porcine GCs through RNA-seq with the criteria ∣log_2_(fold change)∣ ≥ 1 and FDR < 0.05. Interestingly, we observed that the miRNAs mentioned above also existed in our DEmiRNA library. Apart from these well-known miRNAs, multiple newly identified OS-induced DEmiRNAs may also play vital roles in ovaries. miR-126 has been shown to induce GC apoptosis by directly targeting *FSHR* in pigs [64]. miR-142 regulates the proliferation and apoptosis of human GC by inhibiting *TGFBR1* [65]. The miR-182/183/96 cluster actively participates in sexual differentiation in primordial germ cells [66]. Additionally, in another ongoing study, we showed that miR-130a significantly induces porcine GC apoptosis and follicular atresia by targeting the TGF-*β* signaling pathway (data not shown). Moreover, several DEmiRNAs, such as miR-141 and miR-210, have been proven to control oxidative stress responses in ovarian cancer cells and endometriotic cells by targeting *p38α* and *BARD1*, respectively [67, 68], suggesting that several identified DEmiRNAs may function as modulators in response to oxidative stress. We also identified 19 novel, function-unknown DEmiRNAs which require further investigation. It is worth noting that *DROSHA* and *DICRE1* (miRNA-processing enzyme encoding genes) were significantly upregulated during this process which may partially explain the multiple miRNAs differentially expressed in porcine GCs undergoing oxidative stress.

Previous studies have demonstrated that oxidative stress often induces multiple signaling pathways during the regulation of different cellular biological processes. These mainly include the FOXO [26], TGF-*β* [13], Wnt [69], Hippo [70], and PI3K-Akt signaling pathways [71], which were also enriched in the miRNA-mRNA pathway-function regulatory network. Furthermore, cAMP signaling, MAPK signaling, regulation of pluripotency, and miRNAs in cancer were also enriched. Therefore, we hypothesized that oxidative stress might participate in regulating the morphology, pluripotency, energy metabolism, and small noncoding RNA processing in porcine GCs, which requires confirmation in future research. Although the expression profiles of RNAs (mRNAs and miRNAs) were discussed and multiple differentially expressed RNAs were verified by qRT-PCR in the present study, further studies are required to support the results *in vivo*. Moreover, the mechanism involved in the differential expression of RNAs in responses to oxidative stress and how their interaction network affects the state and function of porcine GCs needs to be more precisely described in the future.

## 5. Conclusion

**Figure 5:**
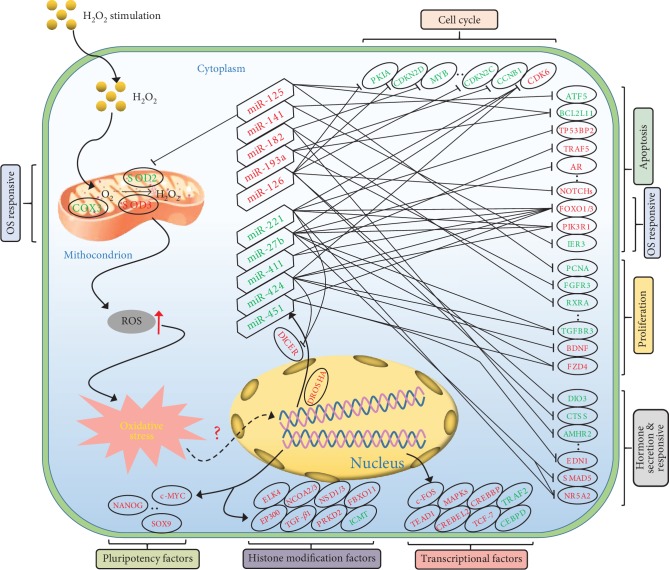
Proposed model of the porcine GC response to H_2_O_2_-mediated oxidative stress. Exogenous H_2_O_2_ induced ROS generation and accumulation in porcine GCs, which further incurred oxidative stress and dramatic changes of transcriptome. DEmiRNA-DEmRNA-function interaction network in porcine GCs undergoing oxidative stress proofed previously or predicted was presented. Red fonts indicate upregulation and green fonts indicate downregulation. Hexagons represent DEmiRNAs and ovals represent DEmRNAs. Moreover, multiple important factors involved in cell pluripotency, histone modification, and gene transcription were differentially expressed after H_2_O_2_ treatment.

In summary, we constructed differentially expressed RNA profiles using RNA-seq technology in this study and demonstrated that dramatic changes in gene expression occurred in porcine GCs under oxidative stress. Functional annotation analysis showed that these DEGs were mainly involved in regulating the state and function of porcine GCs, which was highly consistent with our observations that oxidative stress significantly induced porcine GC apoptosis and dramatically impaired cell viability. A DEmiRNA-DEmRNA pathway-function coregulatory network and a PPI network were established and indicated that multiple physiological processes and signaling pathways were involved in the response of porcine GCs to oxidative stress (Figure 5). The integrated analysis of miRNA-mRNA interaction networks also provides a series of potential therapeutic targets for oxidative stress-induced female infertility.

## Figures and Tables

**Table 1:** Top 10 up- and downregulated DEmRNAs in porcine GCs treated with H_2_O_2_.

Ensembl ID	Gene symbol	Log_2_FC	FDR	Chr.^1^	Regulation
ENSSSCG00000027057	*SYVN1*	9.906	3.23*E*-56	2	Up
New gene_46815	*—*	8.131	2.25*E*-35	2	Up
ENSSSCG00000032395	*—*	7.797	7.74*E*-31	6	Up
ENSSSCG00000040186	*C12ORF57*	7.731	7.23*E*-30	12	Up
ENSSSCG00000029474	*AVEN*	7.132	6.88*E*-28	7	Up
New gene_79274	*—*	6.666	5.23*E*-26	4	Up
ENSSSCG00000017551	*FAM117A*	6.268	9.25*E*-25	12	Up
ENSSSCG00000005915	*MAF1*	6.183	1.01*E*-23	4	Up
ENSSSCG00000021899	*—*	6.100	9.95*E*-21	6	Up
New gene_76434	*—*	5.899	2.23*E*-20	4	Up
ENSSSCG00000040167	*—*	-5.935	1.09*E*-21	18	Down
ENSSSCG00000013725	*FBXW9*	-6.045	8.09*E*-21	2	Down
New gene_34816	*—*	-6.213	1.33*E*-21	17	Down
ENSSSCG00000028892	*—*	-6.337	2.38*E*-23	6	Down
New gene_126488	*—*	-6.512	8.28*E*-25	8	Down
ENSSSCG00000036469	*HIST2H3PS2*	-6.642	5.53*E*-26	1	Down
ENSSSCG00000036620	*DPH2*	-6.979	5.99*E*-27	6	Down
ENSSSCG00000032367	*CEBPD*	-7.205	7.22*E*-29	4	Down
ENSSSCG00000012319	*—*	-9.126	1.19*E*-50	X	Down
ENSSCG00000018082	*COX-3*	-9.445	1.43*E*-53	MT	Down

^1^Chr. indicates chromosome.

**Table 2:** Top 10 up- and downregulated DEmiRNAs in porcine GCs treated with H_2_O_2_.

miRNAs	Log_2_FC	FDR	Mature sequence (5′-3′)	Regulation
novel-miR-336	7.055	5.39*E*-4	UCCCUGGCCUGGGAACUUUU	Up
novel-miR-228	6.557	8.02*E*-4	GCGGGACUGUGCAACUUGCUUUGAC	Up
ssc-miR-128	6.433	1.34*E*-2	CGGGGCGGCAGGCUGAGCCU	Up
novel-miR-285	6.297	2.28*E*-2	UCUCUCCCCCUCCGUCCCAGG	Up
novel-miR-69	6.147	4.00*E*-2	UCUCCAGCCAGACCAGAGGAU	Up
novel-miR-85	6.147	4.00*E*-2	AGGGAGGGUUUGGGUUCAUCUGU	Up
novel-miR-417	5.210	1.04*E*-8	GUGGCUGAGGUGAGAACA	Up
ssc-miR-193a	3.845	3.53*E*-3	AACUGGCCUACAAAGUCCCAGU	Up
ssc-miR-142	3.758	5.58*E*-3	CUCCCAGCGGUGCCUCCU	Up
novel-miR-194	3.347	3.93*E*-2	GUAUGUGAGCGGGGGGCUGGUGGG	Up
novel-miR-176	-2.153	2.82*E*-5	AGACCUUGAUGGCUGGCUGAGUCUC	Down
novel-miR-430	-2.337	3.29*E*-3	GUUAACGAAUCUGACUAGG	Down
ssc-miR-369	-2.818	4.70*E*-2	AGUGGGCUGAGGAUCUGGCGUUGU	Down
novel-miR-90	-2.938	5.62*E*-14	UGGUUUGUUUGGGUUUGUU	Down
ssc-miR-370	-3.178	8.29*E*-4	GCCUGCUGGGGUGGAACCUGGU	Down
ssc-miR-198	-3.606	2.17*E*-13	UAGUGGCUAGGAUUCGGCG	Down
novel-miR-309	-6.759	4.56*E*-4	AUGGUGAGUGUGGACGUG	Down
novel-miR-119	-6.759	4.56*E*-4	GCCUUGAAGACUUUGGCA	Down
ssc-miR-411	-7.245	7.21*E*-29	GGGCCUGUGGCUCAGAGGG	Down
novel-miR-418	-7.858	1.10*E*-6	GCCGUGGAGACCUGGGCC	Down

## Data Availability

The data used to support the findings of this study are included within the article and supplementary information file.

## Supplementary Materials

Supplementary Materials Figure 1S: oxidative stress impaired porcine granulosa cells. (a) ROS levels in porcine GCs treated with 150 *μ*M H_2_O_2_ were detected by fluorescence microscopy (upper lane) and flow cytometry analysis (lower lane). (b) The morphological features of porcine GCs after 150 *μ*M H_2_O_2_ treatment were observed and recorded by a stereomicroscope. (c) Cell viability was measured in control (PBS) and oxidative stress groups (H_2_O_2_) after treatment for 2 h. (d) The apoptosis rate of porcine GCs treated with H_2_O_2_ was detected by flow cytometry analysis. Data are represented as mean ± S.E.M. ^∗∗^*P* < 0.01 with two-tailed Student's *t*-test. Figure 2S: functional annotation of DEmiRNAs in porcine GCs under oxidative stress, related to Figure 2. (a) The heat map depicting the Gene Ontology (GO) enrichment analyses of DEmiRNAs in porcine GCs treated with 150 *μ*M H_2_O_2_. Clustering analyses were performed at both DEmiRNAs and GO term levels. (b) DEmiRNA/KEGG pathway clustering was analyzed in porcine GCs undergoing oxidative stress. 11 significant enrichment signaling pathways existed in the heat map. Figure 3S: hub gene expression validation, related to Figure 3. The expression levels of 6 hub genes were verified by qRT-PCR. Black columns indicate data from RNA-seq, and red columns indicate qRT-PCR data. Supplementary Table S1: primers used for qRT-PCR in this study. Supplementary Table S2: differentially expressed mRNAs in pGCs treated with H_2_O_2_. Supplementary Table S3: differentially expressed miRNAs in pGCs treated with H_2_O_2_. Supplementary Table S4: GO enrichment analysis of DEmRNAs after H_2_O_2_ treatment. Supplementary Table S5: KEGG pathway analysis of DEmRNAs after H_2_O_2_ treatment. Supplementary Table S6: GO enrichment analysis of DEmiRNAs after H_2_O_2_ treatment. Supplementary Table S7: KEGG pathway analysis of DEmiRNAs after H_2_O_2_ treatment. Supplementary Table S8: hub genes in the protein-protein interaction network. Supplementary Table S9: hub genes and miRNAs in the miRNA-mRNA interaction network.
