# Supplementary material for: Integrated Analysis of miRNA-mRNA Interaction Network in Porcine Granulosa Cells Undergoing Oxidative Stress
Source: Oxid Med Cell Longev. 2019 Nov 4;2019:1041583. doi: 10.1155/2019/1041583 (PMC6875397; doi:10.1155/2019/1041583)
Supplement: Supplementary Materials — Figure 1S: oxidative stress impaired porcine granulosa cells. (a) ROS levels in porcine GCs treated with 150 μM H2O2 were detected by fluorescence microscopy (upper lane) and flow cytometry analysis (lower lane). (b) The morphological features of porcine GCs after 150 μM H2O2 treatment were observed and recorded by a stereomicroscope. (c) Cell viability was measured in control (PBS) and oxidative stress groups (H2O2) after treatment for 2 h. (d) The apoptosis rate of porcine GCs treated with H2O2 was detected by flow cytometry analysis. Data are represented as mean ± S.E.M. ∗∗P < 0.01 with two-tailed Student's t-test. Figure 2S: functional annotation of DEmiRNAs in porcine GCs under oxidative stress, related to Figure 2. (a) The heat map depicting the Gene Ontology (GO) enrichment analyses of DEmiRNAs in porcine GCs treated with 150 μM H2O2. Clustering analyses were performed at both DEmiRNAs and GO term levels. (b) DEmiRNA/KEGG pathway clustering was analyzed in porcine GCs undergoing oxidative stress. 11 significant enrichment signaling pathways existed in the heat map. Figure 3S: hub gene expression validation, related to Figure 3. The expression levels of 6 hub genes were verified by qRT-PCR. Black columns indicate data from RNA-seq, and red columns indicate qRT-PCR data. Supplementary Table S1: primers used for qRT-PCR in this study. Supplementary Table S2: differentially expressed mRNAs in pGCs treated with H2O2. Supplementary Table S3: differentially expressed miRNAs in pGCs treated with H2O2. Supplementary Table S4: GO enrichment analysis of DEmRNAs after H2O2 treatment. Supplementary Table S5: KEGG pathway analysis of DEmRNAs after H2O2 treatment. Supplementary Table S6: GO enrichment analysis of DEmiRNAs after H2O2 treatment. Supplementary Table S7: KEGG pathway analysis of DEmiRNAs after H2O2 treatment. Supplementary Table S8: hub genes in the protein-protein interaction network. Supplementary Table S9: hub genes and miRNAs in the miRNA-mRNA interaction n [file 1041583.f1.docx]

**Integrated analysis of miRNA-mRNA interaction network in porcine granulosa cells undergoing oxidative stress**

Xing Du, Qiqi Li, Qiuyu Cao, Siqi Wang, Honglin Liu, Qifa Li

*College of Animal Science and Technology, Nanjing Agricultural University, Nanjing, 210095, China*

Correspondence should be addressed to Qifa Li; [liqifa@njau.edu.cn](mailto:(liqifa@njau.edu.cn)。)

**Supplementary Materials**

**Figure 1S**


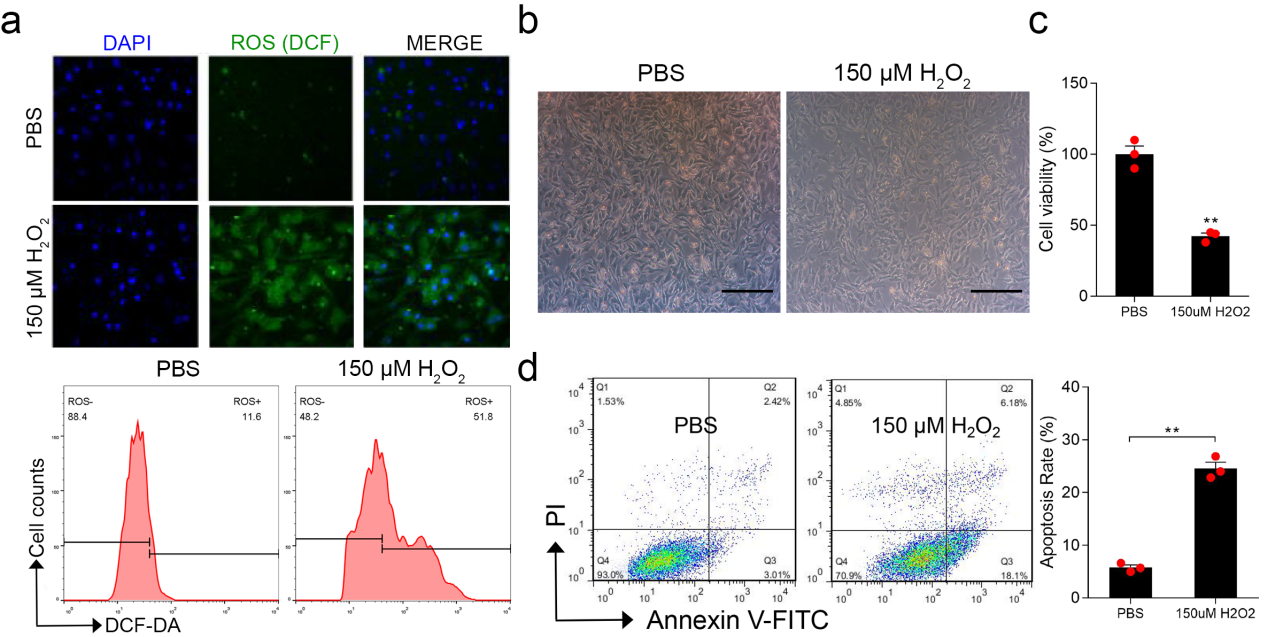


**Figure 1S. Oxidative stress impaired porcine granulosa cells.** (a) ROS levels in porcine GCs treated with 150 μM H_2_O_2_ were detected by fluorescence microscopy (upper lane) and flow cytometry analysis (lower lane). (b) The morphological features of porcine GCs after 150 μM H_2_O_2_ treatment were observed and recorded by a stereomicroscope. (c) Cell viability was measured in control (PBS) and oxidative stress groups (H_2_O_2_) after treatment for 2 h. (d) The apoptosis rate of porcine GCs treated with H_2_O_2_ was detected by flow cytometry analysis. Data are represented as mean ± S.E.M. ** *P* < 0.01 with two-tailed Student’s *t*-test.

**Figure 2S**


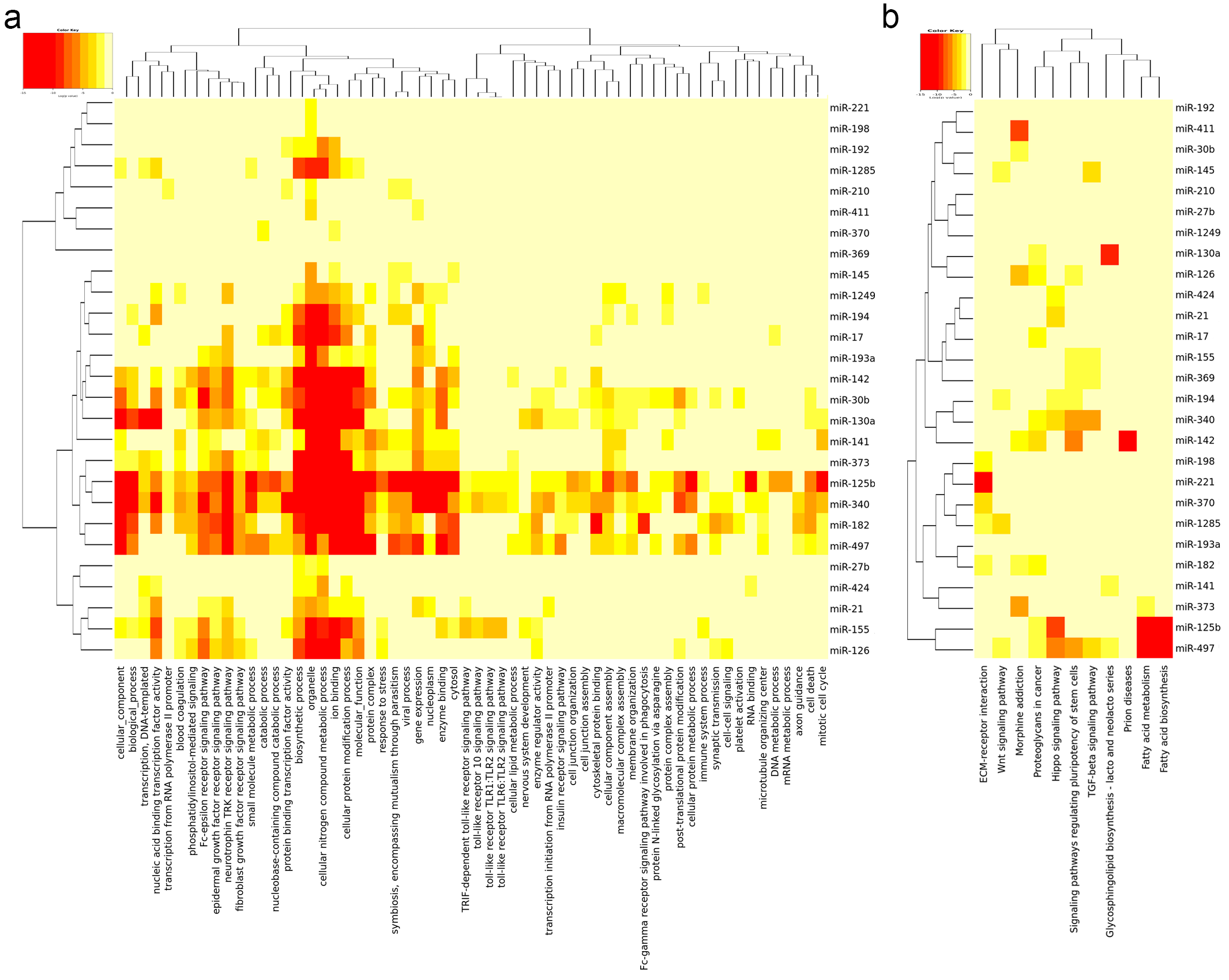


**Figure 2S. Functional annotation of DEmiRNAs in porcine GCs under oxidative stress, related to Figure 2.** (a) The heatmap depicting the Gene Ontology (GO) enrichment analyses of DEmiRNAs in porcine GCs treated with 150 μM H_2_O_2_. Clustering analyses were performed at both DEmiRNAs and GO terms levels. (b) DEmiRNAs/KEGG pathways clustering was analyzed in porcine GCs undergoing oxidative stress. 11 significant enrichment signaling pathways were existed in the heatmap.

**Figure 3S**

**
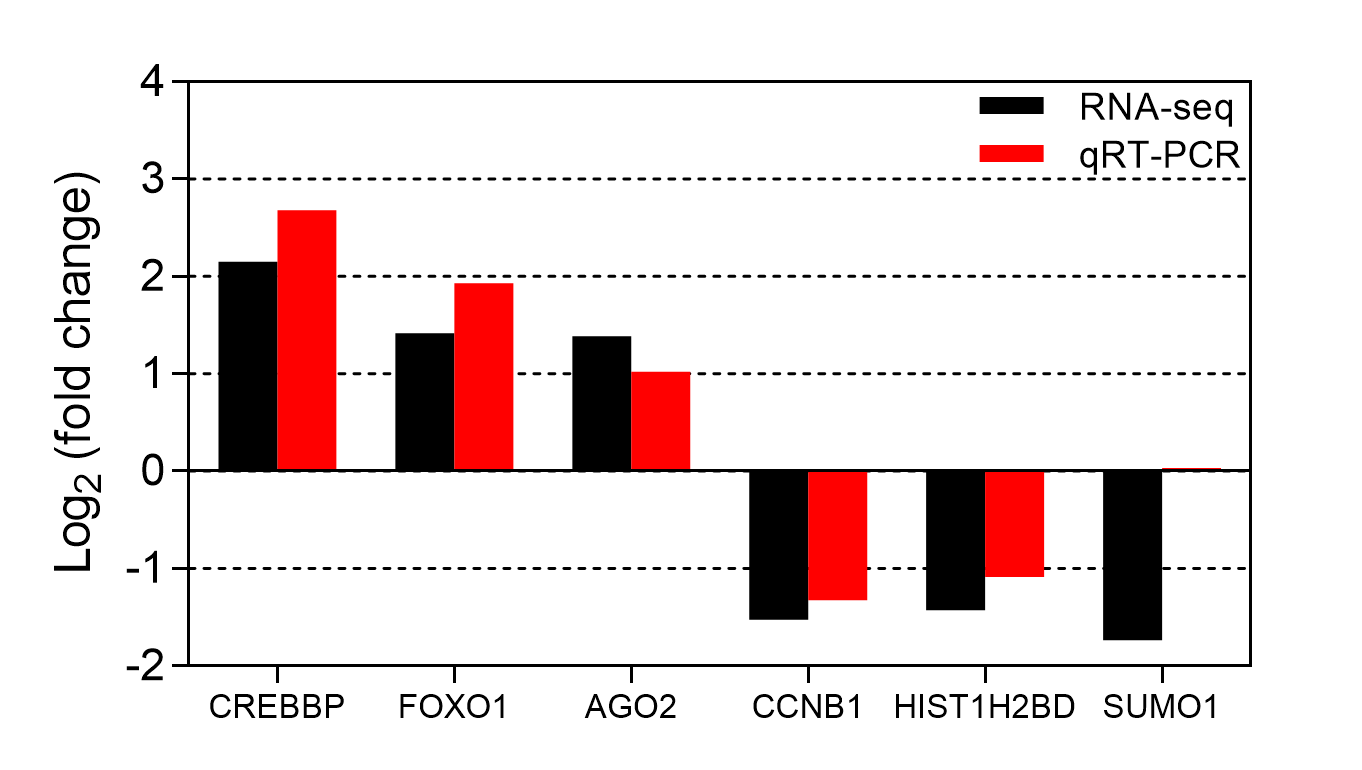
**

**Figure 3S. Hub gene expression validation, related to Figure 3.** The expression levels of 6 hub genes were verified by qRT-PCR. Black columns indicate data from RNA-seq and red columns indicate qRT-PCR data.

**Supplementary Table S1. Primers used for qRT-PCR in this study.**

| **Gene** | **Primers (5’-3’)** | **Tm (℃)** | **Product length (bp)** |
| --- | --- | --- | --- |
| *SYVN1* | F:TGGCTGAGGACCGTGTGGAC | 60.0 | 128 |
|  | R:GTGATAGGCATGGCTGACGAAGAG |  |  |
| *MAF1* | F:ACGGCAGCCTCTGGTCCTTC | 58.5 | 162 |
|  | R:CCTCCTCCTCCTCCTCCTCCTC |  |  |
| *SAC3* | F:TGTGCCAGGCTCACGGACTG | 56.0 | 160 |
|  | R:TCCTCTGCCATGACCACCTCTTC |  |  |
| *EDN1* | F:CTTCTGCCACCTGGACATCATCTG | 60.0 | 149 |
|  | R:GGCTGGCACACTGGCATCTATC |  |  |
| *SOD3* | F:GCTGCTCTGTGCTTACCTGCTC | 60.0 | 137 |
|  | R:ACTCCTGCCAGATCTCCGTCAC |  |  |
| *COX-3* | F:TCCGAGGTTCTGTTCTTCACTG | 56.0 | 150 |
|  | R:GAGGAGGATTGAGGTGTTTAGTAGG |  |  |
| *CEBPD* | F:CAACAGCAACCATAAGGCTGG | 62.0 | 281 |
|  | R:GCTTGCCCGCTGCTTTGT |  |  |
| *FBXW9* | F:ACTTCTGCCTGGCTGATGGG | 63.0 | 311 |
|  | R:TGCTGCCTTGCCCTTTATCTC |  |  |
| *GJA1* | F:TTTCCGAATCCTGCTCTTGG | 61.0 | 244 |
|  | R:TTCCTCCTCTTTCTTGTTCAGTTTC |  |  |
| *SERPINB2* | F:GGCCCTTATCCTGATGCTATTT | 60.0 | 328 |
|  | R:CCATCTTGGTCTCCGCATCTAC |  |  |
| *CREBBP* | F:GTCCTGTTTGCCTCCCTTTG | 60.0 | 258 |
|  | R:GAGGCTGTGCTGGTTGCTG |  |  |
| *FOXO1* | F:AGGGAGGCAAGAGTGGGAAAT | 58.0 | 219 |
|  | R:GCCGAAATGTACTCCAGTTATCAA |  |  |
| *AGO2* | F:CCATCGGGAGGGATAAGGT | 62.0 | 306 |
|  | R:AAGGCCGCACCGACTGAT |  |  |
| *SUMO1* | F:CCTTCAACTGAGGACTTGGGTG | 61.0 | 156 |
|  | R:CATTGGAACGCCCTGTCTTT |  |  |
| *CCNB1* | F:TTGACTGGCTAGTGCAGGTTC | 60.0 | 177 |
|  | R:CTGGAGGGTACATTTCTTCATA |  |  |
| *HIST1H2BD* | F:AGGATGGCAAGAAGCGAAAG | 56.0 | 107 |
|  | R:CATGGCTTTGGACGAGATGC |  |  |

**Supplementary Table S2. Differentially expressed mRNAs in pGCs treated with H_2_O_2_.**

| **Ensembl ID** | **FDR** | **log2FC** | **Regulation** | **Ensembl ID** | **FDR** | **log2FC** | **Regulation** |
| --- | --- | --- | --- | --- | --- | --- | --- |
| ENSSSCG00000018082 | 1.43E-53 | -9.445 | down | ENSSSCG00000012319 | 1.19E-50 | -9.127 | down |
| ENSSSCG00000032367 | 7.22E-29 | -7.205 | down | ENSSSCG00000036620 | 5.99E-27 | -6.979 | down |
| ENSSSCG00000036469 | 5.53E-26 | -6.642 | down | Sus_newGene_126488 | 8.28E-25 | -6.512 | down |
| ENSSSCG00000028892 | 2.38E-23 | -6.337 | down | Sus_newGene_34816 | 1.33E-21 | -6.214 | down |
| ENSSSCG00000013725 | 8.09E-21 | -6.045 | down | ENSSSCG00000040167 | 1.09E-21 | -5.935 | down |
| ENSSSCG00000010068 | 0.00E+00 | -5.734 | down | ENSSSCG00000002906 | 0.00E+00 | -5.731 | down |
| ENSSSCG00000007745 | 0.00E+00 | -5.718 | down | ENSSSCG00000034719 | 0.00E+00 | -5.691 | down |
| ENSSSCG00000038542 | 0.00E+00 | -5.494 | down | ENSSSCG00000011403 | 0.00E+00 | -5.427 | down |
| ENSSSCG00000021811 | 0.00E+00 | -5.295 | down | Sus_newGene_132202 | 0.00E+00 | -5.221 | down |
| Sus_newGene_189665 | 0.00E+00 | -5.086 | down | ENSSSCG00000013269 | 0.00E+00 | -4.981 | down |
| Sus_newGene_80075 | 3.33E-16 | -4.958 | down | Sus_newGene_140785 | 4.88E-15 | -4.830 | down |
| Sus_newGene_1477 | 2.21E-14 | -4.761 | down | ENSSSCG00000018026 | 6.77E-15 | -4.578 | down |
| ENSSSCG00000029291 | 0.00E+00 | -4.548 | down | Sus_newGene_144291 | 1.90E-12 | -4.545 | down |
| ENSSSCG00000031119 | 0.00E+00 | -4.542 | down | Sus_newGene_147132 | 3.30E-12 | -4.517 | down |
| ENSSSCG00000037584 | 1.34E-14 | -4.364 | down | Sus_newGene_46975 | 1.84E-10 | -4.308 | down |
| Sus_newGene_31052 | 5.26E-14 | -4.301 | down | Sus_newGene_22524 | 0.00E+00 | -4.136 | down |
| ENSSSCG00000037257 | 5.51E-09 | -4.119 | down | ENSSSCG00000012530 | 1.05E-08 | -4.081 | down |
| Sus_newGene_75062 | 1.05E-08 | -4.081 | down | Sus_newGene_132294 | 1.45E-08 | -4.062 | down |
| ENSSSCG00000004612 | 0.00E+00 | -3.630 | down | Sus_newGene_146134 | 0.00E+00 | -3.588 | down |
| ENSSSCG00000038945 | 0.00E+00 | -3.576 | down | ENSSSCG00000039416 | 0.00E+00 | -3.561 | down |
| ENSSSCG00000033148 | 0.00E+00 | -3.546 | down | ENSSSCG00000039587 | 0.00E+00 | -3.540 | down |
| ENSSSCG00000036675 | 3.11E-14 | -3.492 | down | ENSSSCG00000013643 | 0.00E+00 | -3.437 | down |
| ENSSSCG00000040638 | 0.00E+00 | -3.411 | down | ENSSSCG00000023998 | 0.00E+00 | -3.386 | down |
| ENSSSCG00000004890 | 0.00E+00 | -3.371 | down | ENSSSCG00000004241 | 0.00E+00 | -3.343 | down |
| ENSSSCG00000032857 | 5.44E-15 | -3.329 | down | Sus_newGene_146114 | 2.73E-13 | -3.314 | down |
| ENSSSCG00000007198 | 1.35E-06 | -3.243 | down | ENSSSCG00000024363 | 0.00E+00 | -3.093 | down |
| ENSSSCG00000034755 | 1.53E-11 | -3.060 | down | ENSSSCG00000001469 | 8.51E-07 | -3.056 | down |
| ENSSSCG00000001546 | 0.00E+00 | -3.034 | down | ENSSSCG00000031547 | 4.11E-15 | -2.990 | down |
| ENSSSCG00000015595 | 0.00E+00 | -2.951 | down | ENSSSCG00000014310 | 4.20E-07 | -2.939 | down |
| Sus_newGene_146112 | 1.11E-15 | -2.903 | down | ENSSSCG00000006530 | 3.20E-09 | -2.899 | down |
| ENSSSCG00000006073 | 1.05E-05 | -2.895 | down | ENSSSCG00000025117 | 0.00E+00 | -2.767 | down |
| ENSSSCG00000028802 | 3.07E-06 | -2.751 | down | Sus_newGene_146673 | 3.07E-06 | -2.751 | down |
| Sus_newGene_118277 | 6.98E-07 | -2.731 | down | ENSSSCG00000038712 | 1.22E-12 | -2.724 | down |
| Sus_newGene_22521 | 1.67E-10 | -2.699 | down | ENSSSCG00000030388 | 1.39E-05 | -2.654 | down |
| ENSSSCG00000003926 | 9.60E-10 | -2.630 | down | Sus_newGene_141241 | 3.33E-15 | -2.595 | down |
| Sus_newGene_144966 | 3.12E-08 | -2.568 | down | Sus_newGene_21346 | 2.30E-04 | -2.526 | down |
| ENSSSCG00000011685 | 4.27E-09 | -2.523 | down | ENSSSCG00000003481 | 9.27E-13 | -2.518 | down |
| ENSSSCG00000006690 | 7.36E-07 | -2.496 | down | ENSSSCG00000015792 | 1.62E-07 | -2.434 | down |
| ENSSSCG00000010134 | 1.75E-08 | -2.422 | down | ENSSSCG00000011450 | 1.96E-04 | -2.417 | down |
| ENSSSCG00000024439 | 1.96E-04 | -2.417 | down | ENSSSCG00000007564 | 5.39E-04 | -2.400 | down |
| ENSSSCG00000040843 | 2.52E-04 | -2.398 | down | Sus_newGene_3622 | 8.49E-11 | -2.393 | down |
| Sus_newGene_1759 | 8.26E-06 | -2.380 | down | Sus_newGene_204565 | 1.59E-04 | -2.380 | down |
| Sus_newGene_141006 | 2.45E-09 | -2.370 | down | ENSSSCG00000004167 | 1.52E-10 | -2.355 | down |
| ENSSSCG00000038439 | 2.47E-07 | -2.355 | down | ENSSSCG00000036048 | 1.24E-05 | -2.354 | down |
| ENSSSCG00000001392 | 3.53E-06 | -2.347 | down | ENSSSCG00000006579 | 4.00E-05 | -2.311 | down |
| ENSSSCG00000031610 | 4.11E-10 | -2.286 | down | ENSSSCG00000032609 | 6.67E-09 | -2.273 | down |
| Sus_newGene_141240 | 5.69E-12 | -2.267 | down | Sus_newGene_9107 | 7.64E-07 | -2.258 | down |
| ENSSSCG00000028186 | 9.45E-12 | -2.250 | down | ENSSSCG00000033997 | 1.76E-04 | -2.246 | down |
| ENSSSCG00000030632 | 5.99E-12 | -2.244 | down | ENSSSCG00000037890 | 8.93E-12 | -2.237 | down |
| Sus_newGene_141899 | 2.02E-07 | -2.230 | down | Sus_newGene_144468 | 2.53E-11 | -2.207 | down |
| Sus_newGene_141897 | 8.17E-05 | -2.200 | down | Sus_newGene_141008 | 1.02E-06 | -2.199 | down |
| Sus_newGene_143833 | 8.69E-04 | -2.169 | down | Sus_newGene_133114 | 5.90E-05 | -2.168 | down |
| ENSSSCG00000006648 | 3.83E-10 | -2.163 | down | ENSSSCG00000006001 | 7.75E-08 | -2.159 | down |
| ENSSSCG00000012669 | 4.57E-06 | -2.153 | down | ENSSSCG00000004554 | 9.67E-09 | -2.149 | down |
| ENSSSCG00000028274 | 2.56E-05 | -2.095 | down | ENSSSCG00000001702 | 2.29E-10 | -2.089 | down |
| Sus_newGene_22525 | 7.71E-05 | -2.081 | down | ENSSSCG00000014385 | 4.91E-03 | -2.076 | down |
| ENSSSCG00000032503 | 4.91E-03 | -2.076 | down | ENSSSCG00000022413 | 9.16E-09 | -2.071 | down |
| ENSSSCG00000008959 | 1.10E-04 | -2.057 | down | Sus_newGene_8516 | 3.32E-04 | -2.025 | down |
| ENSSSCG00000010522 | 1.53E-03 | -2.021 | down | ENSSSCG00000040119 | 7.87E-08 | -2.016 | down |
| ENSSSCG00000001696 | 1.26E-09 | -2.010 | down | Sus_newGene_145875 | 8.32E-09 | -2.005 | down |
| ENSSSCG00000036946 | 4.95E-09 | -1.994 | down | ENSSSCG00000021383 | 3.89E-03 | -1.980 | down |
| Sus_newGene_56362 | 2.23E-04 | -1.969 | down | ENSSSCG00000031174 | 3.20E-04 | -1.963 | down |
| ENSSSCG00000010829 | 5.69E-09 | -1.955 | down | ENSSSCG00000011465 | 6.21E-08 | -1.947 | down |
| ENSSSCG00000032170 | 8.43E-09 | -1.937 | down | ENSSSCG00000009216 | 5.74E-08 | -1.933 | down |
| ENSSSCG00000010444 | 6.30E-04 | -1.896 | down | ENSSSCG00000017195 | 1.19E-02 | -1.889 | down |
| ENSSSCG00000040080 | 1.17E-04 | -1.881 | down | ENSSSCG00000027628 | 7.91E-08 | -1.876 | down |
| ENSSSCG00000005216 | 1.24E-05 | -1.872 | down | ENSSSCG00000005502 | 4.08E-04 | -1.861 | down |
| ENSSSCG00000021748 | 4.40E-05 | -1.857 | down | ENSSSCG00000013655 | 2.09E-04 | -1.854 | down |
| ENSSSCG00000017868 | 2.56E-03 | -1.852 | down | Sus_newGene_91668 | 4.02E-03 | -1.839 | down |
| ENSSSCG00000011322 | 6.21E-07 | -1.833 | down | Sus_newGene_131715 | 1.50E-03 | -1.832 | down |
| ENSSSCG00000035649 | 2.31E-02 | -1.831 | down | ENSSSCG00000022773 | 2.89E-03 | -1.821 | down |
| Sus_newGene_162103 | 7.73E-08 | -1.820 | down | Sus_newGene_144968 | 2.40E-05 | -1.819 | down |
| ENSSSCG00000037358 | 1.24E-05 | -1.810 | down | ENSSSCG00000034639 | 2.13E-03 | -1.806 | down |
| ENSSSCG00000006163 | 9.39E-08 | -1.802 | down | ENSSSCG00000036562 | 3.05E-07 | -1.799 | down |
| ENSSSCG00000015627 | 3.58E-02 | -1.792 | down | ENSSSCG00000021130 | 5.20E-06 | -1.786 | down |
| Sus_newGene_126976 | 3.02E-03 | -1.779 | down | ENSSSCG00000008873 | 1.80E-07 | -1.775 | down |
| ENSSSCG00000008678 | 1.76E-07 | -1.774 | down | ENSSSCG00000039360 | 1.99E-07 | -1.773 | down |
| Sus_newGene_144996 | 1.80E-03 | -1.769 | down | ENSSSCG00000010059 | 2.05E-07 | -1.767 | down |
| ENSSSCG00000031122 | 5.72E-07 | -1.753 | down | ENSSSCG00000017643 | 5.54E-03 | -1.750 | down |
| Sus_newGene_80109 | 4.06E-07 | -1.750 | down | ENSSSCG00000038965 | 1.32E-03 | -1.749 | down |
| ENSSSCG00000038554 | 2.98E-07 | -1.748 | down | ENSSSCG00000005995 | 1.64E-04 | -1.748 | down |
| Sus_newGene_141287 | 1.86E-04 | -1.747 | down | ENSSSCG00000001431 | 7.51E-05 | -1.737 | down |
| ENSSSCG00000035842 | 3.92E-07 | -1.735 | down | ENSSSCG00000015044 | 4.64E-07 | -1.735 | down |
| ENSSSCG00000003369 | 4.56E-07 | -1.726 | down | ENSSSCG00000025353 | 2.12E-02 | -1.725 | down |
| ENSSSCG00000024463 | 4.54E-07 | -1.724 | down | ENSSSCG00000033006 | 2.65E-04 | -1.723 | down |
| ENSSSCG00000033843 | 5.84E-06 | -1.720 | down | ENSSSCG00000015197 | 2.10E-03 | -1.714 | down |
| ENSSSCG00000034743 | 2.27E-04 | -1.712 | down | ENSSSCG00000021798 | 5.65E-07 | -1.711 | down |
| ENSSSCG00000014556 | 6.05E-07 | -1.708 | down | ENSSSCG00000037815 | 8.13E-07 | -1.707 | down |
| ENSSSCG00000038615 | 9.03E-07 | -1.704 | down | ENSSSCG00000033516 | 1.10E-06 | -1.698 | down |
| ENSSSCG00000000680 | 8.64E-03 | -1.696 | down | ENSSSCG00000020675 | 9.22E-06 | -1.692 | down |
| ENSSSCG00000006828 | 1.11E-04 | -1.687 | down | ENSSSCG00000007559 | 3.81E-06 | -1.685 | down |
| ENSSSCG00000017311 | 2.20E-06 | -1.685 | down | ENSSSCG00000036975 | 5.74E-06 | -1.679 | down |
| ENSSSCG00000009821 | 1.67E-06 | -1.672 | down | ENSSSCG00000037929 | 3.68E-05 | -1.657 | down |
| ENSSSCG00000022029 | 2.17E-02 | -1.656 | down | ENSSSCG00000039881 | 1.93E-06 | -1.652 | down |
| ENSSSCG00000026330 | 1.46E-02 | -1.652 | down | ENSSSCG00000030577 | 2.05E-06 | -1.648 | down |
| ENSSSCG00000006846 | 1.94E-02 | -1.646 | down | Sus_newGene_163532 | 4.36E-06 | -1.646 | down |
| Sus_newGene_15954 | 1.29E-04 | -1.644 | down | ENSSSCG00000013011 | 4.03E-02 | -1.643 | down |
| ENSSSCG00000013235 | 2.35E-05 | -1.629 | down | ENSSSCG00000017022 | 8.92E-06 | -1.629 | down |
| ENSSSCG00000003561 | 6.34E-06 | -1.624 | down | ENSSSCG00000016031 | 2.06E-02 | -1.622 | down |
| ENSSSCG00000028804 | 3.26E-03 | -1.622 | down | ENSSSCG00000001463 | 5.29E-03 | -1.618 | down |
| ENSSSCG00000031724 | 4.67E-05 | -1.607 | down | Sus_newGene_202959 | 1.67E-02 | -1.607 | down |
| ENSSSCG00000010600 | 1.74E-04 | -1.604 | down | ENSSSCG00000008664 | 9.33E-04 | -1.601 | down |
| ENSSSCG00000013497 | 1.51E-02 | -1.599 | down | ENSSSCG00000033790 | 3.79E-03 | -1.590 | down |
| ENSSSCG00000037241 | 3.57E-05 | -1.586 | down | ENSSSCG00000006590 | 6.12E-03 | -1.583 | down |
| ENSSSCG00000028192 | 1.60E-02 | -1.579 | down | ENSSSCG00000013005 | 8.29E-06 | -1.574 | down |
| ENSSSCG00000001506 | 2.44E-05 | -1.562 | down | ENSSSCG00000006862 | 2.56E-05 | -1.561 | down |
| ENSSSCG00000039488 | 1.11E-02 | -1.558 | down | ENSSSCG00000040743 | 2.16E-05 | -1.555 | down |
| ENSSSCG00000026248 | 1.64E-05 | -1.555 | down | Sus_newGene_106261 | 1.82E-03 | -1.552 | down |
| ENSSSCG00000015037 | 1.21E-04 | -1.550 | down | ENSSSCG00000017584 | 1.42E-05 | -1.548 | down |
| ENSSSCG00000007161 | 1.44E-05 | -1.545 | down | ENSSSCG00000011234 | 2.70E-04 | -1.544 | down |
| Sus_newGene_58218 | 6.06E-03 | -1.544 | down | ENSSSCG00000006372 | 1.25E-03 | -1.540 | down |
| ENSSSCG00000009100 | 4.08E-04 | -1.538 | down | ENSSSCG00000040681 | 1.42E-05 | -1.537 | down |
| ENSSSCG00000035473 | 2.96E-05 | -1.528 | down | ENSSSCG00000029326 | 6.48E-05 | -1.525 | down |
| ENSSSCG00000025965 | 1.43E-03 | -1.521 | down | ENSSSCG00000036063 | 2.99E-05 | -1.517 | down |
| ENSSSCG00000004518 | 2.71E-03 | -1.516 | down | ENSSSCG00000010947 | 1.43E-02 | -1.514 | down |
| ENSSSCG00000026585 | 1.01E-03 | -1.512 | down | ENSSSCG00000016255 | 5.85E-05 | -1.511 | down |
| ENSSSCG00000031531 | 4.67E-05 | -1.509 | down | ENSSSCG00000029163 | 3.41E-05 | -1.501 | down |
| ENSSSCG00000035176 | 6.96E-04 | -1.498 | down | ENSSSCG00000000002 | 4.11E-02 | -1.496 | down |
| ENSSSCG00000034527 | 4.11E-05 | -1.491 | down | ENSSSCG00000004596 | 2.40E-02 | -1.480 | down |
| ENSSSCG00000032431 | 2.85E-03 | -1.470 | down | ENSSSCG00000010732 | 4.85E-05 | -1.467 | down |
| ENSSSCG00000032123 | 9.17E-04 | -1.466 | down | ENSSSCG00000021557 | 4.97E-05 | -1.465 | down |
| ENSSSCG00000021834 | 6.57E-03 | -1.460 | down | ENSSSCG00000040272 | 1.81E-03 | -1.455 | down |
| ENSSSCG00000026395 | 1.59E-02 | -1.455 | down | ENSSSCG00000031727 | 4.56E-02 | -1.446 | down |
| ENSSSCG00000009877 | 7.72E-03 | -1.441 | down | ENSSSCG00000030300 | 8.14E-04 | -1.439 | down |
| Sus_newGene_131056 | 2.47E-02 | -1.436 | down | Sus_newGene_67448 | 1.15E-03 | -1.435 | down |
| ENSSSCG00000035452 | 9.22E-05 | -1.433 | down | ENSSSCG00000021411 | 1.05E-04 | -1.429 | down |
| ENSSSCG00000034136 | 2.40E-04 | -1.427 | down | ENSSSCG00000000657 | 1.26E-04 | -1.425 | down |
| ENSSSCG00000034986 | 2.99E-03 | -1.425 | down | ENSSSCG00000003697 | 2.77E-03 | -1.424 | down |
| ENSSSCG00000002633 | 1.86E-04 | -1.422 | down | ENSSSCG00000032769 | 1.09E-04 | -1.418 | down |
| ENSSSCG00000012773 | 1.33E-02 | -1.416 | down | ENSSSCG00000016414 | 3.88E-03 | -1.413 | down |
| ENSSSCG00000012508 | 6.62E-04 | -1.407 | down | ENSSSCG00000003201 | 4.80E-04 | -1.406 | down |
| Sus_newGene_176555 | 3.34E-04 | -1.406 | down | Sus_newGene_144999 | 1.07E-03 | -1.400 | down |
| ENSSSCG00000028606 | 1.61E-04 | -1.395 | down | ENSSSCG00000009349 | 4.13E-04 | -1.394 | down |
| ENSSSCG00000000185 | 1.79E-04 | -1.392 | down | ENSSSCG00000036311 | 2.27E-03 | -1.384 | down |
| ENSSSCG00000003453 | 2.20E-04 | -1.382 | down | ENSSSCG00000037174 | 3.42E-04 | -1.379 | down |
| ENSSSCG00000009676 | 2.11E-04 | -1.378 | down | ENSSSCG00000007560 | 1.94E-02 | -1.378 | down |
| ENSSSCG00000025588 | 3.82E-04 | -1.371 | down | ENSSSCG00000004601 | 2.28E-03 | -1.369 | down |
| ENSSSCG00000033180 | 2.65E-04 | -1.363 | down | ENSSSCG00000026055 | 8.63E-04 | -1.359 | down |
| ENSSSCG00000029507 | 2.86E-04 | -1.359 | down | ENSSSCG00000012768 | 8.80E-03 | -1.356 | down |
| ENSSSCG00000013114 | 2.63E-03 | -1.355 | down | ENSSSCG00000004534 | 2.63E-03 | -1.355 | down |
| ENSSSCG00000009378 | 8.37E-04 | -1.355 | down | ENSSSCG00000005056 | 8.50E-04 | -1.345 | down |
| ENSSSCG00000006140 | 1.78E-02 | -1.344 | down | ENSSSCG00000034072 | 2.77E-03 | -1.342 | down |
| Sus_newGene_141284 | 3.10E-03 | -1.341 | down | ENSSSCG00000016823 | 4.94E-03 | -1.339 | down |
| ENSSSCG00000011601 | 4.47E-04 | -1.339 | down | ENSSSCG00000038313 | 4.91E-04 | -1.336 | down |
| ENSSSCG00000035610 | 1.10E-03 | -1.334 | down | ENSSSCG00000007307 | 2.66E-02 | -1.331 | down |
| ENSSSCG00000020906 | 3.75E-03 | -1.330 | down | ENSSSCG00000015924 | 4.03E-02 | -1.326 | down |
| ENSSSCG00000014581 | 6.09E-04 | -1.325 | down | ENSSSCG00000024644 | 6.60E-04 | -1.323 | down |
| ENSSSCG00000040317 | 2.06E-03 | -1.323 | down | ENSSSCG00000039847 | 5.54E-04 | -1.318 | down |
| ENSSSCG00000037791 | 7.55E-04 | -1.317 | down | ENSSSCG00000030182 | 2.28E-03 | -1.315 | down |
| Sus_newGene_166153 | 1.01E-02 | -1.314 | down | Sus_newGene_146669 | 1.43E-02 | -1.313 | down |
| Sus_newGene_132451 | 2.56E-02 | -1.302 | down | ENSSSCG00000030681 | 8.24E-04 | -1.301 | down |
| ENSSSCG00000040486 | 1.27E-03 | -1.298 | down | ENSSSCG00000009361 | 9.45E-04 | -1.297 | down |
| ENSSSCG00000038524 | 3.45E-02 | -1.297 | down | ENSSSCG00000005206 | 3.19E-03 | -1.295 | down |
| ENSSSCG00000031526 | 1.16E-03 | -1.294 | down | ENSSSCG00000017159 | 8.03E-04 | -1.294 | down |
| ENSSSCG00000022322 | 1.06E-03 | -1.294 | down | ENSSSCG00000037022 | 6.85E-03 | -1.292 | down |
| ENSSSCG00000035719 | 8.76E-04 | -1.291 | down | ENSSSCG00000040047 | 9.73E-04 | -1.291 | down |
| ENSSSCG00000009519 | 8.44E-03 | -1.290 | down | ENSSSCG00000009746 | 1.72E-03 | -1.290 | down |
| ENSSSCG00000005761 | 9.33E-04 | -1.285 | down | ENSSSCG00000010471 | 1.62E-03 | -1.284 | down |
| ENSSSCG00000002849 | 6.26E-03 | -1.284 | down | ENSSSCG00000006333 | 2.07E-03 | -1.284 | down |
| Sus_newGene_146099 | 5.64E-03 | -1.284 | down | ENSSSCG00000034260 | 1.27E-02 | -1.280 | down |
| ENSSSCG00000025260 | 2.96E-02 | -1.280 | down | ENSSSCG00000027226 | 3.91E-02 | -1.280 | down |
| Sus_newGene_139994 | 3.83E-03 | -1.278 | down | ENSSSCG00000031715 | 1.64E-03 | -1.273 | down |
| ENSSSCG00000027956 | 1.69E-02 | -1.268 | down | ENSSSCG00000036274 | 1.63E-03 | -1.267 | down |
| ENSSSCG00000022635 | 3.03E-03 | -1.265 | down | ENSSSCG00000031736 | 2.19E-03 | -1.265 | down |
| ENSSSCG00000012171 | 2.10E-03 | -1.264 | down | ENSSSCG00000014672 | 1.65E-02 | -1.264 | down |
| ENSSSCG00000013534 | 1.44E-03 | -1.262 | down | ENSSSCG00000006474 | 3.01E-03 | -1.259 | down |
| ENSSSCG00000000635 | 1.64E-03 | -1.256 | down | ENSSSCG00000038549 | 3.06E-03 | -1.255 | down |
| ENSSSCG00000034014 | 1.46E-03 | -1.253 | down | ENSSSCG00000011576 | 1.93E-03 | -1.252 | down |
| ENSSSCG00000008132 | 1.88E-03 | -1.252 | down | ENSSSCG00000005838 | 1.61E-03 | -1.251 | down |
| Sus_newGene_94064 | 1.50E-03 | -1.251 | down | ENSSSCG00000005478 | 1.55E-03 | -1.249 | down |
| ENSSSCG00000008930 | 1.99E-03 | -1.249 | down | ENSSSCG00000036114 | 2.64E-03 | -1.248 | down |
| ENSSSCG00000000271 | 1.36E-02 | -1.247 | down | ENSSSCG00000024776 | 1.95E-03 | -1.244 | down |
| ENSSSCG00000018092 | 2.09E-03 | -1.243 | down | ENSSSCG00000023665 | 1.87E-03 | -1.238 | down |
| ENSSSCG00000012744 | 1.85E-03 | -1.236 | down | ENSSSCG00000026064 | 2.53E-03 | -1.235 | down |
| ENSSSCG00000008572 | 1.64E-02 | -1.234 | down | ENSSSCG00000022128 | 2.00E-03 | -1.232 | down |
| ENSSSCG00000012519 | 2.35E-03 | -1.225 | down | ENSSSCG00000037307 | 2.38E-03 | -1.224 | down |
| Sus_newGene_67447 | 4.98E-02 | -1.219 | down | ENSSSCG00000003876 | 2.25E-02 | -1.219 | down |
| ENSSSCG00000032496 | 1.60E-02 | -1.214 | down | ENSSSCG00000011740 | 2.64E-02 | -1.213 | down |
| ENSSSCG00000013593 | 1.37E-02 | -1.210 | down | ENSSSCG00000040751 | 2.71E-03 | -1.210 | down |
| ENSSSCG00000014319 | 3.77E-03 | -1.210 | down | ENSSSCG00000025187 | 3.01E-03 | -1.208 | down |
| ENSSSCG00000016679 | 1.05E-02 | -1.207 | down | ENSSSCG00000004762 | 3.98E-03 | -1.206 | down |
| ENSSSCG00000028695 | 1.33E-02 | -1.203 | down | ENSSSCG00000028066 | 3.99E-03 | -1.201 | down |
| ENSSSCG00000006051 | 3.23E-03 | -1.200 | down | ENSSSCG00000005688 | 3.33E-03 | -1.196 | down |
| ENSSSCG00000032344 | 6.46E-03 | -1.195 | down | ENSSSCG00000000853 | 4.75E-02 | -1.193 | down |
| ENSSSCG00000038941 | 1.73E-02 | -1.191 | down | ENSSSCG00000003147 | 2.32E-02 | -1.190 | down |
| ENSSSCG00000017042 | 4.10E-03 | -1.189 | down | ENSSSCG00000035304 | 3.96E-03 | -1.189 | down |
| ENSSSCG00000021706 | 9.55E-03 | -1.187 | down | ENSSSCG00000009466 | 3.84E-03 | -1.186 | down |
| ENSSSCG00000040961 | 4.07E-03 | -1.184 | down | ENSSSCG00000009146 | 1.11E-02 | -1.182 | down |
| ENSSSCG00000039506 | 6.97E-03 | -1.178 | down | ENSSSCG00000003980 | 9.95E-03 | -1.176 | down |
| ENSSSCG00000017032 | 3.88E-02 | -1.176 | down | ENSSSCG00000004643 | 1.67E-02 | -1.173 | down |
| ENSSSCG00000021180 | 5.00E-03 | -1.171 | down | ENSSSCG00000033107 | 5.01E-03 | -1.169 | down |
| ENSSSCG00000015662 | 4.92E-02 | -1.165 | down | ENSSSCG00000022195 | 2.91E-02 | -1.159 | down |
| ENSSSCG00000010214 | 1.38E-02 | -1.156 | down | ENSSSCG00000031510 | 6.13E-03 | -1.155 | down |
| ENSSSCG00000011353 | 7.87E-03 | -1.154 | down | ENSSSCG00000003979 | 1.32E-02 | -1.153 | down |
| ENSSSCG00000024267 | 1.02E-02 | -1.152 | down | ENSSSCG00000012327 | 6.85E-03 | -1.151 | down |
| ENSSSCG00000011857 | 1.41E-02 | -1.151 | down | Sus_newGene_82973 | 8.74E-03 | -1.145 | down |
| ENSSSCG00000006885 | 9.68E-03 | -1.145 | down | ENSSSCG00000040622 | 7.82E-03 | -1.143 | down |
| ENSSSCG00000031782 | 7.34E-03 | -1.141 | down | ENSSSCG00000012608 | 2.54E-02 | -1.141 | down |
| ENSSSCG00000009671 | 2.85E-02 | -1.140 | down | ENSSSCG00000032165 | 9.72E-03 | -1.137 | down |
| ENSSSCG00000002863 | 8.64E-03 | -1.137 | down | ENSSSCG00000002307 | 9.16E-03 | -1.133 | down |
| ENSSSCG00000038475 | 9.93E-03 | -1.133 | down | ENSSSCG00000014274 | 7.95E-03 | -1.133 | down |
| ENSSSCG00000039915 | 1.37E-02 | -1.132 | down | ENSSSCG00000027684 | 8.37E-03 | -1.129 | down |
| ENSSSCG00000030642 | 9.19E-03 | -1.128 | down | ENSSSCG00000017420 | 8.52E-03 | -1.127 | down |
| ENSSSCG00000028414 | 1.21E-02 | -1.127 | down | ENSSSCG00000027607 | 1.07E-02 | -1.126 | down |
| ENSSSCG00000021371 | 1.11E-02 | -1.124 | down | ENSSSCG00000009670 | 3.31E-02 | -1.123 | down |
| Sus_newGene_71897 | 1.03E-02 | -1.122 | down | ENSSSCG00000012124 | 9.57E-03 | -1.119 | down |
| ENSSSCG00000012377 | 4.91E-02 | -1.115 | down | ENSSSCG00000034655 | 1.29E-02 | -1.114 | down |
| ENSSSCG00000039618 | 1.17E-02 | -1.113 | down | ENSSSCG00000030827 | 2.96E-02 | -1.112 | down |
| ENSSSCG00000014141 | 1.07E-02 | -1.111 | down | ENSSSCG00000014915 | 1.11E-02 | -1.108 | down |
| ENSSSCG00000016128 | 3.37E-02 | -1.108 | down | ENSSSCG00000027745 | 3.03E-02 | -1.108 | down |
| ENSSSCG00000004782 | 2.12E-02 | -1.107 | down | Sus_newGene_117649 | 1.14E-02 | -1.105 | down |
| ENSSSCG00000000773 | 1.19E-02 | -1.105 | down | ENSSSCG00000024299 | 1.26E-02 | -1.100 | down |
| ENSSSCG00000015545 | 2.32E-02 | -1.100 | down | ENSSSCG00000010464 | 2.01E-02 | -1.098 | down |
| ENSSSCG00000039568 | 4.23E-02 | -1.097 | down | ENSSSCG00000023747 | 1.31E-02 | -1.096 | down |
| ENSSSCG00000034617 | 4.41E-02 | -1.096 | down | ENSSSCG00000006830 | 1.50E-02 | -1.095 | down |
| ENSSSCG00000004379 | 2.54E-02 | -1.092 | down | ENSSSCG00000036820 | 1.49E-02 | -1.091 | down |
| ENSSSCG00000015301 | 1.39E-02 | -1.091 | down | ENSSSCG00000040801 | 1.89E-02 | -1.086 | down |
| ENSSSCG00000036256 | 1.60E-02 | -1.082 | down | ENSSSCG00000037953 | 1.58E-02 | -1.080 | down |
| ENSSSCG00000008336 | 2.30E-02 | -1.080 | down | ENSSSCG00000000695 | 2.50E-02 | -1.077 | down |
| ENSSSCG00000012532 | 1.78E-02 | -1.076 | down | ENSSSCG00000030655 | 2.59E-02 | -1.075 | down |
| ENSSSCG00000017492 | 1.85E-02 | -1.075 | down | ENSSSCG00000011082 | 1.75E-02 | -1.075 | down |
| ENSSSCG00000035867 | 3.73E-02 | -1.074 | down | ENSSSCG00000038220 | 1.80E-02 | -1.073 | down |
| ENSSSCG00000006175 | 2.09E-02 | -1.071 | down | ENSSSCG00000002828 | 4.51E-02 | -1.069 | down |
| Sus_newGene_152077 | 3.71E-02 | -1.069 | down | ENSSSCG00000037399 | 2.59E-02 | -1.068 | down |
| ENSSSCG00000000807 | 4.00E-02 | -1.068 | down | ENSSSCG00000024019 | 1.85E-02 | -1.067 | down |
| ENSSSCG00000021427 | 2.50E-02 | -1.061 | down | ENSSSCG00000039549 | 2.09E-02 | -1.058 | down |
| ENSSSCG00000025589 | 2.28E-02 | -1.058 | down | ENSSSCG00000016175 | 3.38E-02 | -1.057 | down |
| ENSSSCG00000015080 | 2.38E-02 | -1.057 | down | ENSSSCG00000009410 | 4.08E-02 | -1.057 | down |
| ENSSSCG00000021591 | 4.22E-02 | -1.056 | down | ENSSSCG00000011169 | 2.54E-02 | -1.055 | down |
| ENSSSCG00000014100 | 2.92E-02 | -1.055 | down | ENSSSCG00000006848 | 4.46E-02 | -1.055 | down |
| ENSSSCG00000037568 | 2.55E-02 | -1.055 | down | ENSSSCG00000012517 | 3.05E-02 | -1.055 | down |
| ENSSSCG00000026772 | 3.60E-02 | -1.055 | down | ENSSSCG00000024837 | 2.45E-02 | -1.052 | down |
| ENSSSCG00000039969 | 2.30E-02 | -1.051 | down | ENSSSCG00000031489 | 2.51E-02 | -1.049 | down |
| ENSSSCG00000035114 | 2.41E-02 | -1.048 | down | ENSSSCG00000005944 | 4.73E-02 | -1.048 | down |
| ENSSSCG00000038702 | 3.75E-02 | -1.044 | down | ENSSSCG00000002635 | 2.61E-02 | -1.042 | down |
| ENSSSCG00000014539 | 3.41E-02 | -1.041 | down | ENSSSCG00000034031 | 2.75E-02 | -1.040 | down |
| ENSSSCG00000010370 | 2.85E-02 | -1.040 | down | ENSSSCG00000035434 | 3.49E-02 | -1.037 | down |
| ENSSSCG00000011770 | 2.78E-02 | -1.037 | down | ENSSSCG00000008016 | 3.91E-02 | -1.036 | down |
| ENSSSCG00000037024 | 4.11E-02 | -1.035 | down | ENSSSCG00000013391 | 4.29E-02 | -1.035 | down |
| ENSSSCG00000004439 | 4.14E-02 | -1.034 | down | ENSSSCG00000016096 | 2.82E-02 | -1.034 | down |
| ENSSSCG00000035078 | 3.05E-02 | -1.033 | down | ENSSSCG00000038987 | 3.02E-02 | -1.029 | down |
| ENSSSCG00000035900 | 3.70E-02 | -1.028 | down | ENSSSCG00000006564 | 4.56E-02 | -1.026 | down |
| Sus_newGene_162058 | 3.38E-02 | -1.024 | down | ENSSSCG00000008659 | 4.12E-02 | -1.012 | down |
| Sus_newGene_94606 | 3.80E-02 | -1.012 | down | ENSSSCG00000029380 | 4.36E-02 | -1.008 | down |
| ENSSSCG00000032450 | 4.90E-02 | -1.005 | down | ENSSSCG00000005203 | 4.28E-02 | -1.003 | down |
| ENSSSCG00000037779 | 4.24E-02 | -1.002 | down | ENSSSCG00000004063 | 4.30E-02 | -0.998 | down |
| ENSSSCG00000032459 | 4.74E-02 | -0.991 | down | ENSSSCG00000009973 | 4.87E-02 | -0.991 | down |
| ENSSSCG00000008516 | 4.98E-02 | -0.990 | down | ENSSSCG00000017473 | 4.87E-02 | -0.986 | down |
| ENSSSCG00000005659 | 4.97E-02 | 0.971 | up | ENSSSCG00000034625 | 4.97E-02 | 0.971 | up |
| ENSSSCG00000034966 | 4.63E-02 | 0.977 | up | ENSSSCG00000026367 | 4.75E-02 | 0.975 | up |
| ENSSSCG00000000521 | 4.53E-02 | 0.980 | up | ENSSSCG00000010613 | 4.56E-02 | 0.979 | up |
| ENSSSCG00000009208 | 4.94E-02 | 0.983 | up | ENSSSCG00000018058 | 4.41E-02 | 0.982 | up |
| ENSSSCG00000015937 | 4.60E-02 | 0.984 | up | ENSSSCG00000004738 | 4.27E-02 | 0.983 | up |
| ENSSSCG00000024193 | 4.63E-02 | 0.984 | up | ENSSSCG00000001667 | 4.90E-02 | 0.984 | up |
| ENSSSCG00000017794 | 4.82E-02 | 0.985 | up | ENSSSCG00000038417 | 4.78E-02 | 0.984 | up |
| ENSSSCG00000030042 | 4.22E-02 | 0.989 | up | ENSSSCG00000005967 | 4.12E-02 | 0.988 | up |
| ENSSSCG00000026636 | 4.02E-02 | 0.990 | up | ENSSSCG00000032401 | 4.36E-02 | 0.990 | up |
| ENSSSCG00000010825 | 4.09E-02 | 0.991 | up | ENSSSCG00000003326 | 4.37E-02 | 0.990 | up |
| ENSSSCG00000012624 | 3.89E-02 | 0.992 | up | ENSSSCG00000025053 | 4.17E-02 | 0.991 | up |
| ENSSSCG00000016958 | 4.14E-02 | 0.997 | up | ENSSSCG00000006519 | 3.72E-02 | 0.997 | up |
| ENSSSCG00000013037 | 3.60E-02 | 0.998 | up | ENSSSCG00000015099 | 3.61E-02 | 0.998 | up |
| ENSSSCG00000012309 | 3.62E-02 | 0.999 | up | ENSSSCG00000010544 | 4.16E-02 | 0.998 | up |
| ENSSSCG00000014336 | 4.17E-02 | 0.999 | up | ENSSSCG00000012638 | 3.94E-02 | 0.999 | up |
| ENSSSCG00000010485 | 3.91E-02 | 1.000 | up | ENSSSCG00000011425 | 3.91E-02 | 1.000 | up |
| ENSSSCG00000004250 | 4.06E-02 | 1.001 | up | ENSSSCG00000013050 | 3.60E-02 | 1.001 | up |
| ENSSSCG00000023126 | 4.79E-02 | 1.002 | up | ENSSSCG00000016244 | 3.61E-02 | 1.002 | up |
| ENSSSCG00000034373 | 3.71E-02 | 1.003 | up | ENSSSCG00000016809 | 3.66E-02 | 1.002 | up |
| ENSSSCG00000013388 | 3.65E-02 | 1.003 | up | ENSSSCG00000030358 | 3.75E-02 | 1.003 | up |
| ENSSSCG00000005930 | 4.75E-02 | 1.004 | up | ENSSSCG00000035518 | 4.10E-02 | 1.003 | up |
| ENSSSCG00000023890 | 3.41E-02 | 1.005 | up | ENSSSCG00000037277 | 3.64E-02 | 1.004 | up |
| ENSSSCG00000005720 | 4.91E-02 | 1.007 | up | ENSSSCG00000008549 | 4.29E-02 | 1.005 | up |
| ENSSSCG00000012841 | 3.17E-02 | 1.008 | up | ENSSSCG00000015290 | 3.35E-02 | 1.007 | up |
| ENSSSCG00000032835 | 4.60E-02 | 1.009 | up | Sus_newGene_22847 | 3.31E-02 | 1.009 | up |
| ENSSSCG00000022168 | 4.07E-02 | 1.010 | up | ENSSSCG00000017602 | 3.64E-02 | 1.010 | up |
| ENSSSCG00000026547 | 4.05E-02 | 1.011 | up | Sus_newGene_187003 | 3.66E-02 | 1.011 | up |
| Sus_newGene_138878 | 4.82E-02 | 1.012 | up | ENSSSCG00000004506 | 3.36E-02 | 1.012 | up |
| ENSSSCG00000011495 | 4.76E-02 | 1.013 | up | ENSSSCG00000032397 | 3.03E-02 | 1.013 | up |
| ENSSSCG00000002628 | 3.26E-02 | 1.015 | up | ENSSSCG00000013930 | 3.41E-02 | 1.014 | up |
| Sus_newGene_33144 | 3.85E-02 | 1.016 | up | ENSSSCG00000016200 | 3.68E-02 | 1.015 | up |
| ENSSSCG00000025766 | 3.11E-02 | 1.017 | up | Sus_newGene_22845 | 4.81E-02 | 1.017 | up |
| ENSSSCG00000031589 | 3.08E-02 | 1.019 | up | ENSSSCG00000038401 | 3.79E-02 | 1.018 | up |
| ENSSSCG00000039996 | 2.88E-02 | 1.020 | up | ENSSSCG00000039652 | 2.85E-02 | 1.020 | up |
| ENSSSCG00000002355 | 2.77E-02 | 1.023 | up | ENSSSCG00000009620 | 4.99E-02 | 1.021 | up |
| ENSSSCG00000017357 | 3.41E-02 | 1.024 | up | ENSSSCG00000003393 | 3.48E-02 | 1.023 | up |
| ENSSSCG00000002544 | 2.69E-02 | 1.025 | up | ENSSSCG00000022895 | 4.27E-02 | 1.024 | up |
| ENSSSCG00000036938 | 2.55E-02 | 1.026 | up | ENSSSCG00000004328 | 4.10E-02 | 1.025 | up |
| ENSSSCG00000004718 | 2.85E-02 | 1.026 | up | Sus_newGene_105079 | 2.95E-02 | 1.026 | up |
| ENSSSCG00000038650 | 4.06E-02 | 1.027 | up | ENSSSCG00000028481 | 2.96E-02 | 1.027 | up |
| ENSSSCG00000003622 | 2.95E-02 | 1.027 | up | ENSSSCG00000035669 | 2.69E-02 | 1.027 | up |
| ENSSSCG00000007544 | 3.12E-02 | 1.028 | up | ENSSSCG00000026344 | 3.35E-02 | 1.028 | up |
| ENSSSCG00000034763 | 2.46E-02 | 1.029 | up | ENSSSCG00000031159 | 2.73E-02 | 1.029 | up |
| ENSSSCG00000011025 | 3.02E-02 | 1.030 | up | ENSSSCG00000015667 | 3.47E-02 | 1.030 | up |
| ENSSSCG00000004792 | 2.54E-02 | 1.031 | up | ENSSSCG00000003352 | 2.45E-02 | 1.030 | up |
| ENSSSCG00000010107 | 2.37E-02 | 1.032 | up | ENSSSCG00000015541 | 2.73E-02 | 1.031 | up |
| ENSSSCG00000005749 | 2.39E-02 | 1.033 | up | ENSSSCG00000004897 | 2.64E-02 | 1.032 | up |
| ENSSSCG00000021874 | 2.80E-02 | 1.035 | up | ENSSSCG00000016843 | 3.49E-02 | 1.034 | up |
| ENSSSCG00000008690 | 2.23E-02 | 1.037 | up | ENSSSCG00000036679 | 4.56E-02 | 1.035 | up |
| ENSSSCG00000008029 | 2.27E-02 | 1.039 | up | ENSSSCG00000017160 | 2.22E-02 | 1.038 | up |
| ENSSSCG00000004538 | 2.68E-02 | 1.039 | up | ENSSSCG00000016225 | 2.26E-02 | 1.039 | up |
| ENSSSCG00000011313 | 3.42E-02 | 1.039 | up | Sus_newGene_138869 | 2.30E-02 | 1.039 | up |
| ENSSSCG00000027098 | 2.19E-02 | 1.040 | up | ENSSSCG00000032984 | 2.14E-02 | 1.040 | up |
| ENSSSCG00000017116 | 2.77E-02 | 1.041 | up | ENSSSCG00000017201 | 4.77E-02 | 1.040 | up |
| ENSSSCG00000040735 | 4.03E-02 | 1.044 | up | ENSSSCG00000028762 | 3.10E-02 | 1.044 | up |
| ENSSSCG00000017477 | 2.21E-02 | 1.044 | up | ENSSSCG00000030707 | 2.05E-02 | 1.044 | up |
| ENSSSCG00000002690 | 2.01E-02 | 1.045 | up | ENSSSCG00000008056 | 2.53E-02 | 1.044 | up |
| ENSSSCG00000002755 | 2.84E-02 | 1.047 | up | ENSSSCG00000017156 | 2.36E-02 | 1.045 | up |
| ENSSSCG00000007153 | 3.66E-02 | 1.049 | up | ENSSSCG00000002350 | 2.34E-02 | 1.048 | up |
| ENSSSCG00000012967 | 4.80E-02 | 1.052 | up | ENSSSCG00000010493 | 4.75E-02 | 1.049 | up |
| ENSSSCG00000026863 | 3.11E-02 | 1.054 | up | ENSSSCG00000038250 | 2.99E-02 | 1.053 | up |
| Sus_newGene_16425 | 1.78E-02 | 1.055 | up | Sus_newGene_126464 | 2.41E-02 | 1.055 | up |
| ENSSSCG00000039757 | 1.96E-02 | 1.056 | up | ENSSSCG00000017272 | 2.51E-02 | 1.055 | up |
| ENSSSCG00000023174 | 2.29E-02 | 1.058 | up | ENSSSCG00000028513 | 1.97E-02 | 1.058 | up |
| ENSSSCG00000008644 | 3.82E-02 | 1.059 | up | ENSSSCG00000015045 | 4.42E-02 | 1.058 | up |
| ENSSSCG00000000612 | 1.87E-02 | 1.060 | up | ENSSSCG00000037516 | 2.11E-02 | 1.059 | up |
| ENSSSCG00000012315 | 2.44E-02 | 1.061 | up | ENSSSCG00000005045 | 1.64E-02 | 1.060 | up |
| ENSSSCG00000002739 | 1.64E-02 | 1.063 | up | ENSSSCG00000035621 | 1.62E-02 | 1.061 | up |
| ENSSSCG00000001697 | 1.91E-02 | 1.064 | up | ENSSSCG00000002142 | 2.15E-02 | 1.064 | up |
| ENSSSCG00000016898 | 1.54E-02 | 1.066 | up | ENSSSCG00000007586 | 4.12E-02 | 1.065 | up |
| ENSSSCG00000002931 | 4.84E-02 | 1.066 | up | ENSSSCG00000017623 | 1.72E-02 | 1.066 | up |
| ENSSSCG00000025108 | 2.12E-02 | 1.069 | up | Sus_newGene_60268 | 1.55E-02 | 1.068 | up |
| ENSSSCG00000013278 | 1.46E-02 | 1.069 | up | ENSSSCG00000005585 | 1.54E-02 | 1.069 | up |
| ENSSSCG00000012317 | 1.81E-02 | 1.070 | up | ENSSSCG00000034765 | 1.53E-02 | 1.070 | up |
| ENSSSCG00000021149 | 1.46E-02 | 1.071 | up | ENSSSCG00000011882 | 3.50E-02 | 1.071 | up |
| ENSSSCG00000011843 | 3.07E-02 | 1.072 | up | ENSSSCG00000000493 | 2.01E-02 | 1.071 | up |
| ENSSSCG00000026383 | 2.99E-02 | 1.072 | up | ENSSSCG00000021647 | 2.05E-02 | 1.072 | up |
| ENSSSCG00000015859 | 1.62E-02 | 1.073 | up | ENSSSCG00000005598 | 2.03E-02 | 1.072 | up |
| ENSSSCG00000011896 | 1.50E-02 | 1.074 | up | ENSSSCG00000009071 | 1.59E-02 | 1.074 | up |
| ENSSSCG00000037416 | 2.59E-02 | 1.075 | up | ENSSSCG00000033106 | 1.36E-02 | 1.075 | up |
| Sus_newGene_167429 | 1.40E-02 | 1.076 | up | Sus_newGene_129179 | 1.43E-02 | 1.076 | up |
| ENSSSCG00000000837 | 1.43E-02 | 1.079 | up | ENSSSCG00000001201 | 1.51E-02 | 1.078 | up |
| ENSSSCG00000009971 | 1.86E-02 | 1.080 | up | ENSSSCG00000014044 | 2.00E-02 | 1.079 | up |
| Sus_newGene_200526 | 2.55E-02 | 1.081 | up | ENSSSCG00000040793 | 3.59E-02 | 1.080 | up |
| ENSSSCG00000024975 | 1.71E-02 | 1.081 | up | ENSSSCG00000036520 | 1.33E-02 | 1.081 | up |
| ENSSSCG00000022993 | 1.22E-02 | 1.082 | up | ENSSSCG00000006917 | 1.41E-02 | 1.082 | up |
| ENSSSCG00000003532 | 2.64E-02 | 1.083 | up | ENSSSCG00000029485 | 1.55E-02 | 1.083 | up |
| Sus_newGene_16439 | 1.54E-02 | 1.084 | up | ENSSSCG00000003093 | 1.20E-02 | 1.084 | up |
| ENSSSCG00000005484 | 1.78E-02 | 1.085 | up | ENSSSCG00000013895 | 3.42E-02 | 1.084 | up |
| ENSSSCG00000040183 | 2.35E-02 | 1.086 | up | ENSSSCG00000016111 | 3.10E-02 | 1.086 | up |
| ENSSSCG00000011594 | 1.37E-02 | 1.089 | up | ENSSSCG00000030798 | 1.41E-02 | 1.087 | up |
| ENSSSCG00000024043 | 1.71E-02 | 1.090 | up | ENSSSCG00000034058 | 1.80E-02 | 1.089 | up |
| ENSSSCG00000029168 | 1.42E-02 | 1.091 | up | ENSSSCG00000011791 | 1.79E-02 | 1.090 | up |
| ENSSSCG00000014364 | 1.96E-02 | 1.092 | up | ENSSSCG00000022661 | 1.34E-02 | 1.091 | up |
| ENSSSCG00000006737 | 1.31E-02 | 1.093 | up | ENSSSCG00000012270 | 2.39E-02 | 1.092 | up |
| ENSSSCG00000013583 | 4.16E-02 | 1.094 | up | ENSSSCG00000012108 | 1.20E-02 | 1.094 | up |
| ENSSSCG00000028249 | 1.42E-02 | 1.095 | up | ENSSSCG00000003233 | 3.79E-02 | 1.095 | up |
| ENSSSCG00000025182 | 1.38E-02 | 1.099 | up | ENSSSCG00000008629 | 1.85E-02 | 1.099 | up |
| ENSSSCG00000002287 | 1.05E-02 | 1.100 | up | Sus_newGene_95722 | 3.08E-02 | 1.099 | up |
| ENSSSCG00000029837 | 1.17E-02 | 1.101 | up | ENSSSCG00000001709 | 9.63E-03 | 1.101 | up |
| ENSSSCG00000014252 | 1.15E-02 | 1.103 | up | ENSSSCG00000012371 | 9.82E-03 | 1.102 | up |
| ENSSSCG00000005506 | 1.08E-02 | 1.104 | up | ENSSSCG00000011493 | 9.22E-03 | 1.104 | up |
| ENSSSCG00000034491 | 1.29E-02 | 1.105 | up | ENSSSCG00000005593 | 1.17E-02 | 1.104 | up |
| ENSSSCG00000003512 | 1.88E-02 | 1.105 | up | ENSSSCG00000004955 | 9.00E-03 | 1.105 | up |
| ENSSSCG00000016147 | 1.22E-02 | 1.106 | up | Sus_newGene_117651 | 9.78E-03 | 1.105 | up |
| Sus_newGene_105816 | 1.58E-02 | 1.109 | up | ENSSSCG00000032632 | 9.93E-03 | 1.109 | up |
| ENSSSCG00000023279 | 1.20E-02 | 1.109 | up | Sus_newGene_124732 | 2.55E-02 | 1.109 | up |
| ENSSSCG00000002799 | 1.63E-02 | 1.111 | up | ENSSSCG00000017866 | 1.15E-02 | 1.110 | up |
| ENSSSCG00000006453 | 1.26E-02 | 1.113 | up | ENSSSCG00000002383 | 1.74E-02 | 1.112 | up |
| ENSSSCG00000001873 | 7.87E-03 | 1.115 | up | ENSSSCG00000000293 | 9.30E-03 | 1.114 | up |
| ENSSSCG00000029082 | 1.27E-02 | 1.117 | up | ENSSSCG00000002872 | 9.44E-03 | 1.117 | up |
| ENSSSCG00000003672 | 2.34E-02 | 1.119 | up | ENSSSCG00000009834 | 1.01E-02 | 1.117 | up |
| ENSSSCG00000003654 | 4.22E-02 | 1.119 | up | ENSSSCG00000023806 | 7.43E-03 | 1.119 | up |
| ENSSSCG00000006344 | 7.28E-03 | 1.121 | up | ENSSSCG00000009128 | 7.29E-03 | 1.120 | up |
| ENSSSCG00000006493 | 2.84E-02 | 1.122 | up | ENSSSCG00000003699 | 8.42E-03 | 1.121 | up |
| ENSSSCG00000006321 | 1.88E-02 | 1.122 | up | ENSSSCG00000007023 | 7.90E-03 | 1.122 | up |
| ENSSSCG00000007458 | 8.92E-03 | 1.125 | up | ENSSSCG00000005607 | 1.23E-02 | 1.124 | up |
| ENSSSCG00000003881 | 7.04E-03 | 1.128 | up | ENSSSCG00000009759 | 8.85E-03 | 1.127 | up |
| ENSSSCG00000004999 | 7.36E-03 | 1.129 | up | ENSSSCG00000031866 | 1.15E-02 | 1.128 | up |
| ENSSSCG00000028974 | 1.29E-02 | 1.129 | up | ENSSSCG00000036139 | 6.45E-03 | 1.129 | up |
| ENSSSCG00000005716 | 6.31E-03 | 1.131 | up | ENSSSCG00000008724 | 6.42E-03 | 1.129 | up |
| ENSSSCG00000008735 | 7.35E-03 | 1.131 | up | ENSSSCG00000008966 | 8.23E-03 | 1.131 | up |
| ENSSSCG00000005343 | 1.03E-02 | 1.132 | up | ENSSSCG00000010854 | 6.46E-03 | 1.132 | up |
| ENSSSCG00000006385 | 8.00E-03 | 1.133 | up | ENSSSCG00000021203 | 7.01E-03 | 1.133 | up |
| ENSSSCG00000009569 | 1.15E-02 | 1.134 | up | ENSSSCG00000011360 | 6.34E-03 | 1.133 | up |
| ENSSSCG00000028225 | 1.18E-02 | 1.135 | up | ENSSSCG00000012771 | 7.95E-03 | 1.134 | up |
| ENSSSCG00000040163 | 6.00E-03 | 1.135 | up | ENSSSCG00000024592 | 1.20E-02 | 1.135 | up |
| ENSSSCG00000006553 | 7.40E-03 | 1.137 | up | ENSSSCG00000024065 | 5.81E-03 | 1.136 | up |
| ENSSSCG00000034581 | 6.34E-03 | 1.138 | up | ENSSSCG00000017877 | 8.07E-03 | 1.138 | up |
| ENSSSCG00000012007 | 6.66E-03 | 1.139 | up | ENSSSCG00000011548 | 5.62E-03 | 1.139 | up |
| ENSSSCG00000027251 | 1.95E-02 | 1.139 | up | ENSSSCG00000000146 | 2.06E-02 | 1.139 | up |
| ENSSSCG00000023820 | 5.62E-03 | 1.140 | up | ENSSSCG00000002645 | 5.58E-03 | 1.140 | up |
| ENSSSCG00000014136 | 2.99E-02 | 1.140 | up | ENSSSCG00000003379 | 7.02E-03 | 1.140 | up |
| ENSSSCG00000009781 | 5.45E-03 | 1.141 | up | ENSSSCG00000032936 | 5.78E-03 | 1.141 | up |
| ENSSSCG00000006911 | 2.07E-02 | 1.141 | up | ENSSSCG00000011245 | 5.50E-03 | 1.141 | up |
| ENSSSCG00000040355 | 4.42E-02 | 1.143 | up | Sus_newGene_35349 | 7.58E-03 | 1.142 | up |
| ENSSSCG00000010949 | 5.88E-03 | 1.144 | up | ENSSSCG00000014828 | 7.41E-03 | 1.144 | up |
| ENSSSCG00000009943 | 7.00E-03 | 1.145 | up | ENSSSCG00000006506 | 5.15E-03 | 1.145 | up |
| ENSSSCG00000016379 | 1.28E-02 | 1.145 | up | ENSSSCG00000038853 | 5.77E-03 | 1.145 | up |
| ENSSSCG00000021731 | 7.69E-03 | 1.148 | up | ENSSSCG00000002260 | 7.10E-03 | 1.147 | up |
| ENSSSCG00000021821 | 1.23E-02 | 1.150 | up | Sus_newGene_58149 | 4.85E-03 | 1.149 | up |
| ENSSSCG00000011714 | 5.85E-03 | 1.151 | up | ENSSSCG00000009370 | 1.38E-02 | 1.150 | up |
| ENSSSCG00000014291 | 1.05E-02 | 1.151 | up | ENSSSCG00000007839 | 6.91E-03 | 1.151 | up |
| ENSSSCG00000031426 | 5.88E-03 | 1.151 | up | ENSSSCG00000015116 | 4.70E-03 | 1.151 | up |
| ENSSSCG00000008540 | 9.61E-03 | 1.152 | up | ENSSSCG00000028542 | 5.88E-03 | 1.151 | up |
| ENSSSCG00000038597 | 4.49E-02 | 1.155 | up | ENSSSCG00000032266 | 4.57E-03 | 1.153 | up |
| ENSSSCG00000008796 | 8.44E-03 | 1.156 | up | ENSSSCG00000032831 | 8.09E-03 | 1.155 | up |
| ENSSSCG00000022208 | 5.43E-03 | 1.157 | up | ENSSSCG00000039658 | 4.37E-03 | 1.156 | up |
| ENSSSCG00000009898 | 7.20E-03 | 1.158 | up | Sus_newGene_31161 | 2.75E-02 | 1.158 | up |
| ENSSSCG00000008989 | 4.40E-03 | 1.160 | up | ENSSSCG00000030467 | 6.57E-03 | 1.159 | up |
| ENSSSCG00000030294 | 4.15E-03 | 1.160 | up | ENSSSCG00000012650 | 4.43E-03 | 1.160 | up |
| ENSSSCG00000016743 | 8.55E-03 | 1.161 | up | ENSSSCG00000021534 | 2.33E-02 | 1.161 | up |
| ENSSSCG00000029029 | 8.04E-03 | 1.162 | up | ENSSSCG00000002829 | 1.02E-02 | 1.161 | up |
| ENSSSCG00000026590 | 5.16E-03 | 1.163 | up | ENSSSCG00000031796 | 4.53E-03 | 1.163 | up |
| ENSSSCG00000007558 | 4.47E-03 | 1.164 | up | ENSSSCG00000007355 | 5.92E-03 | 1.163 | up |
| ENSSSCG00000031492 | 4.35E-03 | 1.164 | up | ENSSSCG00000024341 | 4.69E-03 | 1.164 | up |
| ENSSSCG00000022202 | 5.22E-03 | 1.165 | up | ENSSSCG00000011377 | 8.00E-03 | 1.165 | up |
| ENSSSCG00000001654 | 3.77E-03 | 1.167 | up | ENSSSCG00000009142 | 4.28E-03 | 1.166 | up |
| ENSSSCG00000001912 | 4.01E-03 | 1.167 | up | ENSSSCG00000029533 | 4.64E-03 | 1.167 | up |
| ENSSSCG00000001435 | 4.23E-03 | 1.168 | up | Sus_newGene_131825 | 3.73E-03 | 1.167 | up |
| ENSSSCG00000001653 | 3.61E-03 | 1.170 | up | ENSSSCG00000003489 | 4.79E-03 | 1.169 | up |
| ENSSSCG00000029039 | 3.53E-03 | 1.171 | up | ENSSSCG00000023362 | 6.70E-03 | 1.171 | up |
| ENSSSCG00000014956 | 4.12E-03 | 1.172 | up | ENSSSCG00000014314 | 3.73E-02 | 1.172 | up |
| ENSSSCG00000013876 | 5.44E-03 | 1.173 | up | ENSSSCG00000010640 | 2.09E-02 | 1.173 | up |
| ENSSSCG00000011496 | 5.30E-03 | 1.174 | up | ENSSSCG00000007830 | 4.63E-03 | 1.173 | up |
| ENSSSCG00000012335 | 4.48E-03 | 1.176 | up | ENSSSCG00000004823 | 3.68E-03 | 1.175 | up |
| ENSSSCG00000009653 | 1.31E-02 | 1.179 | up | ENSSSCG00000009681 | 4.14E-03 | 1.178 | up |
| ENSSSCG00000010169 | 3.63E-03 | 1.179 | up | ENSSSCG00000011752 | 8.04E-03 | 1.179 | up |
| ENSSSCG00000001410 | 3.97E-03 | 1.181 | up | ENSSSCG00000001577 | 4.21E-03 | 1.181 | up |
| Sus_newGene_105106 | 1.47E-02 | 1.182 | up | ENSSSCG00000000110 | 3.33E-03 | 1.182 | up |
| ENSSSCG00000013566 | 4.68E-03 | 1.183 | up | ENSSSCG00000012731 | 4.99E-03 | 1.182 | up |
| ENSSSCG00000015988 | 4.55E-03 | 1.186 | up | ENSSSCG00000001544 | 4.00E-03 | 1.185 | up |
| ENSSSCG00000008392 | 1.62E-02 | 1.186 | up | Sus_newGene_17062 | 1.62E-02 | 1.186 | up |
| ENSSSCG00000010686 | 6.56E-03 | 1.187 | up | ENSSSCG00000014207 | 4.87E-03 | 1.186 | up |
| ENSSSCG00000010536 | 3.29E-03 | 1.188 | up | Sus_newGene_105068 | 3.36E-03 | 1.187 | up |
| ENSSSCG00000007360 | 2.92E-03 | 1.189 | up | ENSSSCG00000013079 | 5.86E-03 | 1.188 | up |
| ENSSSCG00000039094 | 6.15E-03 | 1.191 | up | ENSSSCG00000014920 | 4.07E-03 | 1.189 | up |
| ENSSSCG00000006767 | 2.76E-03 | 1.192 | up | ENSSSCG00000001695 | 2.70E-03 | 1.191 | up |
| ENSSSCG00000008079 | 5.25E-03 | 1.193 | up | ENSSSCG00000039615 | 3.06E-03 | 1.193 | up |
| ENSSSCG00000020876 | 3.45E-03 | 1.194 | up | ENSSSCG00000034262 | 6.09E-03 | 1.194 | up |
| ENSSSCG00000016794 | 5.26E-03 | 1.195 | up | ENSSSCG00000002451 | 9.86E-03 | 1.195 | up |
| ENSSSCG00000003513 | 2.97E-03 | 1.195 | up | ENSSSCG00000012542 | 3.06E-03 | 1.195 | up |
| ENSSSCG00000038948 | 2.38E-03 | 1.198 | up | Sus_newGene_21139 | 3.77E-02 | 1.195 | up |
| ENSSSCG00000006274 | 2.73E-03 | 1.198 | up | ENSSSCG00000032877 | 2.91E-03 | 1.198 | up |
| ENSSSCG00000005965 | 2.35E-03 | 1.199 | up | ENSSSCG00000000215 | 2.42E-03 | 1.198 | up |
| ENSSSCG00000003895 | 2.75E-03 | 1.199 | up | ENSSSCG00000007454 | 2.60E-03 | 1.199 | up |
| ENSSSCG00000030155 | 2.28E-03 | 1.200 | up | ENSSSCG00000000403 | 2.43E-03 | 1.200 | up |
| ENSSSCG00000016417 | 2.35E-03 | 1.201 | up | Sus_newGene_162047 | 6.88E-03 | 1.200 | up |
| ENSSSCG00000034259 | 3.18E-03 | 1.202 | up | ENSSSCG00000038509 | 4.15E-03 | 1.202 | up |
| ENSSSCG00000016873 | 4.76E-02 | 1.204 | up | ENSSSCG00000008624 | 2.24E-03 | 1.203 | up |
| ENSSSCG00000029771 | 2.64E-03 | 1.204 | up | ENSSSCG00000032407 | 2.24E-02 | 1.204 | up |
| ENSSSCG00000006740 | 7.68E-03 | 1.207 | up | ENSSSCG00000032947 | 2.47E-03 | 1.205 | up |
| ENSSSCG00000031249 | 4.25E-03 | 1.210 | up | ENSSSCG00000000478 | 2.45E-03 | 1.208 | up |
| ENSSSCG00000007477 | 4.69E-03 | 1.212 | up | ENSSSCG00000012996 | 4.35E-03 | 1.211 | up |
| ENSSSCG00000021068 | 1.98E-03 | 1.212 | up | ENSSSCG00000016420 | 2.74E-03 | 1.212 | up |
| Sus_newGene_162067 | 3.32E-03 | 1.213 | up | ENSSSCG00000036402 | 4.22E-03 | 1.213 | up |
| ENSSSCG00000029745 | 2.68E-03 | 1.216 | up | ENSSSCG00000030947 | 4.35E-03 | 1.215 | up |
| ENSSSCG00000021285 | 2.13E-03 | 1.217 | up | ENSSSCG00000024481 | 2.96E-03 | 1.217 | up |
| ENSSSCG00000017164 | 3.52E-03 | 1.218 | up | ENSSSCG00000016816 | 1.28E-02 | 1.217 | up |
| ENSSSCG00000022636 | 2.12E-03 | 1.219 | up | ENSSSCG00000014119 | 4.05E-03 | 1.219 | up |
| ENSSSCG00000013900 | 1.68E-03 | 1.220 | up | ENSSSCG00000034348 | 1.95E-03 | 1.220 | up |
| ENSSSCG00000023569 | 3.68E-02 | 1.223 | up | ENSSSCG00000023026 | 2.51E-03 | 1.221 | up |
| ENSSSCG00000008382 | 3.14E-03 | 1.224 | up | ENSSSCG00000015411 | 1.96E-03 | 1.223 | up |
| Sus_newGene_186679 | 1.59E-03 | 1.226 | up | ENSSSCG00000032360 | 1.72E-03 | 1.226 | up |
| ENSSSCG00000016174 | 6.41E-03 | 1.228 | up | ENSSSCG00000011111 | 1.87E-03 | 1.227 | up |
| ENSSSCG00000007466 | 1.52E-03 | 1.228 | up | ENSSSCG00000040349 | 1.57E-03 | 1.228 | up |
| ENSSSCG00000026506 | 1.55E-03 | 1.230 | up | ENSSSCG00000012250 | 1.55E-03 | 1.229 | up |
| ENSSSCG00000038253 | 1.57E-03 | 1.231 | up | ENSSSCG00000013771 | 1.51E-03 | 1.231 | up |
| ENSSSCG00000003438 | 2.80E-03 | 1.233 | up | ENSSSCG00000011237 | 3.52E-03 | 1.231 | up |
| Sus_newGene_53875 | 3.99E-02 | 1.235 | up | ENSSSCG00000003264 | 2.43E-03 | 1.235 | up |
| ENSSSCG00000016604 | 1.82E-03 | 1.236 | up | ENSSSCG00000015555 | 6.04E-03 | 1.236 | up |
| ENSSSCG00000007599 | 1.39E-03 | 1.238 | up | ENSSSCG00000001025 | 1.95E-03 | 1.237 | up |
| ENSSSCG00000009473 | 2.43E-03 | 1.238 | up | ENSSSCG00000008236 | 2.32E-03 | 1.238 | up |
| ENSSSCG00000015111 | 4.37E-03 | 1.239 | up | ENSSSCG00000016409 | 1.49E-03 | 1.238 | up |
| ENSSSCG00000008991 | 2.23E-03 | 1.240 | up | ENSSSCG00000031706 | 4.48E-03 | 1.240 | up |
| Sus_newGene_545 | 3.16E-03 | 1.240 | up | ENSSSCG00000002254 | 1.31E-03 | 1.240 | up |
| ENSSSCG00000003746 | 2.49E-02 | 1.240 | up | ENSSSCG00000009023 | 1.45E-03 | 1.240 | up |
| ENSSSCG00000001507 | 1.27E-03 | 1.241 | up | ENSSSCG00000017604 | 2.57E-02 | 1.241 | up |
| ENSSSCG00000017754 | 2.95E-02 | 1.243 | up | ENSSSCG00000029367 | 1.88E-03 | 1.243 | up |
| ENSSSCG00000015664 | 1.18E-03 | 1.243 | up | ENSSSCG00000009157 | 1.35E-03 | 1.243 | up |
| ENSSSCG00000029430 | 1.24E-03 | 1.245 | up | ENSSSCG00000014794 | 1.88E-03 | 1.243 | up |
| ENSSSCG00000023603 | 8.57E-03 | 1.249 | up | ENSSSCG00000021036 | 5.43E-03 | 1.245 | up |
| ENSSSCG00000013333 | 1.12E-03 | 1.250 | up | ENSSSCG00000014820 | 1.13E-03 | 1.250 | up |
| ENSSSCG00000012203 | 1.13E-03 | 1.250 | up | ENSSSCG00000022486 | 1.83E-03 | 1.250 | up |
| ENSSSCG00000016548 | 1.50E-03 | 1.251 | up | Sus_newGene_22841 | 1.45E-03 | 1.250 | up |
| ENSSSCG00000005498 | 2.75E-03 | 1.251 | up | ENSSSCG00000011540 | 1.42E-03 | 1.251 | up |
| ENSSSCG00000008887 | 1.78E-03 | 1.256 | up | ENSSSCG00000006194 | 1.11E-03 | 1.252 | up |
| ENSSSCG00000004249 | 1.19E-03 | 1.257 | up | ENSSSCG00000010329 | 1.40E-03 | 1.256 | up |
| ENSSSCG00000012267 | 1.07E-03 | 1.260 | up | ENSSSCG00000009657 | 1.03E-03 | 1.258 | up |
| ENSSSCG00000011047 | 9.63E-04 | 1.264 | up | ENSSSCG00000032049 | 1.13E-03 | 1.264 | up |
| ENSSSCG00000032620 | 1.04E-03 | 1.266 | up | ENSSSCG00000006482 | 8.77E-04 | 1.266 | up |
| Sus_newGene_131430 | 2.82E-03 | 1.267 | up | ENSSSCG00000014960 | 8.23E-04 | 1.267 | up |
| ENSSSCG00000035442 | 8.17E-04 | 1.267 | up | ENSSSCG00000006508 | 1.16E-03 | 1.267 | up |
| ENSSSCG00000009642 | 1.25E-03 | 1.269 | up | Sus_newGene_22840 | 9.15E-04 | 1.269 | up |
| ENSSSCG00000016510 | 7.88E-04 | 1.271 | up | ENSSSCG00000011497 | 9.10E-04 | 1.270 | up |
| ENSSSCG00000005426 | 9.42E-04 | 1.271 | up | ENSSSCG00000003068 | 4.82E-03 | 1.271 | up |
| ENSSSCG00000032838 | 1.02E-03 | 1.273 | up | ENSSSCG00000016123 | 8.28E-04 | 1.272 | up |
| ENSSSCG00000008478 | 1.08E-03 | 1.274 | up | ENSSSCG00000024813 | 7.58E-04 | 1.273 | up |
| ENSSSCG00000016501 | 7.30E-04 | 1.275 | up | ENSSSCG00000028239 | 8.23E-04 | 1.275 | up |
| ENSSSCG00000032705 | 1.12E-03 | 1.277 | up | ENSSSCG00000009048 | 7.32E-04 | 1.276 | up |
| ENSSSCG00000037015 | 1.63E-03 | 1.277 | up | ENSSSCG00000024152 | 9.22E-04 | 1.277 | up |
| Sus_newGene_143458 | 1.52E-03 | 1.280 | up | ENSSSCG00000010062 | 7.36E-04 | 1.278 | up |
| ENSSSCG00000016258 | 3.15E-02 | 1.282 | up | ENSSSCG00000038521 | 1.02E-03 | 1.281 | up |
| Sus_newGene_19799 | 3.57E-02 | 1.286 | up | ENSSSCG00000038089 | 9.54E-04 | 1.285 | up |
| Sus_newGene_158478 | 3.82E-03 | 1.287 | up | ENSSSCG00000026569 | 6.12E-04 | 1.286 | up |
| ENSSSCG00000025565 | 9.05E-04 | 1.288 | up | ENSSSCG00000026457 | 6.71E-03 | 1.287 | up |
| ENSSSCG00000033010 | 2.85E-03 | 1.289 | up | ENSSSCG00000029843 | 5.55E-03 | 1.288 | up |
| ENSSSCG00000032878 | 2.48E-02 | 1.289 | up | ENSSSCG00000006709 | 2.02E-03 | 1.289 | up |
| ENSSSCG00000027407 | 3.07E-03 | 1.292 | up | ENSSSCG00000009007 | 5.90E-04 | 1.292 | up |
| ENSSSCG00000003017 | 7.09E-04 | 1.294 | up | ENSSSCG00000030130 | 1.17E-03 | 1.293 | up |
| ENSSSCG00000010212 | 9.90E-04 | 1.294 | up | ENSSSCG00000013124 | 5.73E-04 | 1.294 | up |
| ENSSSCG00000020813 | 5.30E-04 | 1.297 | up | ENSSSCG00000038082 | 5.43E-04 | 1.294 | up |
| Sus_newGene_142330 | 1.77E-03 | 1.298 | up | ENSSSCG00000023956 | 9.74E-04 | 1.298 | up |
| Sus_newGene_183398 | 5.05E-04 | 1.298 | up | ENSSSCG00000008202 | 6.58E-04 | 1.298 | up |
| Sus_newGene_131434 | 7.95E-04 | 1.300 | up | Sus_newGene_183105 | 1.09E-02 | 1.298 | up |
| ENSSSCG00000004854 | 1.21E-03 | 1.303 | up | ENSSSCG00000034502 | 7.80E-04 | 1.302 | up |
| Sus_newGene_133922 | 8.76E-04 | 1.304 | up | ENSSSCG00000002444 | 5.16E-04 | 1.304 | up |
| ENSSSCG00000010586 | 4.47E-04 | 1.306 | up | ENSSSCG00000014792 | 4.34E-04 | 1.306 | up |
| ENSSSCG00000007574 | 3.31E-03 | 1.310 | up | Sus_newGene_42057 | 1.87E-02 | 1.308 | up |
| ENSSSCG00000021862 | 4.33E-04 | 1.311 | up | Sus_newGene_35357 | 1.11E-03 | 1.311 | up |
| ENSSSCG00000035798 | 4.03E-04 | 1.312 | up | ENSSSCG00000033708 | 4.74E-04 | 1.312 | up |
| ENSSSCG00000017165 | 5.26E-04 | 1.313 | up | ENSSSCG00000012564 | 8.02E-04 | 1.312 | up |
| ENSSSCG00000012284 | 6.07E-04 | 1.314 | up | ENSSSCG00000032444 | 1.43E-03 | 1.313 | up |
| ENSSSCG00000004055 | 1.20E-02 | 1.317 | up | ENSSSCG00000000504 | 1.20E-02 | 1.317 | up |
| ENSSSCG00000002910 | 1.98E-03 | 1.317 | up | Sus_newGene_79868 | 1.33E-03 | 1.317 | up |
| Sus_newGene_120784 | 3.64E-03 | 1.319 | up | ENSSSCG00000003016 | 4.68E-04 | 1.319 | up |
| ENSSSCG00000029185 | 5.09E-04 | 1.324 | up | ENSSSCG00000015273 | 6.37E-04 | 1.321 | up |
| ENSSSCG00000002895 | 5.07E-04 | 1.324 | up | ENSSSCG00000008261 | 4.61E-04 | 1.324 | up |
| ENSSSCG00000004678 | 8.36E-04 | 1.325 | up | ENSSSCG00000005455 | 1.48E-03 | 1.324 | up |
| ENSSSCG00000012408 | 3.75E-04 | 1.325 | up | ENSSSCG00000037670 | 3.30E-04 | 1.325 | up |
| ENSSSCG00000034386 | 8.35E-04 | 1.327 | up | ENSSSCG00000000078 | 4.58E-04 | 1.327 | up |
| ENSSSCG00000011471 | 1.14E-03 | 1.329 | up | ENSSSCG00000004789 | 7.62E-03 | 1.328 | up |
| ENSSSCG00000005308 | 3.50E-04 | 1.332 | up | ENSSSCG00000004830 | 5.06E-04 | 1.329 | up |
| ENSSSCG00000035479 | 3.09E-04 | 1.333 | up | Sus_newGene_176716 | 9.03E-04 | 1.333 | up |
| ENSSSCG00000004387 | 3.54E-04 | 1.335 | up | ENSSSCG00000024021 | 2.81E-04 | 1.333 | up |
| ENSSSCG00000016211 | 2.76E-04 | 1.336 | up | ENSSSCG00000009844 | 2.68E-04 | 1.335 | up |
| ENSSSCG00000030420 | 6.41E-04 | 1.336 | up | ENSSSCG00000016920 | 2.68E-04 | 1.336 | up |
| ENSSSCG00000009431 | 2.63E-04 | 1.337 | up | ENSSSCG00000001483 | 8.18E-04 | 1.336 | up |
| ENSSSCG00000013382 | 6.21E-04 | 1.338 | up | ENSSSCG00000017430 | 4.05E-04 | 1.337 | up |
| ENSSSCG00000028446 | 3.16E-04 | 1.339 | up | ENSSSCG00000015582 | 4.43E-04 | 1.338 | up |
| ENSSSCG00000011951 | 2.39E-04 | 1.342 | up | ENSSSCG00000024694 | 3.27E-04 | 1.342 | up |
| Sus_newGene_85300 | 4.95E-04 | 1.343 | up | ENSSSCG00000010514 | 2.38E-04 | 1.343 | up |
| ENSSSCG00000014213 | 1.00E-03 | 1.344 | up | Sus_newGene_187001 | 2.80E-04 | 1.344 | up |
| ENSSSCG00000021843 | 3.94E-02 | 1.345 | up | ENSSSCG00000009004 | 2.78E-04 | 1.344 | up |
| ENSSSCG00000011201 | 2.68E-04 | 1.346 | up | Sus_newGene_76341 | 6.97E-03 | 1.346 | up |
| Sus_newGene_49958 | 6.21E-04 | 1.347 | up | ENSSSCG00000004856 | 1.11E-02 | 1.346 | up |
| ENSSSCG00000022538 | 2.98E-04 | 1.348 | up | ENSSSCG00000034943 | 5.09E-04 | 1.347 | up |
| ENSSSCG00000034379 | 2.17E-04 | 1.348 | up | ENSSSCG00000008642 | 2.18E-04 | 1.348 | up |
| ENSSSCG00000025826 | 6.66E-04 | 1.349 | up | ENSSSCG00000005225 | 7.63E-04 | 1.348 | up |
| ENSSSCG00000034154 | 4.55E-04 | 1.353 | up | ENSSSCG00000011049 | 2.23E-04 | 1.350 | up |
| ENSSSCG00000004064 | 2.16E-04 | 1.354 | up | ENSSSCG00000007727 | 2.08E-04 | 1.353 | up |
| ENSSSCG00000004705 | 3.01E-04 | 1.355 | up | ENSSSCG00000008881 | 3.43E-04 | 1.354 | up |
| ENSSSCG00000002332 | 2.63E-04 | 1.355 | up | ENSSSCG00000031868 | 3.08E-04 | 1.355 | up |
| ENSSSCG00000010494 | 1.88E-04 | 1.358 | up | ENSSSCG00000009864 | 3.22E-04 | 1.356 | up |
| Sus_newGene_126969 | 3.39E-02 | 1.358 | up | ENSSSCG00000005002 | 2.01E-04 | 1.358 | up |
| ENSSSCG00000015281 | 2.22E-04 | 1.362 | up | ENSSSCG00000038452 | 1.80E-04 | 1.359 | up |
| ENSSSCG00000033497 | 2.10E-04 | 1.364 | up | ENSSSCG00000006701 | 1.71E-04 | 1.362 | up |
| ENSSSCG00000006689 | 1.95E-04 | 1.365 | up | ENSSSCG00000008575 | 2.11E-04 | 1.364 | up |
| ENSSSCG00000009910 | 2.92E-04 | 1.365 | up | ENSSSCG00000000509 | 2.13E-04 | 1.365 | up |
| ENSSSCG00000006018 | 1.98E-04 | 1.367 | up | ENSSSCG00000003471 | 1.63E-04 | 1.365 | up |
| ENSSSCG00000033305 | 8.94E-03 | 1.368 | up | ENSSSCG00000031780 | 3.58E-04 | 1.367 | up |
| ENSSSCG00000031141 | 1.52E-04 | 1.370 | up | ENSSSCG00000015010 | 7.20E-03 | 1.368 | up |
| ENSSSCG00000015828 | 4.52E-04 | 1.371 | up | ENSSSCG00000032078 | 2.41E-04 | 1.370 | up |
| Sus_newGene_24055 | 1.65E-04 | 1.371 | up | ENSSSCG00000037591 | 1.47E-04 | 1.371 | up |
| Sus_newGene_138867 | 1.47E-04 | 1.374 | up | ENSSSCG00000037561 | 1.60E-04 | 1.372 | up |
| ENSSSCG00000006231 | 1.41E-04 | 1.375 | up | Sus_newGene_96378 | 1.85E-04 | 1.374 | up |
| Sus_newGene_196484 | 1.24E-02 | 1.375 | up | ENSSSCG00000004896 | 1.60E-04 | 1.375 | up |
| ENSSSCG00000010575 | 1.35E-04 | 1.376 | up | ENSSSCG00000009917 | 1.37E-04 | 1.375 | up |
| ENSSSCG00000006836 | 1.32E-02 | 1.381 | up | ENSSSCG00000016887 | 1.28E-04 | 1.380 | up |
| ENSSSCG00000015853 | 2.21E-04 | 1.384 | up | Sus_newGene_13065 | 1.18E-04 | 1.384 | up |
| ENSSSCG00000022504 | 1.17E-04 | 1.385 | up | ENSSSCG00000011346 | 1.31E-04 | 1.384 | up |
| ENSSSCG00000007981 | 2.74E-04 | 1.385 | up | ENSSSCG00000002425 | 2.07E-04 | 1.385 | up |
| ENSSSCG00000013773 | 2.15E-04 | 1.385 | up | ENSSSCG00000021704 | 1.44E-04 | 1.385 | up |
| ENSSSCG00000005935 | 1.20E-04 | 1.387 | up | ENSSSCG00000013633 | 1.13E-04 | 1.387 | up |
| ENSSSCG00000026042 | 4.94E-04 | 1.387 | up | ENSSSCG00000003107 | 1.96E-04 | 1.387 | up |
| ENSSSCG00000017139 | 1.13E-04 | 1.389 | up | ENSSSCG00000030857 | 1.09E-04 | 1.389 | up |
| ENSSSCG00000007540 | 3.84E-04 | 1.390 | up | ENSSSCG00000009226 | 1.08E-04 | 1.389 | up |
| ENSSSCG00000032531 | 2.09E-04 | 1.391 | up | ENSSSCG00000005098 | 1.38E-04 | 1.391 | up |
| ENSSSCG00000011364 | 1.69E-04 | 1.392 | up | ENSSSCG00000009114 | 3.81E-04 | 1.391 | up |
| ENSSSCG00000038471 | 4.23E-04 | 1.394 | up | ENSSSCG00000015820 | 1.13E-04 | 1.394 | up |
| ENSSSCG00000038410 | 1.37E-04 | 1.394 | up | ENSSSCG00000011333 | 1.77E-04 | 1.394 | up |
| ENSSSCG00000038730 | 1.65E-04 | 1.397 | up | ENSSSCG00000013432 | 1.07E-04 | 1.395 | up |
| ENSSSCG00000005715 | 1.00E-04 | 1.398 | up | ENSSSCG00000009111 | 2.37E-04 | 1.398 | up |
| ENSSSCG00000006461 | 9.79E-05 | 1.399 | up | ENSSSCG00000031733 | 1.52E-04 | 1.398 | up |
| ENSSSCG00000012885 | 9.02E-05 | 1.399 | up | Sus_newGene_85308 | 1.66E-04 | 1.399 | up |
| ENSSSCG00000004081 | 2.40E-04 | 1.401 | up | ENSSSCG00000008504 | 3.56E-04 | 1.399 | up |
| ENSSSCG00000038604 | 8.67E-05 | 1.403 | up | ENSSSCG00000009650 | 1.36E-04 | 1.402 | up |
| ENSSSCG00000004138 | 8.65E-05 | 1.404 | up | ENSSSCG00000025134 | 1.48E-04 | 1.403 | up |
| ENSSSCG00000006475 | 1.35E-03 | 1.407 | up | ENSSSCG00000007954 | 3.31E-04 | 1.404 | up |
| ENSSSCG00000004082 | 1.44E-04 | 1.409 | up | ENSSSCG00000001099 | 1.08E-04 | 1.408 | up |
| ENSSSCG00000024312 | 4.71E-03 | 1.412 | up | ENSSSCG00000027686 | 8.83E-05 | 1.410 | up |
| ENSSSCG00000014868 | 7.49E-05 | 1.415 | up | ENSSSCG00000010238 | 6.98E-05 | 1.414 | up |
| ENSSSCG00000011368 | 1.94E-04 | 1.417 | up | ENSSSCG00000037371 | 3.12E-04 | 1.415 | up |
| ENSSSCG00000002656 | 6.72E-05 | 1.420 | up | ENSSSCG00000014835 | 6.55E-05 | 1.418 | up |
| ENSSSCG00000011918 | 6.08E-05 | 1.423 | up | ENSSSCG00000032157 | 2.13E-04 | 1.422 | up |
| ENSSSCG00000012926 | 5.70E-05 | 1.425 | up | ENSSSCG00000011278 | 6.18E-05 | 1.423 | up |
| ENSSSCG00000032473 | 5.80E-05 | 1.426 | up | ENSSSCG00000003021 | 5.74E-05 | 1.426 | up |
| ENSSSCG00000034090 | 5.40E-05 | 1.429 | up | Sus_newGene_117013 | 2.01E-03 | 1.428 | up |
| Sus_newGene_138999 | 2.27E-02 | 1.433 | up | Sus_newGene_106567 | 9.78E-05 | 1.432 | up |
| ENSSSCG00000034610 | 7.25E-04 | 1.433 | up | ENSSSCG00000032320 | 5.62E-05 | 1.433 | up |
| ENSSSCG00000035424 | 5.34E-05 | 1.435 | up | ENSSSCG00000010092 | 7.86E-05 | 1.435 | up |
| ENSSSCG00000023010 | 6.28E-05 | 1.435 | up | ENSSSCG00000006622 | 4.83E-05 | 1.435 | up |
| ENSSSCG00000012627 | 3.94E-04 | 1.437 | up | ENSSSCG00000004484 | 2.37E-04 | 1.436 | up |
| ENSSSCG00000015434 | 5.38E-05 | 1.438 | up | Sus_newGene_60267 | 6.52E-05 | 1.438 | up |
| ENSSSCG00000039562 | 9.64E-05 | 1.439 | up | ENSSSCG00000039264 | 4.53E-05 | 1.438 | up |
| ENSSSCG00000025598 | 6.24E-05 | 1.439 | up | ENSSSCG00000035733 | 1.06E-04 | 1.439 | up |
| ENSSSCG00000003038 | 4.50E-05 | 1.440 | up | ENSSSCG00000007682 | 2.77E-03 | 1.439 | up |
| ENSSSCG00000035521 | 4.35E-04 | 1.443 | up | ENSSSCG00000024001 | 6.01E-05 | 1.441 | up |
| ENSSSCG00000010316 | 4.31E-05 | 1.444 | up | ENSSSCG00000015519 | 4.54E-05 | 1.444 | up |
| ENSSSCG00000003040 | 7.31E-05 | 1.445 | up | ENSSSCG00000005469 | 4.11E-05 | 1.445 | up |
| ENSSSCG00000001611 | 3.93E-05 | 1.447 | up | ENSSSCG00000015985 | 5.00E-02 | 1.445 | up |
| ENSSSCG00000022227 | 3.87E-05 | 1.447 | up | ENSSSCG00000006567 | 3.59E-04 | 1.447 | up |
| ENSSSCG00000012591 | 3.66E-05 | 1.450 | up | Sus_newGene_95721 | 4.71E-05 | 1.450 | up |
| ENSSSCG00000008993 | 3.76E-05 | 1.451 | up | Sus_newGene_190125 | 1.45E-03 | 1.451 | up |
| ENSSSCG00000000145 | 1.98E-04 | 1.453 | up | ENSSSCG00000027371 | 3.82E-05 | 1.453 | up |
| ENSSSCG00000011257 | 4.80E-04 | 1.455 | up | ENSSSCG00000038180 | 1.10E-04 | 1.454 | up |
| ENSSSCG00000039997 | 5.03E-05 | 1.457 | up | ENSSSCG00000010012 | 5.71E-05 | 1.456 | up |
| ENSSSCG00000000766 | 8.92E-05 | 1.459 | up | ENSSSCG00000022131 | 4.31E-05 | 1.459 | up |
| ENSSSCG00000027952 | 2.88E-04 | 1.461 | up | ENSSSCG00000034524 | 4.38E-05 | 1.459 | up |
| ENSSSCG00000009968 | 5.12E-05 | 1.462 | up | ENSSSCG00000008697 | 3.66E-05 | 1.462 | up |
| ENSSSCG00000003989 | 2.57E-04 | 1.463 | up | ENSSSCG00000016861 | 1.17E-03 | 1.463 | up |
| ENSSSCG00000034879 | 3.11E-05 | 1.465 | up | ENSSSCG00000012396 | 2.96E-05 | 1.463 | up |
| ENSSSCG00000015842 | 2.72E-05 | 1.468 | up | ENSSSCG00000017791 | 3.71E-05 | 1.466 | up |
| ENSSSCG00000013399 | 3.88E-05 | 1.471 | up | ENSSSCG00000017779 | 3.89E-05 | 1.470 | up |
| ENSSSCG00000025245 | 4.95E-04 | 1.475 | up | ENSSSCG00000003872 | 3.20E-05 | 1.472 | up |
| Sus_newGene_124035 | 2.43E-02 | 1.475 | up | ENSSSCG00000009446 | 2.38E-05 | 1.475 | up |
| ENSSSCG00000009234 | 3.29E-05 | 1.477 | up | ENSSSCG00000001532 | 3.00E-05 | 1.476 | up |
| ENSSSCG00000002965 | 5.22E-05 | 1.478 | up | Sus_newGene_194364 | 1.08E-02 | 1.477 | up |
| ENSSSCG00000003736 | 1.99E-03 | 1.480 | up | ENSSSCG00000002526 | 2.17E-05 | 1.479 | up |
| ENSSSCG00000023328 | 1.75E-03 | 1.485 | up | ENSSSCG00000008051 | 2.05E-05 | 1.482 | up |
| ENSSSCG00000017108 | 1.93E-05 | 1.486 | up | ENSSSCG00000000142 | 2.63E-05 | 1.485 | up |
| ENSSSCG00000016763 | 6.90E-04 | 1.487 | up | ENSSSCG00000001021 | 1.94E-05 | 1.486 | up |
| Sus_newGene_144469 | 4.28E-05 | 1.490 | up | ENSSSCG00000021586 | 5.61E-05 | 1.489 | up |
| ENSSSCG00000015115 | 1.85E-05 | 1.491 | up | ENSSSCG00000007681 | 2.50E-05 | 1.491 | up |
| ENSSSCG00000013314 | 2.55E-05 | 1.493 | up | ENSSSCG00000010303 | 3.01E-05 | 1.493 | up |
| ENSSSCG00000010151 | 1.61E-05 | 1.495 | up | ENSSSCG00000035822 | 1.64E-05 | 1.494 | up |
| Sus_newGene_30193 | 2.26E-05 | 1.496 | up | ENSSSCG00000011889 | 2.41E-05 | 1.496 | up |
| ENSSSCG00000015558 | 1.55E-05 | 1.499 | up | ENSSSCG00000003554 | 2.30E-05 | 1.499 | up |
| ENSSSCG00000007317 | 1.45E-05 | 1.501 | up | ENSSSCG00000027016 | 3.98E-04 | 1.500 | up |
| ENSSSCG00000038824 | 1.47E-05 | 1.503 | up | ENSSSCG00000005288 | 1.43E-05 | 1.503 | up |
| ENSSSCG00000028304 | 1.57E-05 | 1.504 | up | ENSSSCG00000005518 | 1.82E-05 | 1.503 | up |
| ENSSSCG00000005194 | 3.30E-05 | 1.507 | up | Sus_newGene_82460 | 5.10E-03 | 1.505 | up |
| ENSSSCG00000004053 | 1.01E-02 | 1.508 | up | ENSSSCG00000015071 | 1.28E-05 | 1.508 | up |
| ENSSSCG00000025483 | 1.21E-05 | 1.511 | up | ENSSSCG00000016062 | 1.51E-05 | 1.510 | up |
| Sus_newGene_116472 | 2.65E-05 | 1.512 | up | Sus_newGene_58631 | 4.59E-04 | 1.511 | up |
| ENSSSCG00000036401 | 6.46E-05 | 1.515 | up | ENSSSCG00000003987 | 1.58E-03 | 1.514 | up |
| ENSSSCG00000004110 | 1.12E-05 | 1.517 | up | ENSSSCG00000010272 | 1.29E-05 | 1.517 | up |
| ENSSSCG00000016810 | 1.70E-05 | 1.518 | up | ENSSSCG00000003158 | 1.07E-05 | 1.518 | up |
| ENSSSCG00000039009 | 2.36E-05 | 1.520 | up | Sus_newGene_86104 | 3.80E-04 | 1.518 | up |
| Sus_newGene_91813 | 2.98E-05 | 1.521 | up | ENSSSCG00000010894 | 1.05E-05 | 1.521 | up |
| ENSSSCG00000000661 | 2.32E-05 | 1.527 | up | ENSSSCG00000010483 | 4.99E-05 | 1.522 | up |
| ENSSSCG00000003154 | 9.53E-06 | 1.528 | up | ENSSSCG00000017882 | 8.91E-06 | 1.528 | up |
| ENSSSCG00000027348 | 1.36E-05 | 1.529 | up | Sus_newGene_94172 | 1.12E-02 | 1.529 | up |
| Sus_newGene_186678 | 1.55E-05 | 1.530 | up | ENSSSCG00000024587 | 6.72E-05 | 1.529 | up |
| ENSSSCG00000003429 | 1.39E-05 | 1.534 | up | ENSSSCG00000006718 | 1.13E-05 | 1.533 | up |
| ENSSSCG00000034351 | 8.27E-06 | 1.535 | up | ENSSSCG00000013457 | 7.91E-06 | 1.534 | up |
| ENSSSCG00000015619 | 9.50E-05 | 1.537 | up | ENSSSCG00000009364 | 9.74E-06 | 1.535 | up |
| ENSSSCG00000016201 | 7.44E-06 | 1.539 | up | Sus_newGene_31156 | 1.17E-05 | 1.537 | up |
| ENSSSCG00000007874 | 1.02E-05 | 1.542 | up | Sus_newGene_36839 | 6.54E-05 | 1.542 | up |
| Sus_newGene_32963 | 9.36E-03 | 1.543 | up | ENSSSCG00000011136 | 6.62E-06 | 1.543 | up |
| ENSSSCG00000026063 | 6.67E-06 | 1.547 | up | ENSSSCG00000039321 | 1.30E-05 | 1.547 | up |
| ENSSSCG00000032941 | 5.55E-06 | 1.552 | up | ENSSSCG00000015846 | 5.79E-06 | 1.550 | up |
| ENSSSCG00000017177 | 9.45E-05 | 1.553 | up | ENSSSCG00000025729 | 5.52E-06 | 1.552 | up |
| ENSSSCG00000011127 | 1.84E-05 | 1.553 | up | ENSSSCG00000010849 | 6.51E-06 | 1.553 | up |
| ENSSSCG00000017069 | 6.85E-06 | 1.557 | up | ENSSSCG00000014627 | 5.23E-06 | 1.555 | up |
| ENSSSCG00000006383 | 1.06E-04 | 1.560 | up | ENSSSCG00000001573 | 4.87E-06 | 1.559 | up |
| ENSSSCG00000011485 | 6.42E-06 | 1.562 | up | ENSSSCG00000006063 | 1.01E-05 | 1.561 | up |
| ENSSSCG00000008340 | 6.91E-06 | 1.566 | up | ENSSSCG00000011168 | 6.51E-05 | 1.566 | up |
| ENSSSCG00000015748 | 1.37E-04 | 1.570 | up | Sus_newGene_105499 | 4.27E-06 | 1.568 | up |
| ENSSSCG00000000555 | 4.03E-06 | 1.572 | up | ENSSSCG00000032229 | 1.97E-04 | 1.571 | up |
| ENSSSCG00000014570 | 3.45E-03 | 1.573 | up | ENSSSCG00000029570 | 4.38E-06 | 1.573 | up |
| ENSSSCG00000027725 | 1.16E-05 | 1.574 | up | ENSSSCG00000035596 | 1.84E-05 | 1.574 | up |
| ENSSSCG00000005589 | 2.22E-05 | 1.575 | up | ENSSSCG00000011251 | 3.55E-06 | 1.575 | up |
| Sus_newGene_80331 | 2.97E-04 | 1.577 | up | ENSSSCG00000010322 | 8.64E-06 | 1.577 | up |
| ENSSSCG00000020657 | 3.33E-06 | 1.579 | up | ENSSSCG00000003414 | 6.29E-04 | 1.578 | up |
| ENSSSCG00000025374 | 2.90E-06 | 1.586 | up | ENSSSCG00000009037 | 3.03E-06 | 1.583 | up |
| Sus_newGene_114995 | 2.03E-03 | 1.588 | up | ENSSSCG00000008180 | 3.24E-06 | 1.587 | up |
| ENSSSCG00000027812 | 2.95E-06 | 1.590 | up | ENSSSCG00000017748 | 5.76E-06 | 1.588 | up |
| ENSSSCG00000006521 | 4.27E-06 | 1.591 | up | ENSSSCG00000013742 | 6.04E-05 | 1.591 | up |
| ENSSSCG00000012695 | 2.89E-06 | 1.592 | up | ENSSSCG00000016191 | 5.88E-06 | 1.592 | up |
| ENSSSCG00000037958 | 2.31E-06 | 1.597 | up | ENSSSCG00000007991 | 1.79E-04 | 1.596 | up |
| Sus_newGene_120780 | 9.21E-06 | 1.598 | up | Sus_newGene_131444 | 2.96E-06 | 1.598 | up |
| Sus_newGene_13102 | 2.31E-06 | 1.599 | up | ENSSSCG00000005322 | 2.24E-06 | 1.599 | up |
| ENSSSCG00000011610 | 3.05E-06 | 1.600 | up | ENSSSCG00000036569 | 4.51E-03 | 1.600 | up |
| ENSSSCG00000003192 | 3.23E-06 | 1.601 | up | ENSSSCG00000038801 | 2.66E-06 | 1.600 | up |
| ENSSSCG00000012983 | 2.08E-06 | 1.604 | up | ENSSSCG00000035849 | 2.62E-06 | 1.601 | up |
| ENSSSCG00000006121 | 3.93E-02 | 1.604 | up | ENSSSCG00000040533 | 1.46E-03 | 1.604 | up |
| ENSSSCG00000015135 | 2.13E-06 | 1.606 | up | ENSSSCG00000039134 | 1.17E-04 | 1.604 | up |
| ENSSSCG00000021326 | 1.57E-04 | 1.607 | up | ENSSSCG00000037910 | 1.99E-06 | 1.606 | up |
| ENSSSCG00000002957 | 5.83E-03 | 1.613 | up | ENSSSCG00000010359 | 4.19E-04 | 1.610 | up |
| ENSSSCG00000028109 | 2.52E-06 | 1.615 | up | ENSSSCG00000030428 | 1.72E-06 | 1.614 | up |
| ENSSSCG00000001499 | 6.72E-06 | 1.618 | up | Sus_newGene_106572 | 1.58E-06 | 1.617 | up |
| ENSSSCG00000032860 | 7.92E-04 | 1.619 | up | ENSSSCG00000036076 | 2.10E-03 | 1.618 | up |
| ENSSSCG00000017753 | 2.44E-06 | 1.620 | up | Sus_newGene_193634 | 4.02E-06 | 1.620 | up |
| ENSSSCG00000003941 | 1.49E-06 | 1.622 | up | ENSSSCG00000011120 | 1.42E-06 | 1.622 | up |
| ENSSSCG00000010706 | 1.59E-06 | 1.623 | up | ENSSSCG00000011391 | 2.08E-05 | 1.622 | up |
| ENSSSCG00000009338 | 1.31E-06 | 1.627 | up | ENSSSCG00000000753 | 2.09E-06 | 1.624 | up |
| ENSSSCG00000007815 | 1.35E-06 | 1.629 | up | ENSSSCG00000006115 | 1.82E-06 | 1.629 | up |
| ENSSSCG00000006247 | 2.88E-05 | 1.633 | up | ENSSSCG00000034868 | 3.05E-05 | 1.631 | up |
| ENSSSCG00000015136 | 1.19E-05 | 1.636 | up | ENSSSCG00000003738 | 3.01E-06 | 1.635 | up |
| ENSSSCG00000040739 | 3.32E-02 | 1.638 | up | ENSSSCG00000006562 | 1.07E-06 | 1.637 | up |
| ENSSSCG00000003395 | 1.74E-05 | 1.639 | up | Sus_newGene_169085 | 2.88E-06 | 1.638 | up |
| Sus_newGene_181397 | 1.80E-06 | 1.640 | up | ENSSSCG00000033089 | 1.74E-05 | 1.639 | up |
| ENSSSCG00000014056 | 1.04E-05 | 1.644 | up | ENSSSCG00000035181 | 1.44E-06 | 1.642 | up |
| ENSSSCG00000008127 | 1.23E-06 | 1.645 | up | ENSSSCG00000015880 | 1.34E-06 | 1.644 | up |
| ENSSSCG00000009742 | 8.68E-07 | 1.646 | up | ENSSSCG00000022194 | 2.59E-06 | 1.646 | up |
| ENSSSCG00000007980 | 7.77E-07 | 1.655 | up | ENSSSCG00000026224 | 1.07E-06 | 1.646 | up |
| ENSSSCG00000033819 | 5.79E-05 | 1.656 | up | ENSSSCG00000003253 | 7.44E-07 | 1.656 | up |
| ENSSSCG00000002436 | 6.97E-07 | 1.658 | up | ENSSSCG00000002921 | 8.64E-07 | 1.657 | up |
| ENSSSCG00000014293 | 2.60E-05 | 1.659 | up | ENSSSCG00000001869 | 1.22E-06 | 1.658 | up |
| Sus_newGene_54700 | 3.59E-06 | 1.662 | up | ENSSSCG00000025561 | 1.01E-06 | 1.660 | up |
| ENSSSCG00000013538 | 6.26E-07 | 1.665 | up | ENSSSCG00000030497 | 7.41E-07 | 1.662 | up |
| Sus_newGene_185747 | 1.27E-05 | 1.669 | up | ENSSSCG00000037002 | 1.12E-06 | 1.668 | up |
| ENSSSCG00000009794 | 5.22E-07 | 1.672 | up | Sus_newGene_181613 | 9.33E-06 | 1.671 | up |
| ENSSSCG00000029901 | 5.39E-07 | 1.673 | up | ENSSSCG00000008589 | 5.40E-06 | 1.672 | up |
| ENSSSCG00000034444 | 6.48E-07 | 1.677 | up | Sus_newGene_167486 | 7.61E-04 | 1.673 | up |
| Sus_newGene_181614 | 8.89E-04 | 1.679 | up | ENSSSCG00000017479 | 5.42E-07 | 1.679 | up |
| ENSSSCG00000027224 | 4.25E-07 | 1.681 | up | ENSSSCG00000000205 | 3.36E-03 | 1.679 | up |
| ENSSSCG00000004199 | 5.39E-07 | 1.683 | up | ENSSSCG00000015780 | 1.16E-06 | 1.683 | up |
| ENSSSCG00000011074 | 5.36E-07 | 1.684 | up | ENSSSCG00000034767 | 1.02E-06 | 1.684 | up |
| Sus_newGene_35372 | 1.45E-06 | 1.685 | up | ENSSSCG00000029456 | 3.94E-07 | 1.685 | up |
| ENSSSCG00000017578 | 4.19E-07 | 1.686 | up | ENSSSCG00000038929 | 3.56E-06 | 1.686 | up |
| ENSSSCG00000035544 | 3.43E-07 | 1.691 | up | ENSSSCG00000007529 | 4.75E-04 | 1.689 | up |
| ENSSSCG00000012027 | 4.13E-07 | 1.702 | up | ENSSSCG00000030623 | 3.24E-07 | 1.696 | up |
| ENSSSCG00000005317 | 1.21E-06 | 1.704 | up | Sus_newGene_169102 | 7.06E-07 | 1.703 | up |
| ENSSSCG00000005711 | 2.58E-07 | 1.705 | up | ENSSSCG00000034153 | 1.28E-05 | 1.705 | up |
| ENSSSCG00000016842 | 2.92E-07 | 1.708 | up | ENSSSCG00000034854 | 2.48E-07 | 1.707 | up |
| Sus_newGene_167439 | 2.97E-05 | 1.709 | up | ENSSSCG00000037539 | 2.25E-03 | 1.709 | up |
| Sus_newGene_162049 | 6.47E-07 | 1.712 | up | ENSSSCG00000011264 | 1.06E-06 | 1.710 | up |
| ENSSSCG00000039442 | 6.16E-07 | 1.714 | up | ENSSSCG00000028612 | 2.73E-07 | 1.712 | up |
| ENSSSCG00000007278 | 1.83E-07 | 1.723 | up | ENSSSCG00000039770 | 2.54E-07 | 1.714 | up |
| ENSSSCG00000014387 | 2.96E-07 | 1.727 | up | ENSSSCG00000016625 | 5.03E-07 | 1.726 | up |
| ENSSSCG00000034357 | 3.80E-07 | 1.727 | up | Sus_newGene_4316 | 5.75E-03 | 1.727 | up |
| ENSSSCG00000017401 | 1.58E-07 | 1.729 | up | ENSSSCG00000038950 | 1.62E-07 | 1.728 | up |
| Sus_newGene_82191 | 1.97E-06 | 1.730 | up | ENSSSCG00000003030 | 1.70E-07 | 1.729 | up |
| ENSSSCG00000002298 | 1.80E-07 | 1.732 | up | ENSSSCG00000012985 | 1.56E-07 | 1.731 | up |
| Sus_newGene_144623 | 4.54E-07 | 1.735 | up | ENSSSCG00000026276 | 1.43E-07 | 1.733 | up |
| ENSSSCG00000035527 | 2.41E-07 | 1.736 | up | ENSSSCG00000015283 | 1.67E-07 | 1.735 | up |
| ENSSSCG00000013764 | 1.78E-06 | 1.737 | up | ENSSSCG00000026686 | 6.97E-06 | 1.737 | up |
| ENSSSCG00000006698 | 1.45E-07 | 1.740 | up | ENSSSCG00000016520 | 1.61E-07 | 1.739 | up |
| ENSSSCG00000017983 | 4.72E-07 | 1.741 | up | Sus_newGene_205587 | 2.81E-04 | 1.740 | up |
| ENSSSCG00000009648 | 7.63E-03 | 1.741 | up | ENSSSCG00000012766 | 1.51E-07 | 1.741 | up |
| ENSSSCG00000005030 | 1.16E-07 | 1.749 | up | Sus_newGene_169108 | 2.43E-06 | 1.748 | up |
| Sus_newGene_35375 | 2.68E-05 | 1.750 | up | ENSSSCG00000001765 | 1.04E-07 | 1.750 | up |
| ENSSSCG00000016971 | 9.44E-08 | 1.755 | up | ENSSSCG00000037262 | 2.29E-05 | 1.751 | up |
| Sus_newGene_116478 | 6.10E-07 | 1.757 | up | ENSSSCG00000000567 | 1.02E-06 | 1.756 | up |
| ENSSSCG00000015543 | 4.80E-07 | 1.757 | up | ENSSSCG00000011178 | 5.92E-04 | 1.757 | up |
| Sus_newGene_190124 | 7.85E-03 | 1.759 | up | ENSSSCG00000026498 | 8.59E-08 | 1.757 | up |
| ENSSSCG00000015692 | 7.77E-08 | 1.762 | up | ENSSSCG00000014112 | 9.24E-08 | 1.761 | up |
| ENSSSCG00000017300 | 1.29E-07 | 1.765 | up | ENSSSCG00000004037 | 9.00E-08 | 1.764 | up |
| ENSSSCG00000020801 | 1.24E-07 | 1.768 | up | ENSSSCG00000002836 | 9.09E-06 | 1.768 | up |
| Sus_newGene_157022 | 4.16E-04 | 1.770 | up | Sus_newGene_171742 | 6.95E-04 | 1.768 | up |
| ENSSSCG00000018039 | 6.71E-08 | 1.772 | up | ENSSSCG00000021023 | 7.05E-08 | 1.772 | up |
| ENSSSCG00000032024 | 6.57E-08 | 1.774 | up | ENSSSCG00000007473 | 6.04E-08 | 1.774 | up |
| ENSSSCG00000010746 | 1.59E-07 | 1.781 | up | Sus_newGene_51181 | 8.83E-06 | 1.780 | up |
| ENSSSCG00000026116 | 5.12E-07 | 1.782 | up | ENSSSCG00000007249 | 7.01E-08 | 1.781 | up |
| ENSSSCG00000009230 | 6.20E-08 | 1.787 | up | ENSSSCG00000017230 | 3.96E-07 | 1.783 | up |
| ENSSSCG00000007782 | 3.78E-07 | 1.788 | up | ENSSSCG00000032137 | 2.11E-06 | 1.787 | up |
| ENSSSCG00000031592 | 3.58E-08 | 1.801 | up | ENSSSCG00000029960 | 8.14E-04 | 1.795 | up |
| Sus_newGene_167709 | 1.49E-07 | 1.806 | up | ENSSSCG00000037115 | 3.88E-06 | 1.803 | up |
| ENSSSCG00000011075 | 2.86E-08 | 1.808 | up | ENSSSCG00000009801 | 1.37E-07 | 1.808 | up |
| ENSSSCG00000010579 | 2.81E-08 | 1.812 | up | ENSSSCG00000008337 | 3.00E-08 | 1.809 | up |
| ENSSSCG00000001905 | 2.61E-06 | 1.814 | up | ENSSSCG00000006289 | 9.44E-07 | 1.813 | up |
| ENSSSCG00000017569 | 8.54E-03 | 1.819 | up | ENSSSCG00000012104 | 1.51E-06 | 1.816 | up |
| ENSSSCG00000001646 | 1.05E-07 | 1.820 | up | Sus_newGene_80529 | 1.33E-06 | 1.820 | up |
| ENSSSCG00000016125 | 1.91E-08 | 1.826 | up | ENSSSCG00000026152 | 2.67E-07 | 1.822 | up |
| ENSSSCG00000005667 | 1.88E-08 | 1.828 | up | ENSSSCG00000020663 | 1.91E-08 | 1.827 | up |
| ENSSSCG00000014833 | 1.45E-04 | 1.829 | up | ENSSSCG00000005250 | 4.40E-07 | 1.828 | up |
| Sus_newGene_162046 | 2.30E-07 | 1.831 | up | ENSSSCG00000031818 | 2.23E-06 | 1.831 | up |
| ENSSSCG00000011795 | 2.97E-03 | 1.834 | up | ENSSSCG00000016725 | 1.70E-08 | 1.831 | up |
| ENSSSCG00000002857 | 6.58E-03 | 1.840 | up | ENSSSCG00000027378 | 2.36E-08 | 1.839 | up |
| ENSSSCG00000033907 | 1.08E-07 | 1.842 | up | ENSSSCG00000036155 | 2.18E-08 | 1.841 | up |
| ENSSSCG00000006571 | 1.39E-08 | 1.847 | up | ENSSSCG00000024794 | 1.26E-08 | 1.845 | up |
| Sus_newGene_142433 | 1.48E-08 | 1.857 | up | ENSSSCG00000015238 | 1.38E-08 | 1.849 | up |
| ENSSSCG00000006940 | 1.23E-08 | 1.861 | up | ENSSSCG00000028922 | 3.86E-03 | 1.857 | up |
| ENSSSCG00000002494 | 2.61E-06 | 1.862 | up | ENSSSCG00000004577 | 1.92E-08 | 1.862 | up |
| ENSSSCG00000037046 | 8.51E-09 | 1.866 | up | ENSSSCG00000004332 | 6.41E-07 | 1.863 | up |
| ENSSSCG00000011643 | 7.76E-09 | 1.870 | up | ENSSSCG00000004067 | 1.29E-04 | 1.869 | up |
| ENSSSCG00000030291 | 7.17E-09 | 1.874 | up | Sus_newGene_183413 | 2.33E-03 | 1.873 | up |
| ENSSSCG00000040313 | 1.05E-08 | 1.882 | up | Sus_newGene_108420 | 9.03E-07 | 1.879 | up |
| ENSSSCG00000016956 | 5.53E-09 | 1.883 | up | ENSSSCG00000008443 | 5.49E-09 | 1.882 | up |
| Sus_newGene_139921 | 4.01E-08 | 1.887 | up | Sus_newGene_165057 | 5.38E-06 | 1.887 | up |
| ENSSSCG00000007324 | 8.89E-09 | 1.893 | up | ENSSSCG00000025349 | 9.95E-09 | 1.891 | up |
| ENSSSCG00000022957 | 4.05E-09 | 1.895 | up | ENSSSCG00000015480 | 5.53E-09 | 1.895 | up |
| ENSSSCG00000028277 | 1.18E-07 | 1.898 | up | ENSSSCG00000011384 | 1.21E-06 | 1.897 | up |
| ENSSSCG00000032957 | 4.54E-09 | 1.898 | up | ENSSSCG00000028978 | 3.82E-09 | 1.898 | up |
| ENSSSCG00000035062 | 5.61E-09 | 1.899 | up | Sus_newGene_167898 | 2.74E-04 | 1.898 | up |
| ENSSSCG00000024596 | 3.64E-08 | 1.902 | up | ENSSSCG00000005291 | 4.63E-09 | 1.900 | up |
| Sus_newGene_142148 | 3.69E-05 | 1.905 | up | Sus_newGene_204322 | 7.32E-05 | 1.905 | up |
| ENSSSCG00000007155 | 2.93E-09 | 1.911 | up | ENSSSCG00000014399 | 2.08E-08 | 1.909 | up |
| Sus_newGene_102847 | 1.80E-03 | 1.915 | up | Sus_newGene_93568 | 4.70E-05 | 1.911 | up |
| ENSSSCG00000036772 | 6.71E-08 | 1.915 | up | ENSSSCG00000028759 | 2.73E-09 | 1.915 | up |
| ENSSSCG00000012328 | 4.42E-09 | 1.919 | up | Sus_newGene_114008 | 4.72E-06 | 1.917 | up |
| ENSSSCG00000025537 | 7.35E-09 | 1.925 | up | ENSSSCG00000010002 | 2.39E-09 | 1.924 | up |
| ENSSSCG00000023593 | 2.00E-09 | 1.927 | up | ENSSSCG00000010219 | 3.34E-09 | 1.926 | up |
| ENSSSCG00000039688 | 1.10E-03 | 1.928 | up | ENSSSCG00000031337 | 2.63E-09 | 1.927 | up |
| ENSSSCG00000024157 | 1.92E-09 | 1.928 | up | ENSSSCG00000007000 | 5.13E-09 | 1.928 | up |
| Sus_newGene_56192 | 2.12E-04 | 1.930 | up | ENSSSCG00000040315 | 2.28E-06 | 1.929 | up |
| ENSSSCG00000026113 | 2.10E-09 | 1.934 | up | ENSSSCG00000030424 | 4.38E-09 | 1.932 | up |
| Sus_newGene_86107 | 1.24E-04 | 1.936 | up | ENSSSCG00000013860 | 3.56E-09 | 1.935 | up |
| ENSSSCG00000022462 | 1.50E-06 | 1.936 | up | ENSSSCG00000031180 | 2.00E-06 | 1.936 | up |
| ENSSSCG00000033762 | 5.04E-07 | 1.943 | up | Sus_newGene_49959 | 5.54E-09 | 1.938 | up |
| Sus_newGene_82182 | 2.37E-09 | 1.948 | up | Sus_newGene_75688 | 1.62E-09 | 1.945 | up |
| ENSSSCG00000002777 | 2.99E-03 | 1.953 | up | Sus_newGene_182263 | 2.03E-04 | 1.950 | up |
| ENSSSCG00000006970 | 3.43E-09 | 1.958 | up | ENSSSCG00000023121 | 2.42E-07 | 1.957 | up |
| ENSSSCG00000035859 | 4.63E-07 | 1.963 | up | ENSSSCG00000025943 | 3.52E-07 | 1.958 | up |
| ENSSSCG00000011246 | 1.93E-04 | 1.970 | up | ENSSSCG00000036537 | 8.26E-10 | 1.964 | up |
| ENSSSCG00000010915 | 6.75E-10 | 1.973 | up | ENSSSCG00000004980 | 1.35E-09 | 1.970 | up |
| ENSSSCG00000031719 | 6.72E-10 | 1.978 | up | ENSSSCG00000014894 | 2.31E-04 | 1.976 | up |
| ENSSSCG00000013021 | 5.52E-10 | 1.982 | up | ENSSSCG00000013884 | 1.50E-09 | 1.982 | up |
| ENSSSCG00000012126 | 5.35E-10 | 1.983 | up | ENSSSCG00000000263 | 1.80E-09 | 1.983 | up |
| ENSSSCG00000038164 | 3.30E-08 | 1.985 | up | ENSSSCG00000027998 | 3.02E-05 | 1.984 | up |
| ENSSSCG00000009761 | 6.28E-10 | 1.988 | up | Sus_newGene_76346 | 6.66E-10 | 1.985 | up |
| ENSSSCG00000029815 | 4.71E-10 | 1.991 | up | Sus_newGene_190824 | 2.22E-05 | 1.991 | up |
| ENSSSCG00000029249 | 4.09E-10 | 1.994 | up | ENSSSCG00000015774 | 4.23E-10 | 1.993 | up |
| ENSSSCG00000032668 | 2.41E-08 | 2.000 | up | Sus_newGene_142152 | 9.71E-07 | 1.998 | up |
| ENSSSCG00000037318 | 1.38E-06 | 2.005 | up | Sus_newGene_56592 | 6.07E-10 | 2.001 | up |
| ENSSSCG00000036877 | 7.50E-10 | 2.006 | up | Sus_newGene_184245 | 3.08E-10 | 2.006 | up |
| ENSSSCG00000040581 | 3.66E-07 | 2.007 | up | ENSSSCG00000036145 | 3.66E-10 | 2.006 | up |
| ENSSSCG00000014110 | 4.16E-10 | 2.008 | up | ENSSSCG00000012035 | 4.26E-10 | 2.008 | up |
| Sus_newGene_143436 | 2.96E-10 | 2.010 | up | ENSSSCG00000037621 | 3.70E-10 | 2.009 | up |
| ENSSSCG00000017817 | 3.45E-10 | 2.012 | up | Sus_newGene_552 | 1.08E-08 | 2.011 | up |
| Sus_newGene_54123 | 2.59E-10 | 2.013 | up | ENSSSCG00000001900 | 5.87E-10 | 2.013 | up |
| ENSSSCG00000003756 | 5.86E-05 | 2.014 | up | ENSSSCG00000013915 | 2.31E-05 | 2.014 | up |
| ENSSSCG00000040578 | 6.16E-10 | 2.017 | up | ENSSSCG00000027669 | 5.17E-10 | 2.015 | up |
| ENSSSCG00000033528 | 2.26E-10 | 2.021 | up | ENSSSCG00000030767 | 2.23E-09 | 2.017 | up |
| ENSSSCG00000032786 | 5.84E-08 | 2.025 | up | ENSSSCG00000035267 | 3.35E-08 | 2.024 | up |
| ENSSSCG00000002509 | 8.34E-10 | 2.032 | up | Sus_newGene_53943 | 2.74E-10 | 2.026 | up |
| ENSSSCG00000004835 | 6.27E-09 | 2.035 | up | ENSSSCG00000007248 | 2.04E-10 | 2.033 | up |
| ENSSSCG00000014567 | 1.03E-08 | 2.038 | up | Sus_newGene_95071 | 1.58E-09 | 2.036 | up |
| ENSSSCG00000012769 | 1.27E-03 | 2.046 | up | Sus_newGene_194402 | 1.75E-05 | 2.044 | up |
| ENSSSCG00000024009 | 2.37E-04 | 2.057 | up | Sus_newGene_82195 | 9.55E-06 | 2.054 | up |
| ENSSSCG00000000029 | 2.16E-08 | 2.059 | up | ENSSSCG00000017962 | 2.64E-08 | 2.058 | up |
| Sus_newGene_141147 | 1.72E-07 | 2.067 | up | ENSSSCG00000002372 | 8.63E-11 | 2.059 | up |
| Sus_newGene_36841 | 3.33E-08 | 2.070 | up | ENSSSCG00000004149 | 2.61E-09 | 2.067 | up |
| ENSSSCG00000002368 | 1.12E-10 | 2.079 | up | ENSSSCG00000036135 | 6.18E-11 | 2.073 | up |
| ENSSSCG00000023483 | 8.02E-11 | 2.083 | up | ENSSSCG00000017251 | 4.91E-08 | 2.081 | up |
| ENSSSCG00000038071 | 1.46E-05 | 2.084 | up | ENSSSCG00000021562 | 5.14E-11 | 2.084 | up |
| ENSSSCG00000009885 | 5.64E-11 | 2.086 | up | Sus_newGene_432 | 1.05E-10 | 2.085 | up |
| ENSSSCG00000007284 | 4.38E-11 | 2.094 | up | ENSSSCG00000025252 | 1.08E-10 | 2.089 | up |
| ENSSSCG00000008835 | 3.73E-11 | 2.096 | up | Sus_newGene_106568 | 3.88E-11 | 2.094 | up |
| Sus_newGene_120955 | 1.81E-07 | 2.106 | up | ENSSSCG00000007067 | 3.61E-11 | 2.104 | up |
| ENSSSCG00000040339 | 1.11E-04 | 2.107 | up | ENSSSCG00000016568 | 1.28E-10 | 2.106 | up |
| ENSSSCG00000009408 | 2.86E-11 | 2.110 | up | Sus_newGene_153495 | 4.23E-11 | 2.108 | up |
| ENSSSCG00000037545 | 3.20E-11 | 2.117 | up | ENSSSCG00000016513 | 1.50E-10 | 2.114 | up |
| ENSSSCG00000022302 | 1.33E-04 | 2.120 | up | ENSSSCG00000022099 | 1.96E-11 | 2.119 | up |
| ENSSSCG00000033299 | 1.22E-10 | 2.122 | up | ENSSSCG00000005654 | 2.90E-11 | 2.121 | up |
| ENSSSCG00000013400 | 3.08E-11 | 2.124 | up | ENSSSCG00000003709 | 2.32E-10 | 2.123 | up |
| ENSSSCG00000013351 | 1.75E-11 | 2.130 | up | ENSSSCG00000023498 | 1.12E-06 | 2.129 | up |
| ENSSSCG00000036052 | 4.24E-11 | 2.132 | up | ENSSSCG00000005036 | 8.73E-06 | 2.130 | up |
| ENSSSCG00000005751 | 1.82E-11 | 2.134 | up | ENSSSCG00000040738 | 3.77E-11 | 2.132 | up |
| ENSSSCG00000022506 | 8.01E-09 | 2.135 | up | ENSSSCG00000011859 | 1.65E-11 | 2.134 | up |
| ENSSSCG00000036049 | 2.33E-11 | 2.144 | up | Sus_newGene_82425 | 5.14E-04 | 2.142 | up |
| ENSSSCG00000031317 | 9.75E-10 | 2.148 | up | ENSSSCG00000000191 | 1.10E-11 | 2.147 | up |
| ENSSSCG00000003559 | 9.00E-12 | 2.152 | up | ENSSSCG00000024396 | 1.11E-10 | 2.149 | up |
| ENSSSCG00000007951 | 9.21E-12 | 2.154 | up | ENSSSCG00000014259 | 7.95E-05 | 2.154 | up |
| ENSSSCG00000010211 | 7.32E-12 | 2.160 | up | ENSSSCG00000014176 | 8.41E-12 | 2.160 | up |
| ENSSSCG00000016974 | 9.70E-12 | 2.166 | up | ENSSSCG00000001409 | 6.35E-12 | 2.163 | up |
| ENSSSCG00000010502 | 8.72E-11 | 2.170 | up | ENSSSCG00000011129 | 3.36E-09 | 2.168 | up |
| Sus_newGene_37135 | 4.11E-05 | 2.172 | up | Sus_newGene_82187 | 3.95E-11 | 2.170 | up |
| ENSSSCG00000035774 | 5.11E-12 | 2.172 | up | ENSSSCG00000009953 | 1.68E-07 | 2.172 | up |
| ENSSSCG00000009053 | 2.26E-10 | 2.180 | up | ENSSSCG00000030345 | 3.99E-11 | 2.179 | up |
| ENSSSCG00000015074 | 4.74E-12 | 2.184 | up | ENSSSCG00000033760 | 2.89E-04 | 2.182 | up |
| ENSSSCG00000008984 | 3.35E-12 | 2.189 | up | ENSSSCG00000003921 | 3.21E-07 | 2.186 | up |
| ENSSSCG00000025176 | 8.10E-12 | 2.190 | up | ENSSSCG00000012741 | 6.87E-10 | 2.190 | up |
| ENSSSCG00000023371 | 3.00E-12 | 2.196 | up | ENSSSCG00000004338 | 8.56E-11 | 2.195 | up |
| ENSSSCG00000009786 | 1.75E-11 | 2.203 | up | Sus_newGene_124737 | 2.17E-04 | 2.202 | up |
| ENSSSCG00000009545 | 4.54E-12 | 2.205 | up | ENSSSCG00000003577 | 2.28E-12 | 2.204 | up |
| ENSSSCG00000037450 | 1.36E-05 | 2.217 | up | ENSSSCG00000000075 | 2.44E-12 | 2.208 | up |
| ENSSSCG00000032446 | 1.02E-04 | 2.221 | up | ENSSSCG00000012656 | 1.50E-10 | 2.220 | up |
| ENSSSCG00000029260 | 3.97E-06 | 2.229 | up | ENSSSCG00000028530 | 1.63E-04 | 2.221 | up |
| ENSSSCG00000013248 | 1.05E-12 | 2.235 | up | ENSSSCG00000005617 | 3.44E-12 | 2.231 | up |
| ENSSSCG00000017146 | 1.07E-12 | 2.241 | up | ENSSSCG00000017728 | 1.38E-12 | 2.236 | up |
| ENSSSCG00000025214 | 9.54E-10 | 2.252 | up | ENSSSCG00000015584 | 1.77E-07 | 2.251 | up |
| ENSSSCG00000004603 | 6.92E-13 | 2.255 | up | ENSSSCG00000011259 | 2.18E-07 | 2.252 | up |
| ENSSSCG00000002689 | 5.12E-12 | 2.259 | up | ENSSSCG00000034105 | 6.07E-13 | 2.255 | up |
| ENSSSCG00000004961 | 3.53E-13 | 2.276 | up | ENSSSCG00000016119 | 5.78E-13 | 2.275 | up |
| ENSSSCG00000039580 | 3.82E-13 | 2.283 | up | ENSSSCG00000039198 | 1.42E-11 | 2.280 | up |
| Sus_newGene_141153 | 3.00E-07 | 2.289 | up | ENSSSCG00000022204 | 5.88E-13 | 2.288 | up |
| ENSSSCG00000032261 | 5.30E-13 | 2.290 | up | ENSSSCG00000009806 | 6.58E-10 | 2.290 | up |
| ENSSSCG00000014256 | 3.80E-10 | 2.293 | up | ENSSSCG00000004044 | 3.14E-13 | 2.292 | up |
| ENSSSCG00000013263 | 7.73E-13 | 2.299 | up | ENSSSCG00000016578 | 2.21E-13 | 2.295 | up |
| ENSSSCG00000034677 | 4.32E-13 | 2.301 | up | ENSSSCG00000032749 | 1.83E-13 | 2.301 | up |
| ENSSSCG00000012241 | 5.04E-13 | 2.308 | up | Sus_newGene_37097 | 2.19E-12 | 2.305 | up |
| ENSSSCG00000007243 | 1.11E-11 | 2.314 | up | ENSSSCG00000000274 | 1.65E-13 | 2.308 | up |
| ENSSSCG00000000092 | 9.13E-08 | 2.324 | up | ENSSSCG00000015401 | 1.32E-05 | 2.321 | up |
| ENSSSCG00000008292 | 3.01E-11 | 2.326 | up | ENSSSCG00000030196 | 1.75E-13 | 2.325 | up |
| ENSSSCG00000003451 | 6.92E-14 | 2.337 | up | Sus_newGene_13082 | 8.76E-14 | 2.328 | up |
| ENSSSCG00000007606 | 5.47E-14 | 2.347 | up | ENSSSCG00000038144 | 1.17E-11 | 2.341 | up |
| ENSSSCG00000040731 | 5.03E-14 | 2.349 | up | ENSSSCG00000013078 | 1.39E-13 | 2.347 | up |
| ENSSSCG00000030048 | 2.11E-13 | 2.358 | up | Sus_newGene_126931 | 1.99E-13 | 2.355 | up |
| Sus_newGene_129168 | 6.91E-14 | 2.369 | up | Sus_newGene_82184 | 2.89E-11 | 2.361 | up |
| ENSSSCG00000012679 | 2.82E-14 | 2.373 | up | Sus_newGene_54694 | 1.01E-06 | 2.369 | up |
| ENSSSCG00000003569 | 8.14E-14 | 2.383 | up | ENSSSCG00000006688 | 4.60E-12 | 2.378 | up |
| ENSSSCG00000031327 | 2.33E-07 | 2.388 | up | Sus_newGene_185767 | 6.72E-11 | 2.387 | up |
| Sus_newGene_82179 | 1.71E-14 | 2.395 | up | ENSSSCG00000034484 | 1.67E-14 | 2.390 | up |
| ENSSSCG00000003488 | 1.60E-14 | 2.406 | up | Sus_newGene_162022 | 9.68E-06 | 2.402 | up |
| ENSSSCG00000012324 | 1.58E-12 | 2.412 | up | ENSSSCG00000000386 | 1.14E-14 | 2.407 | up |
| ENSSSCG00000015786 | 6.82E-13 | 2.421 | up | ENSSSCG00000002950 | 4.77E-14 | 2.418 | up |
| Sus_newGene_111793 | 6.00E-15 | 2.429 | up | ENSSSCG00000012655 | 2.31E-12 | 2.428 | up |
| ENSSSCG00000001920 | 1.33E-10 | 2.438 | up | ENSSSCG00000016461 | 5.88E-15 | 2.435 | up |
| Sus_newGene_42481 | 6.93E-07 | 2.439 | up | Sus_newGene_49963 | 2.09E-10 | 2.438 | up |
| Sus_newGene_82144 | 8.44E-15 | 2.441 | up | ENSSSCG00000039926 | 8.99E-15 | 2.439 | up |
| ENSSSCG00000034853 | 4.82E-14 | 2.452 | up | ENSSSCG00000032452 | 3.55E-15 | 2.446 | up |
| ENSSSCG00000040697 | 6.77E-15 | 2.475 | up | Sus_newGene_177919 | 6.50E-08 | 2.455 | up |
| Sus_newGene_418 | 1.33E-15 | 2.480 | up | ENSSSCG00000008357 | 1.51E-13 | 2.477 | up |
| ENSSSCG00000011681 | 6.99E-15 | 2.484 | up | ENSSSCG00000031488 | 1.67E-15 | 2.481 | up |
| Sus_newGene_4314 | 1.61E-07 | 2.494 | up | Sus_newGene_13095 | 1.59E-08 | 2.491 | up |
| Sus_newGene_33598 | 5.16E-13 | 2.504 | up | ENSSSCG00000040403 | 2.69E-10 | 2.502 | up |
| Sus_newGene_82188 | 6.66E-16 | 2.507 | up | ENSSSCG00000008241 | 2.24E-12 | 2.505 | up |
| ENSSSCG00000013545 | 1.34E-11 | 2.519 | up | ENSSSCG00000032610 | 1.50E-11 | 2.514 | up |
| ENSSSCG00000037893 | 3.27E-05 | 2.534 | up | ENSSSCG00000032718 | 7.38E-06 | 2.533 | up |
| ENSSSCG00000032761 | 2.22E-16 | 2.548 | up | ENSSSCG00000002752 | 3.22E-15 | 2.536 | up |
| ENSSSCG00000004549 | 1.11E-05 | 2.553 | up | ENSSSCG00000029944 | 2.22E-16 | 2.550 | up |
| ENSSSCG00000012832 | 1.11E-16 | 2.564 | up | ENSSSCG00000022705 | 4.06E-14 | 2.560 | up |
| ENSSSCG00000027558 | 1.13E-06 | 2.565 | up | ENSSSCG00000007705 | 2.35E-14 | 2.565 | up |
| ENSSSCG00000026733 | 1.11E-16 | 2.583 | up | ENSSSCG00000027312 | 8.77E-15 | 2.571 | up |
| Sus_newGene_126933 | 9.99E-16 | 2.585 | up | ENSSSCG00000016685 | 1.92E-14 | 2.583 | up |
| Sus_newGene_17667 | 7.29E-14 | 2.601 | up | ENSSSCG00000016754 | 0.00E+00 | 2.595 | up |
| Sus_newGene_41109 | 1.11E-16 | 2.606 | up | ENSSSCG00000016529 | 0.00E+00 | 2.605 | up |
| ENSSSCG00000023200 | 4.92E-07 | 2.612 | up | ENSSSCG00000039171 | 0.00E+00 | 2.607 | up |
| ENSSSCG00000034360 | 3.33E-16 | 2.614 | up | ENSSSCG00000003238 | 7.83E-08 | 2.613 | up |
| ENSSSCG00000016606 | 1.11E-10 | 2.621 | up | ENSSSCG00000038684 | 8.90E-06 | 2.616 | up |
| ENSSSCG00000033765 | 6.66E-07 | 2.633 | up | Sus_newGene_31862 | 4.88E-15 | 2.626 | up |
| ENSSSCG00000025270 | 7.70E-08 | 2.645 | up | ENSSSCG00000026412 | 0.00E+00 | 2.642 | up |
| ENSSSCG00000000278 | 0.00E+00 | 2.646 | up | Sus_newGene_167441 | 1.11E-16 | 2.645 | up |
| ENSSSCG00000007789 | 0.00E+00 | 2.650 | up | ENSSSCG00000032383 | 2.22E-16 | 2.648 | up |
| Sus_newGene_101586 | 3.04E-11 | 2.679 | up | ENSSSCG00000021657 | 9.73E-06 | 2.666 | up |
| ENSSSCG00000034319 | 5.20E-14 | 2.691 | up | ENSSSCG00000033626 | 0.00E+00 | 2.681 | up |
| Sus_newGene_189038 | 1.11E-16 | 2.703 | up | ENSSSCG00000000531 | 0.00E+00 | 2.700 | up |
| ENSSSCG00000031361 | 6.15E-14 | 2.705 | up | ENSSSCG00000012793 | 0.00E+00 | 2.704 | up |
| ENSSSCG00000000431 | 1.37E-12 | 2.713 | up | ENSSSCG00000010033 | 0.00E+00 | 2.710 | up |
| ENSSSCG00000023181 | 1.69E-08 | 2.725 | up | ENSSSCG00000006651 | 2.84E-07 | 2.723 | up |
| Sus_newGene_53157 | 2.06E-08 | 2.748 | up | Sus_newGene_124117 | 0.00E+00 | 2.727 | up |
| ENSSSCG00000029189 | 0.00E+00 | 2.752 | up | ENSSSCG00000032803 | 3.89E-10 | 2.750 | up |
| ENSSSCG00000025141 | 7.06E-08 | 2.758 | up | ENSSSCG00000027987 | 1.59E-07 | 2.756 | up |
| ENSSSCG00000028018 | 0.00E+00 | 2.763 | up | ENSSSCG00000001362 | 0.00E+00 | 2.762 | up |
| ENSSSCG00000017333 | 1.20E-07 | 2.771 | up | ENSSSCG00000016830 | 4.33E-15 | 2.769 | up |
| ENSSSCG00000030680 | 7.66E-13 | 2.777 | up | ENSSSCG00000010638 | 1.11E-16 | 2.774 | up |
| ENSSSCG00000000068 | 0.00E+00 | 2.805 | up | ENSSSCG00000012479 | 3.77E-06 | 2.786 | up |
| Sus_newGene_191676 | 7.44E-15 | 2.822 | up | ENSSSCG00000001049 | 0.00E+00 | 2.815 | up |
| ENSSSCG00000004368 | 0.00E+00 | 2.826 | up | ENSSSCG00000004404 | 1.29E-09 | 2.822 | up |
| ENSSSCG00000036768 | 0.00E+00 | 2.830 | up | Sus_newGene_21140 | 1.91E-06 | 2.827 | up |
| ENSSSCG00000021845 | 0.00E+00 | 2.856 | up | Sus_newGene_167433 | 0.00E+00 | 2.837 | up |
| ENSSSCG00000017211 | 0.00E+00 | 2.876 | up | ENSSSCG00000017428 | 0.00E+00 | 2.861 | up |
| ENSSSCG00000004192 | 0.00E+00 | 2.885 | up | ENSSSCG00000031384 | 3.12E-09 | 2.880 | up |
| ENSSSCG00000023324 | 0.00E+00 | 2.923 | up | ENSSSCG00000008192 | 0.00E+00 | 2.886 | up |
| Sus_newGene_99070 | 7.56E-08 | 2.950 | up | Sus_newGene_192592 | 1.99E-11 | 2.934 | up |
| ENSSSCG00000012724 | 1.84E-07 | 2.963 | up | ENSSSCG00000001639 | 2.02E-14 | 2.959 | up |
| ENSSSCG00000023080 | 0.00E+00 | 2.995 | up | ENSSSCG00000004215 | 0.00E+00 | 2.973 | up |
| ENSSSCG00000040144 | 2.55E-15 | 3.040 | up | ENSSSCG00000008618 | 5.36E-11 | 3.005 | up |
| Sus_newGene_170941 | 6.45E-09 | 3.081 | up | ENSSSCG00000017262 | 1.37E-11 | 3.067 | up |
| ENSSSCG00000017506 | 0.00E+00 | 3.108 | up | Sus_newGene_25614 | 7.77E-16 | 3.099 | up |
| Sus_newGene_96960 | 1.02E-08 | 3.121 | up | ENSSSCG00000040937 | 1.11E-16 | 3.110 | up |
| Sus_newGene_203733 | 1.95E-09 | 3.143 | up | ENSSSCG00000032422 | 0.00E+00 | 3.126 | up |
| ENSSSCG00000040682 | 0.00E+00 | 3.155 | up | Sus_newGene_182265 | 0.00E+00 | 3.153 | up |
| Sus_newGene_82202 | 5.43E-08 | 3.181 | up | Sus_newGene_167444 | 1.03E-10 | 3.177 | up |
| ENSSSCG00000023304 | 0.00E+00 | 3.211 | up | ENSSSCG00000039310 | 1.98E-07 | 3.200 | up |
| Sus_newGene_205593 | 9.49E-08 | 3.242 | up | ENSSSCG00000028278 | 0.00E+00 | 3.223 | up |
| Sus_newGene_18813 | 0.00E+00 | 3.293 | up | ENSSSCG00000002774 | 0.00E+00 | 3.257 | up |
| ENSSSCG00000022031 | 6.67E-10 | 3.332 | up | ENSSSCG00000000423 | 0.00E+00 | 3.310 | up |
| ENSSSCG00000036976 | 9.99E-16 | 3.430 | up | ENSSSCG00000035101 | 0.00E+00 | 3.333 | up |
| ENSSSCG00000015271 | 0.00E+00 | 3.435 | up | ENSSSCG00000017095 | 0.00E+00 | 3.433 | up |
| Sus_newGene_2277 | 0.00E+00 | 3.474 | up | ENSSSCG00000031321 | 0.00E+00 | 3.440 | up |
| ENSSSCG00000028814 | 0.00E+00 | 3.497 | up | ENSSSCG00000026349 | 9.77E-10 | 3.488 | up |
| ENSSSCG00000003514 | 0.00E+00 | 3.523 | up | Sus_newGene_33600 | 1.11E-15 | 3.501 | up |
| ENSSSCG00000039224 | 0.00E+00 | 3.623 | up | Sus_newGene_152741 | 8.95E-09 | 3.607 | up |
| ENSSSCG00000000436 | 1.73E-13 | 3.644 | up | ENSSSCG00000036322 | 0.00E+00 | 3.637 | up |
| Sus_newGene_31675 | 2.76E-14 | 3.656 | up | Sus_newGene_53274 | 0.00E+00 | 3.651 | up |
| ENSSSCG00000011404 | 0.00E+00 | 3.688 | up | ENSSSCG00000034008 | 2.86E-09 | 3.669 | up |
| ENSSSCG00000017516 | 9.95E-12 | 3.713 | up | ENSSSCG00000001716 | 0.00E+00 | 3.710 | up |
| ENSSSCG00000035493 | 0.00E+00 | 3.812 | up | ENSSSCG00000035598 | 0.00E+00 | 3.748 | up |
| ENSSSCG00000010503 | 0.00E+00 | 3.823 | up | ENSSSCG00000022250 | 6.66E-16 | 3.816 | up |
| ENSSSCG00000021155 | 5.18E-11 | 3.874 | up | ENSSSCG00000033570 | 4.53E-14 | 3.860 | up |
| ENSSSCG00000033606 | 8.66E-15 | 3.930 | up | ENSSSCG00000011310 | 0.00E+00 | 3.915 | up |
| ENSSSCG00000014380 | 0.00E+00 | 3.987 | up | ENSSSCG00000032291 | 8.66E-15 | 3.930 | up |
| ENSSSCG00000037206 | 0.00E+00 | 4.033 | up | Sus_newGene_142146 | 1.26E-10 | 3.987 | up |
| Sus_newGene_93656 | 4.72E-10 | 4.115 | up | ENSSSCG00000007181 | 8.62E-13 | 4.069 | up |
| ENSSSCG00000035313 | 0.00E+00 | 4.197 | up | Sus_newGene_30249 | 9.58E-11 | 4.197 | up |
| ENSSSCG00000037748 | 0.00E+00 | 4.252 | up | ENSSSCG00000033978 | 1.22E-15 | 4.224 | up |
| Sus_newGene_124927 | 2.22E-16 | 4.293 | up | ENSSSCG00000035838 | 2.02E-11 | 4.275 | up |
| ENSSSCG00000038838 | 6.48E-12 | 4.331 | up | Sus_newGene_112094 | 9.45E-12 | 4.313 | up |
| ENSSSCG00000038662 | 0.00E+00 | 4.413 | up | Sus_newGene_42485 | 4.46E-12 | 4.349 | up |
| ENSSSCG00000029954 | 0.00E+00 | 4.619 | up | ENSSSCG00000038970 | 0.00E+00 | 4.576 | up |
| Sus_newGene_41444 | 1.55E-15 | 4.714 | up | ENSSSCG00000032480 | 7.55E-15 | 4.642 | up |
| Sus_newGene_153535 | 0.00E+00 | 4.923 | up | ENSSSCG00000003555 | 0.00E+00 | 4.746 | up |
| ENSSSCG00000001537 | 0.00E+00 | 4.964 | up | ENSSSCG00000032605 | 0.00E+00 | 4.929 | up |
| Sus_newGene_171367 | 0.00E+00 | 4.982 | up | Sus_newGene_37813 | 0.00E+00 | 4.970 | up |
| ENSSSCG00000035428 | 0.00E+00 | 5.083 | up | ENSSSCG00000008004 | 0.00E+00 | 5.039 | up |
| ENSSSCG00000013931 | 0.00E+00 | 5.195 | up | Sus_newGene_96522 | 0.00E+00 | 5.094 | up |
| Sus_newGene_151598 | 0.00E+00 | 5.359 | up | ENSSSCG00000020710 | 0.00E+00 | 5.266 | up |
| Sus_newGene_95715 | 0.00E+00 | 5.848 | up | ENSSSCG00000007312 | 0.00E+00 | 5.376 | up |
| ENSSSCG00000021899 | 9.95E-21 | 6.100 | up | Sus_newGene_76434 | 2.23E-20 | 5.899 | up |
| ENSSSCG00000017551 | 9.25E-25 | 6.268 | up | ENSSSCG00000005915 | 1.01E-23 | 6.183 | up |
| ENSSSCG00000029474 | 6.88E-28 | 7.132 | up | Sus_newGene_79274 | 5.23E-26 | 6.665 | up |
| ENSSSCG00000032395 | 7.74E-31 | 7.797 | up | ENSSSCG00000040186 | 7.23E-30 | 7.731 | up |
| ENSSSCG00000027057 | 3.23E-56 | 9.906 | up | Sus_newGene_46815 | 2.25E-35 | 8.131 | up |

|Log_2_(Fold change)| ≥ 1 and adjusted FDR < 0.05 was the cut-off criteria for DEmRNAs.

**Supplementary Table S3. Differentially expressed miRNAs in pGCs treated with H_2_O_2_.**

| **miRNA ID** | **Mature sequence (5'-3')** | **FDR** | **log2FC** | **Regulation** |
| --- | --- | --- | --- | --- |
| novel-miR-418 | GCCGUGGAGACCUGGGCC | 1.10E-06 | -7.8585 | down |
| ssc-miR-411 | GGGCCUGUGGCUCAGAGGG | 7.21E-29 | -7.2456 | down |
| novel-miR-119 | GCCUUGAAGACUUUGGCA | 4.56E-03 | -6.7587 | down |
| novel-miR-309 | AUGGUGAGUGUGGACGUG | 4.56E-03 | -6.7587 | down |
| ssc-miR-198 | UAGUGGCUAGGAUUCGGCG | 3.17E-13 | -3.6064 | down |
| ssc-miR-370 | GCCUGCUGGGGUGGAACCUGGU | 8.29E-03 | -3.1780 | down |
| novel-miR-90 | UGGUUUGUUUGGGUUUGUU | 5.62E-14 | -2.9383 | down |
| ssc-miR-369 | AGUGGGCUGAGGAUCUGGCGUUGU | 4.70E-02 | -2.8181 | down |
| novel-miR-430 | GUUAACGAAUCUGACUAGG | 3.29E-03 | -2.3368 | down |
| novel-miR-176 | AGACCUUGAUGGCUGGCUGAGUCUC | 2.82E-05 | -2.1535 | down |
| ssc-miR-451 | AAACCGUUACCAUUACUGAGUU | 3.25E-03 | -1.6654 | down |
| novel-miR-74 | GAUAUGAGAGUGUUGGUCCUGA | 6.65E-03 | -1.5484 | down |
| ssc-miR-365-5p | GAGGGACUUUCAGGGGCAGCUGU | 9.44E-03 | -1.4185 | down |
| ssc-miR-424-3p | CAAAACGUGAGGCGCUGCUAU | 3.21E-02 | -1.3667 | down |
| novel-miR-112 | GAUGUGGCUCGGAUCUGGUGUUGC | 3.26E-05 | -1.2628 | down |
| ssc-miR-221-5p | ACCUGGCAUACAAUGUAGAUUUCUGU | 4.49E-06 | -1.1509 | down |
| ssc-miR-27b-5p | AGAGCUUAGCUGAUUGGUGAACA | 1.61E-05 | -1.1040 | down |
| ssc-miR-130a | CAGUGCAAUGUUAAAAGGGCAU | 2.53E-03 | 1.0160 | up |
| ssc-miR-30b-5p | UGUAAACAUCCUACACUCAGCU | 2.82E-05 | 1.0280 | up |
| ssc-miR-23 | UAAUUUUAUGUAUAAGCUAGU | 1.83E-04 | 1.1110 | up |
| ssc-miR-1249 | ACGCCCUUCCCCCCCUUCUUCA | 8.82E-06 | 1.1524 | up |
| ssc-miR-497 | CAGCAGCACACUGUGGUUUGU | 1.62E-05 | 1.1873 | up |
| ssc-miR-210 | CUGUGCGUGUGACAGCGGCUGA | 3.29E-08 | 1.3152 | up |
| novel-miR-11 | GGACUUCCCGUGUGGCUCACAAC | 3.33E-10 | 1.5672 | up |
| ssc-miR-192 | AGACUCUUAGAGGUGGAU | 1.28E-05 | 1.6194 | up |
| novel-miR-318 | UGGGCUGUGGUCCUUGCCUCUUU | 5.21E-03 | 1.6806 | up |
| novel-miR-370 | UGUCAUGCUGGGGAGUGUAGUG | 2.57E-02 | 1.7543 | up |
| novel-miR-121 | GCCUCCUCCUCCCCCCCUU | 4.15E-03 | 1.7563 | up |
| novel-miR-259 | CAUCCCUUGCAUGGUGGAGGG | 3.48E-04 | 1.7864 | up |
| novel-miR-244 | CCUCCCCCCCCUCCCCGGC | 9.79E-03 | 1.8153 | up |
| ssc-miR-17-3p | ACUGCAGUGAAGGCACUUGUAG | 9.76E-03 | 1.9925 | up |
| ssc-miR-125 | GGACUUCCCGUGUGGCUCAC | 5.61E-10 | 2.0518 | up |
| ssc-miR-373 | UGACUCUUAGAGGUGGAU | 1.99E-04 | 2.0589 | up |
| novel-miR-104 | GGACUUCCCGUGUGGCUCACAAC | 2.64E-14 | 2.0643 | up |
| ssc-miR-413 | ACUGGACUUGGAAUCAGAAGGU | 3.81E-02 | 2.1262 | up |
| novel-miR-407 | GGACUUCCCGUGUGGCUCAC | 1.05E-09 | 2.2680 | up |
| ssc-miR-110 | GCAUGUGGGCUAGUUUCAAACAAGG | 1.67E-02 | 2.3364 | up |
| ssc-miR-141 | UCCCAGCUGGUCAUUAAUCCUC | 4.48E-04 | 2.3696 | up |
| ssc-miR-126-5p | CAUUAUUACUUUUGGUACGCG | 1.18E-03 | 2.3907 | up |
| novel-miR-111 | CUCAUGGAUCUCCUCUCCCAGU | 1.09E-05 | 2.5071 | up |
| ssc-miR-213 | UGUGAGGGCGAUCUGGCU | 5.66E-06 | 2.5585 | up |
| ssc-miR-182 | GUGGGGAAGAACUACAAGACAGCU | 2.52E-12 | 2.6334 | up |
| ssc-miR-1285 | CUGGGCAACAUAGCGAGACCCCGU | 3.89E-15 | 2.6607 | up |
| novel-miR-340 | AGGUGCGGCCACUUGUUU | 9.64E-20 | 2.6846 | up |
| novel-miR-287 | AGGUGCGGCCACUUGUUU | 9.64E-20 | 2.8732 | up |
| novel-miR-194 | GUAUGUGAGCGGGGGGCUGGUGGG | 3.93E-02 | 3.3469 | up |
| ssc-miR-142 | CUCCCAGCGGUGCCUCCU | 5.58E-03 | 3.7581 | up |
| ssc-miR-193a-3p | AACUGGCCUACAAAGUCCCAGU | 3.53E-03 | 3.8449 | up |
| novel-miR-417 | GUGGCUGAGGUGAGAACA | 1.04E-08 | 5.2102 | up |
| novel-miR-85 | AGGGAGGGUUUGGGUUCAUCUGU | 4.00E-02 | 6.1469 | up |
| novel-miR-69 | UCUCCAGCCAGACCAGAGGAU | 4.00E-02 | 6.1469 | up |
| novel-miR-285 | UCUCUCCCCCUCCGUCCCAGG | 2.28E-02 | 6.2969 | up |
| ssc-miR-128 | CGGGGCGGCAGGCUGAGCCU | 1.34E-02 | 6.4327 | up |
| novel-miR-228 | GCGGGACUGUGCAACUUGCUUUGAC | 8.03E-03 | 6.5568 | up |
| novel-miR-336 | UCCCUGGCCUGGGAACUUUU | 5.40E-04 | 7.0548 | up |

|Log_2_(Fold change)| ≥ 1 and adjusted FDR < 0.05 was the cut-off criteria for DEmiRNAs.

**Supplementary Table S4. GO enrichment analysis of DEmRNAs after H_2_O_2_ treatment**

| **Category** | **KEGG pathway terms** | **Count** | **Percentage %** | ***P*-value^1^** |
| --- | --- | --- | --- | --- |
| GOTERM_BP | GO:0000122 negative regulation of transcription from RNA polymerase II promoter | 46 | 3.619 | 4.08E-06 |
| GOTERM_BP | GO:0045944 positive regulation of transcription from RNA polymerase II promoter | 57 | 4.485 | 7.93E-06 |
| GOTERM_BP | GO:0007507 heart development | 19 | 1.495 | 5.44E-05 |
| GOTERM_BP | GO:0036342 post-anal tail morphogenesis | 7 | 0.551 | 1.35E-04 |
| GOTERM_BP | GO:0060070 canonical Wnt signaling pathway | 14 | 1.101 | 1.89E-04 |
| GOTERM_BP | GO:0030036 actin cytoskeleton organization | 15 | 1.180 | 1.96E-04 |
| GOTERM_BP | GO:0045599 negative regulation of fat cell differentiation | 9 | 0.708 | 5.17E-04 |
| GOTERM_BP | GO:0045893 positive regulation of transcription, DNA-templated | 28 | 2.203 | 6.06E-04 |
| GOTERM_BP | GO:0006357 regulation of transcription from RNA polymerase II promoter | 28 | 2.203 | 6.57E-04 |
| GOTERM_BP | GO:0042733 embryonic digit morphogenesis | 10 | 0.787 | 6.88E-04 |
| GOTERM_BP | GO:0001649 osteoblast differentiation | 13 | 1.023 | 7.17E-04 |
| GOTERM_BP | GO:0045662 negative regulation of myoblast differentiation | 7 | 0.551 | 1.08E-03 |
| GOTERM_BP | GO:0006351 transcription, DNA-templated | 36 | 2.832 | 1.23E-03 |
| GOTERM_BP | GO:0030048 actin filament-based movement | 5 | 0.393 | 1.37E-03 |
| GOTERM_BP | GO:0043536 positive regulation of blood vessel endothelial cell migration | 5 | 0.393 | 1.37E-03 |
| GOTERM_BP | GO:0060021 palate development | 11 | 0.865 | 1.62E-03 |
| GOTERM_BP | GO:0045669 positive regulation of osteoblast differentiation | 9 | 0.708 | 1.69E-03 |
| GOTERM_BP | GO:0006974 cellular response to DNA damage stimulus | 13 | 1.023 | 1.75E-03 |
| GOTERM_BP | GO:0030324 lung development | 10 | 0.787 | 1.86E-03 |
| GOTERM_BP | GO:0000288 nuclear-transcribed mRNA catabolic process, deadenylation | 5 | 0.393 | 2.32E-03 |
| GOTERM_BP | GO:0045444 fat cell differentiation | 10 | 0.787 | 2.64E-03 |
| GOTERM_BP | GO:0042752 regulation of circadian rhythm | 8 | 0.629 | 2.70E-03 |
| GOTERM_BP | GO:0090090 negative regulation of canonical Wnt signaling pathway | 11 | 0.865 | 3.03E-03 |
| GOTERM_BP | GO:0061036 positive regulation of cartilage development | 5 | 0.393 | 3.66E-03 |
| GOTERM_BP | GO:0009791 post-embryonic development | 10 | 0.787 | 4.96E-03 |
| GOTERM_BP | GO:0006355 regulation of transcription, DNA-templated | 41 | 3.226 | 5.37E-03 |
| GOTERM_BP | GO:0035019 somatic stem cell population maintenance | 7 | 0.551 | 5.42E-03 |
| GOTERM_BP | GO:0035307 positive regulation of protein dephosphorylation | 5 | 0.393 | 5.43E-03 |
| GOTERM_BP | GO:0051056 regulation of small GTPase mediated signal transduction | 5 | 0.393 | 5.43E-03 |
| GOTERM_BP | GO:0001843 neural tube closure | 10 | 0.787 | 6.60E-03 |
| GOTERM_BP | GO:0043966 histone H3 acetylation | 7 | 0.551 | 6.71E-03 |
| GOTERM_BP | GO:0045773 positive regulation of axon extension | 5 | 0.393 | 7.70E-03 |
| GOTERM_BP | GO:0043407 negative regulation of MAP kinase activity | 7 | 0.551 | 8.22E-03 |
| GOTERM_BP | GO:0050680 negative regulation of epithelial cell proliferation | 8 | 0.629 | 8.50E-03 |
| GOTERM_BP | GO:0050770 regulation of axonogenesis | 5 | 0.393 | 1.05E-02 |
| GOTERM_BP | GO:0045732 positive regulation of protein catabolic process | 7 | 0.551 | 1.19E-02 |
| GOTERM_BP | GO:0043123 positive regulation of I-kappaB kinase/NF-kappaB signaling | 14 | 1.101 | 1.22E-02 |
| GOTERM_BP | GO:0042981 regulation of apoptotic process | 16 | 1.259 | 1.29E-02 |
| GOTERM_BP | GO:0021915 neural tube development | 5 | 0.393 | 1.39E-02 |
| GOTERM_BP | GO:0006338 chromatin remodeling | 9 | 0.708 | 1.41E-02 |
| GOTERM_BP | GO:0007160 cell-matrix adhesion | 9 | 0.708 | 1.41E-02 |
| GOTERM_BP | GO:0007050 cell cycle arrest | 9 | 0.708 | 1.41E-02 |
| GOTERM_BP | GO:0060028 convergent extension involved in axis elongation | 3 | 0.236 | 1.42E-02 |
| GOTERM_BP | GO:0007296 vitellogenesis | 3 | 0.236 | 1.42E-02 |
| GOTERM_BP | GO:0007059 chromosome segregation | 8 | 0.629 | 1.56E-02 |
| GOTERM_BP | GO:0035329 hippo signaling | 5 | 0.393 | 1.79E-02 |
| GOTERM_BP | GO:0002053 positive regulation of mesenchymal cell proliferation | 5 | 0.393 | 1.79E-02 |
| GOTERM_BP | GO:0003281 ventricular septum development | 5 | 0.393 | 1.79E-02 |
| GOTERM_BP | GO:0043473 pigmentation | 5 | 0.393 | 1.79E-02 |
| GOTERM_BP | GO:0000165 MAPK cascade | 7 | 0.551 | 1.95E-02 |
| GOTERM_BP | GO:0043507 positive regulation of JUN kinase activity | 6 | 0.472 | 2.00E-02 |
| GOTERM_BP | GO:0097150 neuronal stem cell population maintenance | 4 | 0.315 | 2.13E-02 |
| GOTERM_BP | GO:0046328 regulation of JNK cascade | 4 | 0.315 | 2.13E-02 |
| GOTERM_BP | GO:0090179 planar cell polarity pathway involved in neural tube closure | 4 | 0.315 | 2.13E-02 |
| GOTERM_BP | GO:0001701 in utero embryonic development | 16 | 1.259 | 2.30E-02 |
| GOTERM_BP | GO:0031175 neuron projection development | 9 | 0.708 | 2.53E-02 |
| GOTERM_BP | GO:0016477 cell migration | 13 | 1.023 | 2.58E-02 |
| GOTERM_BP | GO:0001822 kidney development | 7 | 0.551 | 2.60E-02 |
| GOTERM_BP | GO:0007044 cell-substrate junction assembly | 3 | 0.236 | 2.71E-02 |
| GOTERM_BP | GO:0043535 regulation of blood vessel endothelial cell migration | 3 | 0.236 | 2.71E-02 |
| GOTERM_BP | GO:0006929 substrate-dependent cell migration | 3 | 0.236 | 2.71E-02 |
| GOTERM_BP | GO:0033962 cytoplasmic mRNA processing body assembly | 4 | 0.315 | 2.89E-02 |
| GOTERM_BP | GO:0016525 negative regulation of angiogenesis | 7 | 0.551 | 2.98E-02 |
| GOTERM_BP | GO:0035914 skeletal muscle cell differentiation | 6 | 0.472 | 3.29E-02 |
| GOTERM_BP | GO:0002244 hematopoietic progenitor cell differentiation | 8 | 0.629 | 3.30E-02 |
| GOTERM_BP | GO:0030866 cortical actin cytoskeleton organization | 5 | 0.393 | 3.40E-02 |
| GOTERM_BP | GO:0043066 negative regulation of apoptotic process | 22 | 1.731 | 3.60E-02 |
| GOTERM_BP | GO:0030509 BMP signaling pathway | 8 | 0.629 | 3.67E-02 |
| GOTERM_BP | GO:0001837 epithelial to mesenchymal transition | 4 | 0.315 | 3.77E-02 |
| GOTERM_BP | GO:0001666 response to hypoxia | 9 | 0.708 | 3.79E-02 |
| GOTERM_BP | GO:0007173 epidermal growth factor receptor signaling pathway | 6 | 0.472 | 3.81E-02 |
| GOTERM_BP | GO:0032092 positive regulation of protein binding | 7 | 0.551 | 3.83E-02 |
| GOTERM_BP | GO:0009749 response to glucose | 5 | 0.393 | 4.07E-02 |
| GOTERM_BP | GO:0030198 extracellular matrix organization | 8 | 0.629 | 4.08E-02 |
| GOTERM_BP | GO:0032873 negative regulation of stress-activated MAPK cascade | 3 | 0.236 | 4.31E-02 |
| GOTERM_BP | GO:0051023 regulation of immunoglobulin secretion | 3 | 0.236 | 4.31E-02 |
| GOTERM_BP | GO:0032793 positive regulation of CREB transcription factor activity | 3 | 0.236 | 4.31E-02 |
| GOTERM_BP | GO:0051533 positive regulation of NFAT protein import into nucleus | 3 | 0.236 | 4.31E-02 |
| GOTERM_BP | GO:1904938 planar cell polarity pathway involved in axon guidance | 3 | 0.236 | 4.31E-02 |
| GOTERM_BP | GO:0033601 positive regulation of mammary gland epithelial cell proliferation | 3 | 0.236 | 4.31E-02 |
| GOTERM_BP | GO:0038031 non-canonical Wnt signaling pathway via JNK cascade | 3 | 0.236 | 4.31E-02 |
| GOTERM_BP | GO:0001938 positive regulation of endothelial cell proliferation | 7 | 0.551 | 4.31E-02 |
| GOTERM_BP | GO:0034446 substrate adhesion-dependent cell spreading | 6 | 0.472 | 4.37E-02 |
| GOTERM_BP | GO:0034613 cellular protein localization | 6 | 0.472 | 4.37E-02 |
| GOTERM_BP | GO:0030154 cell differentiation | 15 | 1.180 | 4.55E-02 |
| GOTERM_BP | GO:0001702 gastrulation with mouth forming second | 4 | 0.315 | 4.76E-02 |
| GOTERM_BP | GO:0003007 heart morphogenesis | 4 | 0.315 | 4.76E-02 |
| GOTERM_BP | GO:0035264 multicellular organism growth | 9 | 0.708 | 4.96E-02 |
| GOTERM_BP | GO:0030512 negative regulation of TGF-beta receptor signaling pathway | 6 | 0.472 | 4.99E-02 |
| GOTERM_CC | GO:0005654 nucleoplasm | 112 | 8.812 | 1.99E-09 |
| GOTERM_CC | GO:0005634 nucleus | 203 | 15.972 | 1.08E-07 |
| GOTERM_CC | GO:0005737 cytoplasm | 205 | 16.129 | 2.45E-07 |
| GOTERM_CC | GO:0005802 trans-Golgi network | 17 | 1.338 | 1.07E-04 |
| GOTERM_CC | GO:0005925 focal adhesion | 31 | 2.439 | 6.11E-04 |
| GOTERM_CC | GO:0005578 proteinaceous extracellular matrix | 21 | 1.652 | 1.70E-03 |
| GOTERM_CC | GO:0009897 external side of plasma membrane | 17 | 1.338 | 8.03E-03 |
| GOTERM_CC | GO:0016328 lateral plasma membrane | 7 | 0.551 | 8.85E-03 |
| GOTERM_CC | GO:0005829 cytosol | 67 | 5.271 | 9.25E-03 |
| GOTERM_CC | GO:0043231 intracellular membrane-bounded organelle | 15 | 1.180 | 1.08E-02 |
| GOTERM_CC | GO:0070776 MOZ/MORF histone acetyltransferase complex | 3 | 0.236 | 1.35E-02 |
| GOTERM_CC | GO:0001726 ruffle | 8 | 0.629 | 1.38E-02 |
| GOTERM_CC | GO:0030027 lamellipodium | 12 | 0.944 | 1.58E-02 |
| GOTERM_CC | GO:0048471 perinuclear region of cytoplasm | 28 | 2.203 | 1.75E-02 |
| GOTERM_CC | GO:0005765 lysosomal membrane | 15 | 1.180 | 1.99E-02 |
| GOTERM_CC | GO:0035102 PRC1 complex | 4 | 0.315 | 2.70E-02 |
| GOTERM_CC | GO:0005876 spindle microtubule | 6 | 0.472 | 2.99E-02 |
| GOTERM_CC | GO:0005856 cytoskeleton | 12 | 0.944 | 3.09E-02 |
| GOTERM_CC | GO:0005911 cell-cell junction | 13 | 1.023 | 3.38E-02 |
| GOTERM_CC | GO:0070062 extracellular exosome | 128 | 10.071 | 3.71E-02 |
| GOTERM_CC | GO:0035631 CD40 receptor complex | 3 | 0.236 | 4.11E-02 |
| GOTERM_CC | GO:0005874 microtubule | 12 | 0.944 | 4.13E-02 |
| GOTERM_CC | GO:0005770 late endosome | 9 | 0.708 | 4.39E-02 |
| GOTERM_CC | GO:0031012 extracellular matrix | 12 | 0.944 | 4.43E-02 |
| GOTERM_CC | GO:0000932 cytoplasmic mRNA processing body | 8 | 0.629 | 4.45E-02 |
| GOTERM_MF | GO:0008270 zinc ion binding | 84 | 6.609 | 4.90E-05 |
| GOTERM_MF | GO:0003713 transcription coactivator activity | 19 | 1.495 | 8.62E-05 |
| GOTERM_MF | GO:0005096 GTPase activator activity | 21 | 1.652 | 2.96E-04 |
| GOTERM_MF | GO:0000978 RNA polymerase II core promoter -specific DNA binding | 27 | 2.124 | 7.76E-04 |
| GOTERM_MF | GO:0050840 extracellular matrix binding | 7 | 0.551 | 9.09E-04 |
| GOTERM_MF | GO:0003714 transcription corepressor activity | 15 | 1.180 | 1.22E-03 |
| GOTERM_MF | GO:0046872 metal ion binding | 76 | 5.980 | 1.65E-03 |
| GOTERM_MF | GO:0005509 calcium ion binding | 50 | 3.934 | 2.09E-03 |
| GOTERM_MF | GO:0003682 chromatin binding | 29 | 2.282 | 2.47E-03 |
| GOTERM_MF | GO:0008134 transcription factor binding | 9 | 0.708 | 3.57E-03 |
| GOTERM_MF | GO:0044212 transcription regulatory region DNA binding | 15 | 1.180 | 3.70E-03 |
| GOTERM_MF | GO:0003700 transcription factor activity, sequence-specific DNA binding | 43 | 3.383 | 4.50E-03 |
| GOTERM_MF | GO:0004402 histone acetyltransferase activity | 7 | 0.551 | 5.75E-03 |
| GOTERM_MF | GO:0000981 RNA polymerase II transcription factor activit-specific DNA binding | 17 | 1.338 | 1.67E-02 |
| GOTERM_MF | GO:0004674 protein serine/threonine kinase activity | 21 | 1.652 | 2.02E-02 |
| GOTERM_MF | GO:0043565 sequence-specific DNA binding | 29 | 2.282 | 2.02E-02 |
| GOTERM_MF | GO:0008201 heparin binding | 12 | 0.944 | 2.32E-02 |
| GOTERM_MF | GO:0019902 phosphatase binding | 3 | 0.236 | 2.55E-02 |
| GOTERM_MF | GO:0044822 poly(A) RNA binding | 58 | 4.563 | 3.68E-02 |
| GOTERM_MF | GO:0005201 extracellular matrix structural constituent | 6 | 0.472 | 3.89E-02 |
| GOTERM_MF | GO:0003677 DNA binding | 43 | 3.383 | 4.51E-02 |

^1^GO terms with *P*-value < 0.05 were considered as significant functional processes of DEmRNAs.

**Supplementary Table S5. KEGG pathway analysis of DEmRNAs after H_2_O_2_ treatment**

| **KEGG pathway terms** | **Count** | ***P*-value^1^** | **Percentage %** |
| --- | --- | --- | --- |
| ssc05200:Pathways in cancer | 52 | 1.46E-07 | 4.091266719 |
| ssc04151:PI3K-Akt signaling pathway | 44 | 1.80E-06 | 3.46184107 |
| ssc05166:HTLV-I infection | 36 | 3.76E-06 | 2.832415421 |
| ssc04910:Insulin signaling pathway | 24 | 4.99E-06 | 1.888276947 |
| ssc04152:AMPK signaling pathway | 21 | 3.11E-05 | 1.652242329 |
| ssc04390:Hippo signaling pathway | 23 | 3.45E-05 | 1.809598741 |
| ssc04510:Focal adhesion | 28 | 5.79E-05 | 2.202989772 |
| ssc04310:Wnt signaling pathway | 21 | 9.22E-05 | 1.652242329 |
| ssc04512:ECM-receptor interaction | 16 | 1.20E-04 | 1.258851298 |
| ssc04919:Thyroid hormone signaling pathway | 19 | 1.96E-04 | 1.494885917 |
| ssc04024:cAMP signaling pathway | 26 | 5.47E-04 | 2.04563336 |
| ssc04550:Signaling pathways regulating pluripotency of stem cells | 19 | 1.11E-03 | 1.494885917 |
| ssc04931:Insulin resistance | 17 | 1.48E-03 | 1.337529504 |
| ssc04810:Regulation of actin cytoskeleton | 24 | 2.42E-03 | 1.888276947 |
| ssc04520:Adherens junction | 12 | 4.20E-03 | 0.944138474 |
| ssc04015:Rap1 signaling pathway | 24 | 4.55E-03 | 1.888276947 |
| ssc04916:Melanogenesis | 14 | 4.84E-03 | 1.101494886 |
| ssc05202:Transcriptional misregulation in cancer | 19 | 6.29E-03 | 1.494885917 |
| ssc04350:TGF-beta signaling pathway | 12 | 7.84E-03 | 0.944138474 |
| ssc04068:FoxO signaling pathway | 17 | 8.37E-03 | 1.337529504 |
| ssc04022:cGMP-PKG signaling pathway | 19 | 9.32E-03 | 1.494885917 |
| ssc05213:Endometrial cancer | 9 | 1.01E-02 | 0.708103855 |
| ssc05132:Salmonella infection | 12 | 1.14E-02 | 0.944138474 |
| ssc04360:Axon guidance | 15 | 1.58E-02 | 1.180173092 |
| ssc05222:Small cell lung cancer | 12 | 1.60E-02 | 0.944138474 |
| ssc04668:TNF signaling pathway | 14 | 1.65E-02 | 1.101494886 |
| ssc05210:Colorectal cancer | 10 | 2.45E-02 | 0.786782061 |
| ssc04722:Neurotrophin signaling pathway | 14 | 3.18E-02 | 1.101494886 |
| ssc04340:Hedgehog signaling pathway | 5 | 3.27E-02 | 0.393391031 |
| ssc05133:Pertussis | 10 | 3.68E-02 | 0.786782061 |
| ssc05412:Arrhythmogenic right ventricular cardiomyopathy | 9 | 3.77E-02 | 0.708103855 |
| ssc05215:Prostate cancer | 11 | 3.80E-02 | 0.865460268 |
| ssc05144:Malaria | 8 | 4.13E-02 | 0.629425649 |
| ssc04530:Tight junction | 11 | 4.36E-02 | 0.865460268 |
| ssc05211:Renal cell carcinoma | 9 | 4.44E-02 | 0.708103855 |
| ssc05100:Bacterial invasion of epithelial cells | 10 | 4.60E-02 | 0.786782061 |
| ssc04921:Oxytocin signaling pathway | 16 | 4.68E-02 | 1.258851298 |
| ssc05221:Acute myeloid leukemia | 8 | 4.92E-02 | 0.629425649 |
| ssc04010:MAPK signaling pathway | 23 | 5.00E-02 | 1.809598741 |

^1^*P*-value < 0.05 was considered as a cut-off criteria to identify significantly enriched pathways.

**Supplementary Table S6. GO enrichment analysis of DEmiRNAs after H_2_O_2_ treatment**

| **GO terms** | **GO names** | **P-value^1^** | **Gene No.** | **DEmiRNAs** |
| --- | --- | --- | --- | --- |
| [GO:0006351](https://www.ebi.ac.uk/QuickGO/term/GO:0006351) | Transcription, DNA-templated | >1.00E-17 | 729 | 9 |
| [GO:0009056](https://www.ebi.ac.uk/QuickGO/term/GO:0009056) | Catabolic process | >1.00E-17 | 594 | 9 |
| GO:0008150 | Biological_process | >1.00E-17 | 5440 | 10 |
| GO:0008543 | Fibroblast growth factor receptor signaling pathway | >1.00E-17 | 98 | 10 |
| GO:0016032 | Viral process | >1.00E-17 | 191 | 10 |
| GO:0022607 | Cellular component assembly | >1.00E-17 | 520 | 10 |
| GO:0044281 | Small molecule metabolic process | >1.00E-17 | 844 | 10 |
| GO:0005575 | Cellular_component | >1.00E-17 | 5869 | 11 |
| GO:0005829 | Cytosol | >1.00E-17 | 990 | 11 |
| GO:0019899 | Enzyme binding | >1.00E-17 | 572 | 11 |
| GO:0043903 | Symbiosis, encompassing through parasitism | >1.00E-17 | 217 | 11 |
| GO:0000988 | Protein binding transcription factor activity | >1.00E-17 | 219 | 12 |
| GO:0005654 | Nucleoplasm | >1.00E-17 | 473 | 12 |
| [GO:0031519](https://www.ebi.ac.uk/QuickGO/term/GO:0031519) | Protein complex | >1.00E-17 | 1444 | 12 |
| GO:0007173 | Epidermal growth factor receptor signaling pathway | >1.00E-17 | 123 | 13 |
| GO:0038095 | Fc-epsilon receptor signaling pathway | >1.00E-17 | 101 | 13 |
| GO:0003700 | Nucleic acid binding transcription factor activity | >1.00E-17 | 462 | 14 |
| GO:0010467 | Gene expression | >1.00E-17 | 289 | 14 |
| GO:0048011 | Neurotrophin TRK receptor signaling pathway | >1.00E-17 | 162 | 14 |
| GO:0003674 | Molecular_function | >1.00E-17 | 6304 | 15 |
| GO:0006464 | Cellular protein modification process | >1.00E-17 | 1109 | 18 |
| [GO:0009058](https://www.ebi.ac.uk/QuickGO/term/GO:0009058) | Biosynthetic process | >1.00E-17 | 1792 | 20 |
| GO:0034641 | Cellular nitrogen compound metabolic process | >1.00E-17 | 2167 | 20 |
| GO:0043167 | Ion binding | >1.00E-17 | 2680 | 20 |
| GO:0043226 | Organelle | >1.00E-17 | 4439 | 25 |
| [GO:0000278](https://www.ebi.ac.uk/QuickGO/term/GO:0000278) | Mitotic cell cycle | 1.11E-16 | 147 | 6 |
| GO:0008092 | Cytoskeletal protein binding | 1.11E-16 | 287 | 8 |
| GO:0007596 | Blood coagulation | 4.80E-14 | 190 | 10 |
| GO:0030234 | Enzyme regulator activity | 2.56E-13 | 306 | 8 |
| GO:0043687 | Post-translational protein modification | 5.91E-13 | 81 | 6 |
| GO:0048015 | Phosphatidylinositol-mediated signaling | 8.12E-13 | 67 | 9 |
| GO:0008219 | Cell death | 1.35E-12 | 308 | 6 |
| GO:0003723 | RNA binding | 4.71E-12 | 348 | 3 |
| GO:0044267 | Cellular protein metabolic process | 5.92E-12 | 140 | 4 |
| GO:0034655 | Nucleobase-containing compound catabolic process | 6.47E-12 | 277 | 7 |
| GO:0006950 | Response to stress | 7.62E-12 | 668 | 8 |
| GO:0061024 | Membrane organization | 1.95E-08 | 224 | 7 |
| GO:0006367 | Transcription initiation from RNA polymerase II promoter | 5.60E-06 | 83 | 6 |
| GO:0060012 | Synaptic transmission | 8.94E-05 | 134 | 5 |
| GO:0034330 | Cell junction organization | 2.68E-04 | 57 | 4 |
| GO:0038096 | Fc-gamma receptor involved in Phagocytosis | 3.40E-04 | 27 | 3 |
| GO:0008286 | Insulin receptor signaling pathway | 4.99E-04 | 72 | 6 |
| [GO:0007183](https://www.ebi.ac.uk/QuickGO/term/GO:0007183) | Protein complex assembly | 1.46E-03 | 165 | 5 |
| GO:0044255 | Cellular lipid metabolic process | 1.73E-03 | 52 | 5 |
| GO:0007411 | Axon guidance | 3.55E-03 | 116 | 3 |
| GO:0006259 | DNA metabolic process | 4.35E-03 | 113 | 3 |
| [GO:0007399](https://www.ebi.ac.uk/QuickGO/term/GO:0007399) | Nervous system development | 4.51E-03 | 106 | 4 |
| GO:0007267 | Cell-cell signaling | 4.93E-03 | 134 | 3 |
| GO:0038123 | Toll-like receptor TLR1:TLR2 signaling pathway | 6.21E-03 | 24 | 3 |
| GO:0038124 | Toll-like receptor TLR6:TLR2 signaling pathway | 6.21E-03 | 24 | 3 |
| GO:0034329 | Cell junction assembly | 7.47E-03 | 24 | 3 |
| GO:0018279 | Protein N-linked glycosylation via asparagine | 7.75E-03 | 37 | 4 |
| GO:0034166 | Toll-like receptor 10 signaling pathway | 8.11E-03 | 23 | 3 |
| GO:0030168 | Platelet activation | 3.10E-02 | 49 | 3 |
| GO:0006921 | Execution Phase of apoptosis | 3.12E-02 | 20 | 3 |
| GO:0035666 | TRIF-dependent toll-like receptor signaling pathway | 3.31E-02 | 24 | 4 |
| GO:0005815 | Microtubule organizing center | 3.45E-02 | 137 | 5 |
| GO:0016071 | MRNA metabolic process | 3.57E-02 | 22 | 1 |
| [GO:0100020](https://www.ebi.ac.uk/QuickGO/term/GO:0100020) | Transcription from RNA polymerase II promoter | 3.63E-02 | 109 | 3 |
| [GO:0002376](https://www.ebi.ac.uk/QuickGO/term/GO:0002376) | Immune system process | 3.92E-02 | 254 | 4 |

^1^GO terms with *P*-value < 0.05 were considered as significant functional processes of DEmiRNAs.

**Supplementary Table S7. KEGG pathway analysis of DEmiRNAs after H_2_O_2_ treatment**

| **KEGG terms** | **Pathways** | ***P*-value^1^** | **Numbers of genes** | **Numbers of DEmiRNAs** |
| --- | --- | --- | --- | --- |
| ssc05205 | Proteoglycans in cancer | 2.05E-10 | 123 | 24 |
| ssc00061 | Fatty acid biosynthesis | 8.62E-09 | 8 | 6 |
| ssc04915 | Estrogen signaling pathway | 1.47-E07 | 56 | 18 |
| ssc04550 | Pluripotency of stem cells regulation pathway | 8.46E-06 | 87 | 26 |
| ssc04390 | Hippo signaling pathway | 1.16E-05 | 88 | 23 |
| ssc05200 | Pathways in cancer | 1.46E-05 | 221 | 25 |
| ssc04360 | Axon guidance | 3.25E-05 | 77 | 21 |
| ssc04350 | TGF-beta signaling pathway | 3.90E-05 | 50 | 22 |
| ssc04068 | FoxO signaling pathway | 3.90E-05 | 83 | 24 |
| ssc04014 | Ras signaling pathway | 7.44E-05 | 128 | 24 |
| ssc04150 | mTOR signaling pathway | 8.13E-05 | 45 | 22 |
| ssc04520 | Adherens junction | 1.20E-04 | 49 | 20 |
| ssc05231 | Choline metabolism in cancer | 1.99E-04 | 66 | 23 |
| ssc04810 | Regulation of actin cytoskeleton | 1.99E-04 | 123 | 24 |
| ssc05210 | Colorectal cancer | 2.48E-04 | 41 | 22 |
| ssc05223 | Non-small cell lung cancer | 3.07E-04 | 36 | 18 |
| ssc04012 | ErbB signaling pathway | 3.07E-04 | 55 | 20 |
| ssc05215 | Prostate cancer | 3.18E-04 | 57 | 21 |
| ssc04722 | Neurotrophin signaling pathway | 3.51E-04 | 75 | 24 |
| ssc04724 | Glutamatergic synapse | 3.51E-04 | 67 | 25 |
| ssc05020 | Prion diseases | 4.89E-04 | 12 | 14 |
| ssc05212 | Pancreatic cancer | 7.23E-04 | 43 | 20 |
| ssc01212 | Fatty acid metabolism | 7.35E-04 | 24 | 14 |
| ssc05211 | Renal cell carcinoma | 7.35E-04 | 45 | 20 |
| ssc04015 | Rap1 signaling pathway | 7.35E-04 | 119 | 26 |
| ssc04310 | MAPK signaling pathway | 8.14E-04 | 125 | 18 |
| ssc04510 | Wnt signaling pathway | 1.10E-03 | 80 | 24 |
| ssc04141 | Focal adhesion | 1.16E-03 | 117 | 24 |
| ssc04151 | Protein processing in endoplasmic reticulum | 1.46E-03 | 93 | 24 |
| ssc05202 | PI3K-Akt signaling pathway | 1.66E-03 | 182 | 25 |
| ssc04727 | Transcriptional misregulation in cancer | 1.72E-03 | 94 | 23 |
| ssc05220 | GABAergic synapse | 3.70E-03 | 47 | 22 |
| ssc05214 | Chronic myeloid leukemia | 5.91E-03 | 46 | 20 |
| ssc04917 | Glioma | 6.23E-03 | 38 | 20 |
| ssc04660 | Prolactin signaling pathway | 7.18E-03 | 43 | 21 |
| ssc05221 | T cell receptor signaling pathway | 7.29E-03 | 61 | 19 |
| ssc05203 | Acute myeloid leukemia | 7.54E-03 | 35 | 19 |
| ssc04120 | Viral carcinogenesis | 7.54E-03 | 102 | 22 |
| ssc00534 | Ubiquitin mediated proteolysis | 7.54E-03 | 80 | 23 |
| ssc05213 | Heparan sulfate / heparin | 8.45E-03 | 15 | 13 |
| ssc04919 | Endometrial cancer | 8.45E-03 | 33 | 20 |
| ssc04921 | Thyroid hormone signaling pathway | 8.45E-03 | 66 | 23 |
| ssc05161 | Oxytocin signaling pathway | 8.45E-03 | 87 | 24 |
| ssc00532 | Hepatitis B | 8.45E-03 | 76 | 24 |
| ssc04152 | Glycosaminoglycan biosynthesis sulfate | 8.72E-03 | 11 | 12 |
| ssc04115 | AMPK signaling pathway | 1.09E-02 | 72 | 23 |
| ssc00510 | p53 signaling pathway | 1.15E-02 | 41 | 17 |
| ssc04144 | N-Glycan biosynthesis | 1.40E-02 | 27 | 20 |
| ssc05218 | Endocytosis | 1.43E-02 | 108 | 24 |
| ssc00512 | Melanoma | 1.60E-02 | 43 | 19 |
| ssc05032 | Mucin type O-Glycan biosynthesis | 1.80E-02 | 16 | 11 |
| ssc04024 | Morphine addiction | 2.05E-02 | 49 | 22 |
| ssc04916 | cAMP signaling pathway | 2.78E-02 | 105 | 24 |
| ssc00471 | Melanogenesis | 2.92E-02 | 55 | 22 |
| ssc04710 | D-Glutamine and D-glutamate metabolism | 3.54E-02 | 4 | 5 |
| ssc04010 | Circadian rhythm | 3.65E-02 | 22 | 18 |
| ssc04910 | Insulin signaling pathway | 3.80E-02 | 76 | 22 |
| ssc04022 | cGMP-PKG signaling pathway | 3.80E-02 | 88 | 25 |
| ssc00310 | Lysine degradation | 4.54E-02 | 24 | 17 |
| ssc05100 | Bacterial invasion of epithelial cells | 4.68E-02 | 43 | 20 |

^1^*P*-value < 0.05 was considered as a criteria for significant enriched pathway identification.

**Supplementary Table S8. Hub genes in the protein-protein interaction network**

| **Ensembl ID** | **Gene name** | **Degree^1^** | **Radiality** | **Stress** | **Regulation** |
| --- | --- | --- | --- | --- | --- |
| ENSSSCG00000007951 | CREBBP | 60 | 0.80523870 | 310326 | up |
| ENSSSCG00000021411 | HIST1H2BD | 56 | 0.80925222 | 240822 | down |
| ENSSSCG00000010214 | CDK1 | 42 | 0.80713984 | 290510 | up |
| ENSSSCG00000003949 | CDC20 | 40 | 0.77672159 | 60634 | up |
| ENSSSCG00000016958 | PIK3R1 | 37 | 0.80608365 | 272156 | up |
| ENSSSCG00000009370 | FOXO1 | 33 | 0.73848754 | 164776 | up |
| ENSSSCG00000040738 | DYNC1H1 | 33 | 0.77777778 | 75214 | up |
| ENSSSCG00000035842 | SUMO1 | 33 | 0.79763414 | 184684 | down |
| ENSSSCG00000022486 | CBL | 32 | 0.78918462 | 155200 | up |
| ENSSSCG00000016174 | FN1 | 31 | 0.79678918 | 116610 | up |
| ENSSSCG00000000078 | TNRC6B | 31 | 0.77313054 | 79046 | up |
| ENSSSCG00000034373 | TNRC6C | 31 | 0.77313054 | 79046 | up |
| ENSSSCG00000015842 | PPP2CB | 31 | 0.79721166 | 140222 | up |
| ENSSSCG00000022486 | CBLB | 30 | 0.77439797 | 74234 | up |
| ENSSSCG00000001654 | PPP2R5D | 30 | 0.79509928 | 96002 | up |
| ENSSSCG00000007458 | NCOA3 | 30 | 0.77460921 | 47532 | up |
| ENSSSCG00000015853 | HERC2 | 29 | 0.76996198 | 69910 | up |
| ENSSSCG00000004789 | THBS1 | 29 | 0.77946768 | 214174 | up |
| ENSSSCG00000018039 | NCOR1 | 28 | 0.76151246 | 75242 | up |
| ENSSSCG00000028239 | FBXL7 | 27 | 0.74165610 | 25320 | up |
| ENSSSCG00000014915 | EED | 27 | 0.73954373 | 33786 | down |
| ENSSSCG00000030632 | UBE2V1 | 27 | 0.74228982 | 24928 | down |
| ENSSSCG00000008690 | RNF4 | 27 | 0.76932826 | 66890 | up |
| ENSSSCG00000038220 | RXRA | 27 | 0.78200253 | 51042 | down |
| ENSSSCG00000008478 | SOS1 | 26 | 0.79742290 | 86706 | up |
| ENSSSCG00000005935 | AGO2 | 26 | 0.77651035 | 102496 | up |
| ENSSSCG00000029326 | CCNB1 | 26 | 0.78158006 | 101382 | down |
| ENSSSCG00000011889 | GSK3B | 25 | 0.78221377 | 122824 | up |
| ENSSSCG00000036562 | HIST3H3 | 25 | 0.77038445 | 40848 | down |
| ENSSSCG00000023998 | PSMD2 | 25 | 0.78263625 | 83740 | down |

^1^Genes with degree ≥ 25 was considered as hub genes in the protein-protein interaction network.

**Supplementary Table S9. Hub genes and miRNAs in the miRNA-mRNA interaction network**

| **DEmiRNAs** | **Regulation** | **Common DEmRNA targets^1^** | **Numbers** |
| --- | --- | --- | --- |
| ssc-miR-198 | down | *SYVN1* | 1 |
| ssc-miR-221 | down | *FOXO1, CDK6, DICER1,FOS, NR5A2, PIKER1, TEAD1, THBS1, TCF7L2, MEGF9, CBL* | 11 |
| ssc-miR-27b | down | *FOXO1, AGO2, CDK6, CREB3L2, FOXO3, CCNJ, CCNK, FZD4, GDF6, ID4, NCOA3, NR5A2, PIK3R1, SOS1, TEAD1, TGFBR3, USP31, PROX1* | 18 |
| ssc-miR-365 | down | *CBL, CHD6, MAP3K13, NCOA2, NR5A2* | 5 |
| ssc-miR-369 | down | *BMP2, EP300, FOXO1, HOXD8, NCOA2, PROX1* | 6 |
| ssc-miR-370 | down | *AR, CCNJ, SOX5, MEGF9* | 4 |
| ssc-miR-411 | down | *USP31, USP53, TCF7L2, MEGF9, MAPKBP1* | 5 |
| ssc-miR-424 | down | *FOXO1, ACVR2B, BCL9, BCL9L, BDNF, CDK6, DICER1, ELK4, ELK5, FOS, CCNJ, CHD6, MAP3K13, PIK3R1, ROCK2, SOX9, TGFBR3, TRAF6, USP31, VEGFA, MEGF9, FOSL* | 22 |
| ssc-miR-451 | down | *FZD4, FZD7, NCOA2, SOX5, VEGFA, MEGFA* | 6 |
| ssc-miR-125 | up | *SOD2, BCL2L11, IL6R, PCNA, COX-3, TRAAPC2, ZFP62, DPH2, PKIA* | 9 |
| ssc-miR-126 | up | *CDKN2D, PROCR, CTSS, PKIA* | 4 |
| ssc-miR-128 | up | *SMAD5, PTGER4, PROCR, PKIA* | 4 |
| ssc-miR-130a | up | *SMAD5, MYB* | 2 |
| ssc-miR-141 | up | *IL6R, SOD2, TRAAPC2, DIO2, SUMO1* | 5 |
| ssc-miR-142 | up | *SOD2, SMAD5* | 2 |
| ssc-miR-17 | up | *SMAD5* | 1 |
| ssc-miR-182 | up | *CCDC59, DUSP22, MYB, SERPINB2, NDRG1, MGST2* | 6 |
| ssc-miR-192 | up | *SOD2* | 1 |
| ssc-miR-193a | up | *BCL2L11, CCR1, DUSP22* | 3 |
| ssc-miR-23 | up | *CA2* | 1 |
| ssc-miR-30b | up | *BCL2L11, DIO2, PKIA* | 3 |
| ssc-miR-373 | up | *MYB, TRAAPC2* | 2 |
| ssc-miR-497 | up | *BIRC5* | 1 |

^1^The genes with colors are experimentally-verified targets. Genes in red or blue indicted the experimentally-verified miRNA-miRNA regulation pairs which were discovered in ovary tissue or non-ovary tissues, respectively
